# Imputation of fluid intelligence scores reduces ascertainment bias and increases power for analyses of common and rare variants

**DOI:** 10.1038/s41588-026-02787-5

**Published:** 2026-10-05

**Authors:** David M. van den Berg, Daniel S. Malawsky, Wei Huang, Petr Danecek, Klaudia Walter, Georgios Kalantzis, Isaac Wade, Duncan Palmer, Ewan Birney, Karin J. H. Verweij, Dirk J. A. Smit, Sarah J. Lindsay, Matthew E. Hurles, Abdel Abdellaoui, Hilary C. Martin

**Affiliations:** 1 Department of Psychiatry, Amsterdam UMC, University of Amsterdam, Amsterdam, the Netherlands; 2 Wellcome Sanger Institute, Wellcome Genome Campus, Hinxton, UK; 3 Nuffield Department of Population Health, Medical Sciences Division, University of Oxford, Oxford, UK; 4 Department of Statistics, University of Oxford, Oxford, UK; 5 The Pioneer Centre for SMARTbiomed, Big Data Institute, Li Ka Shing Centre for Health Information and Discovery, University of Oxford, Oxford, UK; 6 European Molecular Biology Laboratory, European Bioinformatics Institute (EMBL-EBI), Cambridge, UK

**Keywords:** Population genetics, Genome-wide association studies, Neurodevelopmental disorders, Sequencing

## Abstract

Studying the genetics of intelligence provides insight into the neurobiology of cognitive function and rare neurodevelopmental conditions. Here we integrated fluid intelligence (FI) tests across different UK Biobank measures and imputed FI for unmeasured individuals, increasing the sample size from ~270,000 to >455,000. We show through extensive validation that the imputed phenotype genetically resembles measured FI, although some noncognitive signal likely remains. Combining measured and imputed FI increases the number of independent significant common SNP associations from 390 to 550 and reduces the ascertainment bias introduced by FI test participants being, on average, more educated. Rare variant analyses identify 26 significantly associated genes (false discovery rate < 1%), including 8 without strong prior evidence of involvement in intelligence or neurodevelopmental conditions, which replicate in aggregate in external cohorts. Our results demonstrate that imputed intelligence can boost power for genetic discovery and support its use to help discover new neurodevelopmental condition-associated genes.

## Abstract (web summary)

Imputing unmeasured fluid intelligence scores in UK Biobank reduces ascertainment bias and increases power for genetic analyses, improving out-of-sample polygenic score performance and identifying rare variant signals that are strongly enriched for intellectual disability-associated genes.

## Main

Measures of intelligence are associated with many life outcomes, including educational attainment (EA)^1^, occupational success^2^, income^3^, and mental^4,5^ and physical health^6,7^. Understanding the genetic architecture of intelligence can help illuminate the biological basis of cognitive functioning and the etiology of rare neurodevelopmental conditions, which are negatively genetically correlated with measures of intelligence^8^. It can also facilitate better-powered studies into the role of intelligence in socioeconomic and health-related disparities.

Pedigree-based studies have shown that genetics makes a substantial contribution to variation in measures of intelligence^9–11^, with the heritability of ‘general intelligence’ estimated at ~0.6 to 0.8 from twin and sibling-regression studies^12,13^. The largest genome-wide association study (GWAS) meta-analysis of intelligence measures to date^14^ (*n* = 269,867; 73% UK Biobank (UKB), 13% COGENT consortium^15^, 14% 12 other cohorts), conducted in ref. ^14^, reported 205 associated genomic loci and an SNP heritability of 0.19 (ref. ^16^). A recent within-family GWAS meta-analysis conducted in ref. ^17^ estimated direct genetic effects on measures of intelligence within families, thereby removing biases from genetic nurture, residual population stratification and assortative mating. Their results suggested that common genetic variants contribute substantially to variation in measures of intelligence both within and between families but that the population-based GWAS results may be biased by residual confounding.

Recent studies have shown that rare protein-truncating variants (PTVs) and damaging missense variants in genes intolerant of loss of function^18^ are associated with lower scores on intelligence tests in UKB^19,20^; such variants are also implicated in rare developmental disorders (DDs)^21,22^. Seven genes are identified in ref. ^20^ in which rare PTVs are negatively associated with measures of intelligence^20^, of which three are established DD-associated genes^20^. The rare variants in these genes have substantially larger effect sizes on the measures of intelligence used (~0.25 to 1 s.d.) than common variants implicated through GWAS (typically <0.1 s.d.)^23^. Increasing sample sizes in rare variant analyses should enable the discovery of additional genes associated with measures of intelligence.

The intelligence measure in UKB that these studies used is a measure of verbal-numerical reasoning commonly referred to as ‘fluid intelligence’ (FI), the ability to solve new problems independent of prior knowledge^24^. It was measured using a 13-item or 14-item survey at the baseline in-person visit and in subsequent online questionnaires completed at different times (Supplementary Note 1, Supplementary Table 1 and Supplementary Fig. 1). This FI test has a test–retest correlation of ~0.6 (Supplementary Table 2) and correlates ~0.55 with a measure of ‘general cognitive ability’ estimated from gold-standard cognitive tests^25^. To date, ~62% of UKB participants have completed the FI test at least once, but previous studies have analyzed only a subset of these participants. Participants who completed FI tests tended to have higher EA and experience lower socioeconomic deprivation within UKB (Supplementary Fig. 2b). These kinds of selection biases have been shown to distort genetic associations, affecting downstream estimates of heritability and genetic correlations^26^.

Inspired by recent work in major depressive disorder^27^, we sought to integrate FI values across the various FI tests in UKB and impute missing values for the 38% of individuals of European ancestry who did not complete any of them. We predicted that this would ameliorate ascertainment bias and boost power for downstream genetic analyses. We developed a theoretical framework (Supplementary Note 2) that formalizes the conditions under which pooled analysis of measured and imputed phenotypes recovers consistent population genetic effects and provides principled validation criteria based on heritability and genetic-correlation estimates. We tested multiple approaches to integrate all FI tests and impute missing FI scores (FIS) and evaluated these with the derived validation criteria using population and within-family GWAS. Then, we used the integrated and imputed FIS to perform the largest common-variant GWAS for intelligence to date (*n* = 490,700) and additionally boost power for rare variant-based analyses (*n* = 438,285). For a less technical description of the paper and ethical considerations, see the general lay summary and frequently asked questions (FAQ) document^28^. See Table 1 for an overview of terminology used in this paper.

**Table 1:** Glossary of abbreviations and terms

Abbreviation	Description
Intelligence^14^	GWAS meta-analysis of measures of intelligence in 14 cohorts, including UKB, from ref. ^14^
COGENT	GWAS meta-analysis of measures of intelligence across 24 cohorts in the COGENT consortium from ref. ^15^. No UKB participants included.
NonCogEA	GWAS of the ‘noncognitive component’ of EA, derived using GWAS-by-subtraction in ref. ^30^
FIS	FIS in UKB
FIS1	FIS measured at baseline (20016–0.0)
Earliest FIS	Earliest measured FIS across five measured tests (Supplementary Table 1)
Average FIS	Average measured FIS across five measured tests (Supplementary Table 1)
Observed FIS	Term used in the main text to describe ‘average FIS’ in downstream analyses
Imputed FIS	Term used in the main text to describe imputed FIS produced by using average FIS in the imputation along with 82 other variables
Combined FIS	Mega-analysis of observed and imputed FIS (named MegaFIS-average in supplement)

## Results

### Integration of measured fluid intelligence phenotypes

**Extended Data Fig. 1:**
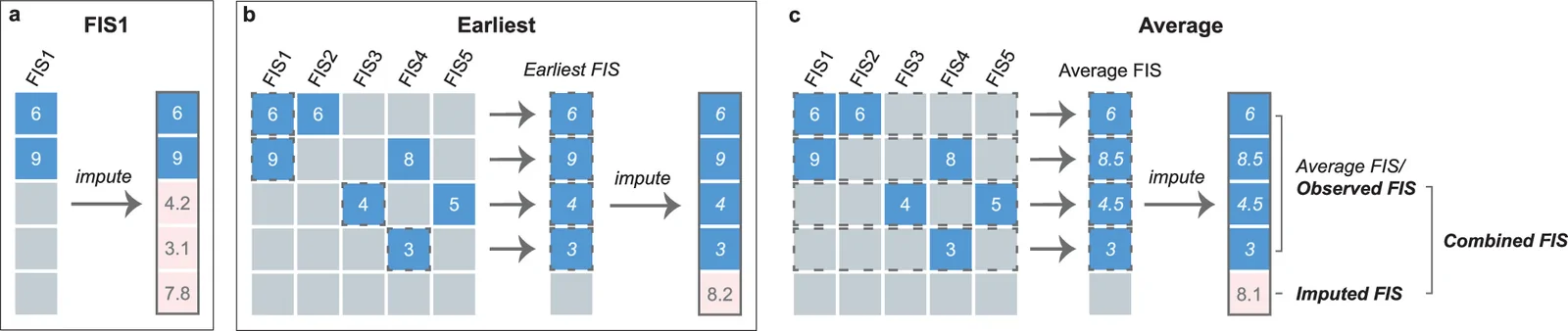
Schematic illustrating the strategies for integration and imputation of fluid intelligence described in the main text. **a**, Missing values (gray) are imputed (pink) using observed (blue) FIS1 and selected imputation variables. **b**, Five FIS are integrated by taking the earliest available measure, which is then used for imputation along with selected imputation variables. **c**, Our selected approach: five FIS are integrated by taking the average of available measures, which is then used for imputation along with selected imputation variables. Nomenclature used throughout this study is highlighted in bold. Although raw FIS scores are shown for clarity, actual scores were transformed before imputation (Methods). See Supplementary Fig. 5 for additional integration strategies evaluated in Supplementary Note 3.

We first set out to integrate FIS measured at five different time points (Supplementary Note 1, Supplementary Table 1 and Supplementary Fig. 1). After adjusting for age and sex (Methods), we evaluated the following two approaches for integrating the transformed FIS values (Extended Data Fig. 1): (1) taking the average of available scores (average FIS), and (2) taking the first available score (earliest FIS). We compared these approaches to using only the FI test taken at the initial assessment visit (FIS1). To assess the characteristics of these three phenotypes, we conducted population-based GWAS using REGENIE with heteroskedasticity-robust standard errors (Methods; Supplementary Note 2). We then considered the SNP heritability (*h*^2^_pop_) and genetic correlations (*r*_g_) with a range of traits (Supplementary Fig. 3 and Supplementary Table 3). In particular, we examined genetic correlations with two previous GWAS of measures of intelligence^14,15^ and, as negative controls, GWAS for height^29^ and the noncognitive component of educational attainment (NonCogEA)^30^. The NonCogEA GWAS was derived by ‘subtracting’ the association signal from a cognition GWAS (a meta-analysis of the COGENT GWAS^15^ with a GWAS of FIS in UKB) from the GWAS of EA in ref. ^31^. NonCogEA therefore reflects factors other than cognitive ability that contribute to EA, which we want to minimize in a GWAS for either observed or imputed FI, as well as potentially aspects of cognitive ability that are not measured by the UKB FI test or the COGENT cognition tests.

**Fig. 1:**
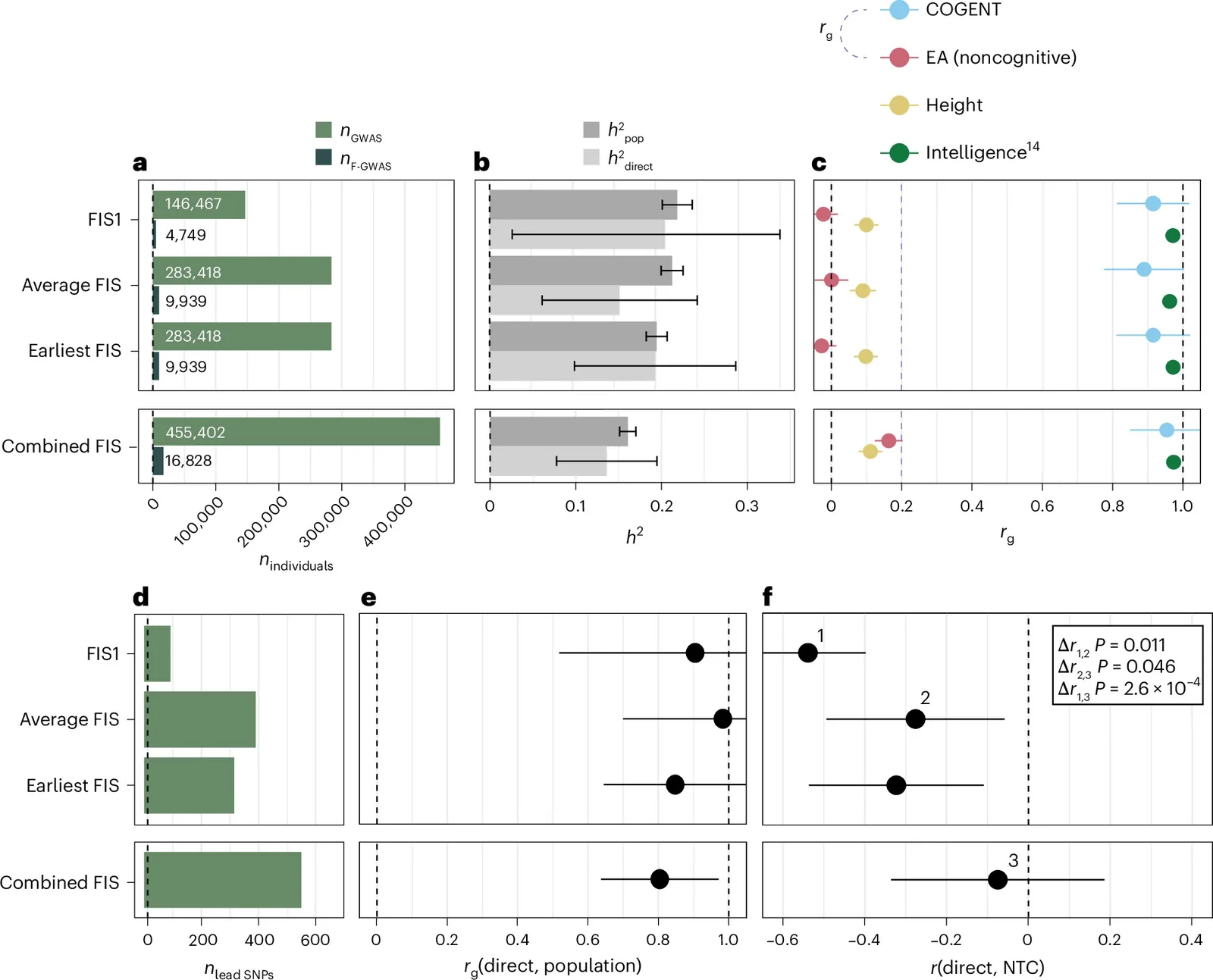
Evaluation of different data integration and imputation approaches. All panels give various summary statistics from either population-based or within-family GWAS of measured FI phenotypes (top three results) and through mega-analysis of average measured and imputed FIS (‘combined FIS’; bottom result). Association was tested through linear regression using REGENIE and SNIPAR, both using two-sided *z* tests. **a**, Sample sizes of population GWAS (*n*_GWAS_) and within-family GWAS (*n*_F-GWAS_). **b**, Population and direct effect heritabilities estimated using LDSC^70^. **c**, Population effect genetic correlations estimated using LDSC^71^ with traits from external studies of measures of intelligence (blue and green), the NonCogEA (red) and height (yellow). The purple dashed line indicates the genetic correlation between the COGENT GWAS and NonCogEA. **d**, Number of significant (*P* < 5 × 10^−8^) independent lead SNPs from the population-based GWAS computed using FUMA^32^. **e**, Genome-wide correlations estimated using LDSC between the population effects and the direct effects from a within-family GWAS conducted on the indicated trait. **f**, Correlations between direct effects and NTCs estimated using SNIPAR^33^ for the indicated trait. Inset *P* values indicate the significance of two-sided *z* tests for pairwise differences across estimates. Standard errors were obtained from a block-jackknife procedure. *P* values were not adjusted for multiple comparisons. For **b**, **c**, **e** and **f**, display point estimates ± 95% CI (estimate ± 1.96 × s.e.).

Integrating the phenotypes using the average and earliest FIS approaches increased the sample size from 146,467 (FIS1) to 283,418 individuals (Fig. 1a). Accordingly, the number of FIS-associated lead SNPs identified using functional mapping and annotation of genome-wide association studies (FUMA)^32^ increased from 92 for FIS1 to 390 and 315 for the average and earliest FIS approaches, respectively (Fig. 1d). The estimated *h*^2^_pop_ for FIS1 was 0.23 (s.e. = 0.01), which was marginally higher than that estimated for average FIS (0.22, s.e. = 0.007) and earliest FIS (0.20, s.e. = 0.006; Fig. 1b). There were no significant differences between the integrated FIS and FIS1 in terms of their patterns of genetic correlation with measured intelligence, height or NonCogEA (Fig. 1c), suggesting GWAS of these measures captured similar signals to FIS1.

To further characterize the genetic architecture of these different FI phenotypes, we conducted within-family GWAS^33^. Integrating FIS scores increased the sample size from 4,749 trios (for FIS1) to 9,939 trios. This within-family GWAS allowed us to separate direct genetic effects from other factors that can contribute to the association signal in population-based GWAS by combining them in the so-called ‘nontransmitted coefficient’ (NTC)^33^. This coefficient captures the association between nontransmitted alleles in the parents and the offspring’s phenotype, which reflects some combination of genetic nurture (that is, indirect genetic effects due to genetically influenced traits in relatives that influence the offspring’s phenotype), assortative mating and residual population stratification.

For FIS1, the linkage disequilibrium score regression (LDSC)-SNP heritability attributed to direct genetic effects (Fig. 1b) was not significantly different from the population GWAS heritability estimate (*h*^2^_direct_ = 0.21, s.e. = 0.094; *h*^2^_pop_ = 0.23, s.e. = 0.009) and was concordant with prior estimates (*h*^2^_direct_ = 0.19, s.e. = 0.027)^17^. Earliest FIS had a similarly high *h*^2^_direct_, whereas average FIS had a lower, but not significantly different, point estimate. We found that the genetic correlation between the direct effects and the corresponding population effects was not significantly different from 1 in any of these GWASs (Fig. 1e).

Next, we considered the correlation between the direct effects and NTCs (*r*(direct,NTC)). A negative correlation has previously been observed for various phenotypes including FI in UKB, potentially due to ascertainment bias^33^ (Supplementary Note 2). We replicated this for FIS1 (Fig. 1f). We hypothesized that including a larger fraction of UKB by integrating the various FI tests would ameliorate the ascertainment bias and hence attenuate this negative correlation. In line with this, when using average or earliest FIS, the *r*(direct,NTC) estimates were closer to 0 (*r* = −0.28 and *r* = −0.32, respectively) than they were for FIS1 (*r* = −0.54; Δ_average FIS_ = 0.26, *P* = 0.011), although still significantly negative (Fig. 1f). Collectively, these results suggest that, despite the slight differences between the various FIS measurements (Supplementary Note 1), phenotype integration yielded a trait that is highly genetically similar to FIS1 while reducing ascertainment biases.

### Imputation of fluid intelligence scores

We then set out to impute missing FIS for individuals who never took the FI test by leveraging correlated variables to expand the sample size for genetic analyses and reduce artifacts in downstream analysis that might arise from residual biased ascertainment. In Supplementary Note 2, we consider the circumstances under which such a strategy could, in theory, produce consistent genetic effect estimates and describe how it should be formally evaluated. In brief, we show that consistent estimates can be recovered through mega-analysis of observed and imputed values if two key assumptions hold—first, that measurement error in the observed values is uncorrelated with genotype; and, second, that the imputed values are calibrated to the intended FI construct in the unmeasured group, in the sense that they recover its mean and that their covariance with each SNP equals the covariance that the FI construct itself would have with that SNP in the unmeasured group (Supplementary Notes 2 and 4). Intuitively, the second condition requires that the imputation does not import genetic signals from the auxiliary variables that are not also present in the intended FI construct. Although these assumptions cannot be tested directly, a necessary consequence is that the genetic-correlation profile obtained from the resulting mega-analysis should be unchanged relative to that obtained from an independent GWAS targeting the same construct.

To arrive at a robust imputation strategy, we went through several iterations. We started with refining our set of imputation variables, beginning with 154 variables phenotypically correlated with intelligence that were then subsetted to 82 variables whose genetic correlation profile was more aligned with intelligence than with the residual noncognitive signal observed in the first imputation iteration (Methods). Then, we evaluated three approaches to imputing FIS (Supplementary Fig. 5), namely, through imputing FIS using the earliest FIS measure for observed individuals, the average measure for observed individuals, or all available FIS measures. These approaches were evaluated using imputation accuracy, SNP heritability (population based and within family), genetic correlations with external traits and the correlation between direct effects and NTCs.

After extensive evaluation (Supplementary Figs. 4 and 6 and Supplementary Notes 2–5), we selected the approach based on imputation of average FIS to take forward for downstream analyses. Imputed average FIS showed a genetic correlation of 0.74 (s.e. = 0.015) with its observed counterpart (potentially reflecting a mixture of ascertainment bias in the observed sample^26^ and the importation of some noncognitive signal) and 0.85 (s.e. = 0.056) with COGENT. The phenotypic accuracy of imputation was assessed in a separate analysis based on the correlation between observed and imputed FIS for a set of randomly chosen participants (Supplementary Table 5), whose measured values were removed before imputation. Our chosen strategy achieved an accuracy of *r* = 0.46 (s.e. = 0.004), slightly lower than the test–retest correlation of FI we found in UKB (Supplementary Table 2; *r* = 0.6).

**Extended Data Fig. 2:**
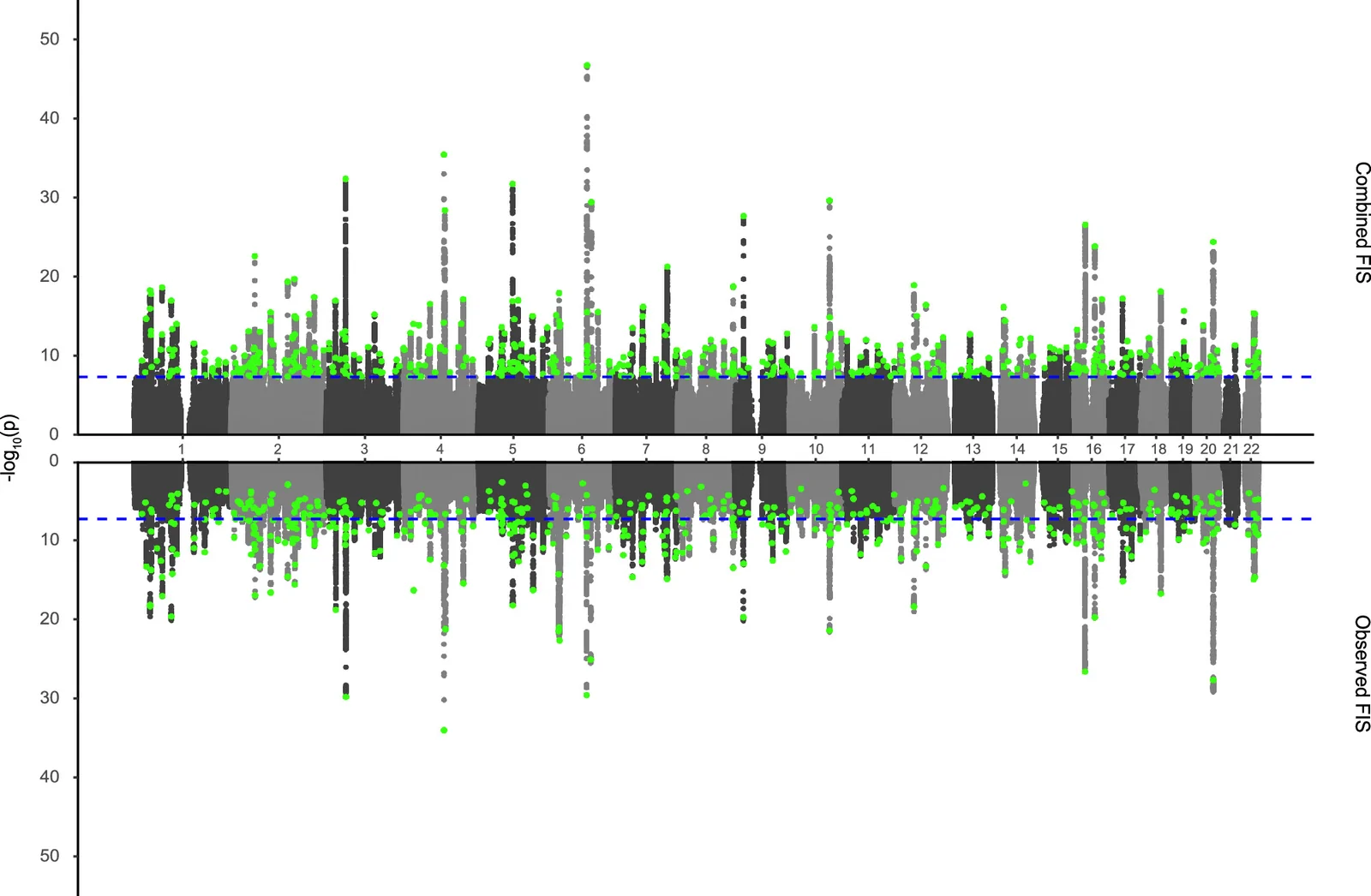
Combined FIS versus observed FIS Miami plot. Results from the population-based GWAS of combined FIS (top) versus observed FIS (bottom). SNPs highlighted in green are lead SNPs identified in combined FIS using FUMA. The *x*-axis shows the chromosomes, and the *y*-axis the −log_10_(*P*) for the SNP associations. Association was tested using linear regression, with two-sided tests of association. The dashed line in both plots indicates the genome-wide significance threshold *P* < 5 × 10^−8^. For observed FIS, highlighted SNPs below the dotted line (nonsignificant) indicate SNPs that were identified as lead SNPs as a result of adding imputed values in combined FIS.

**Extended Data Fig. 3:**
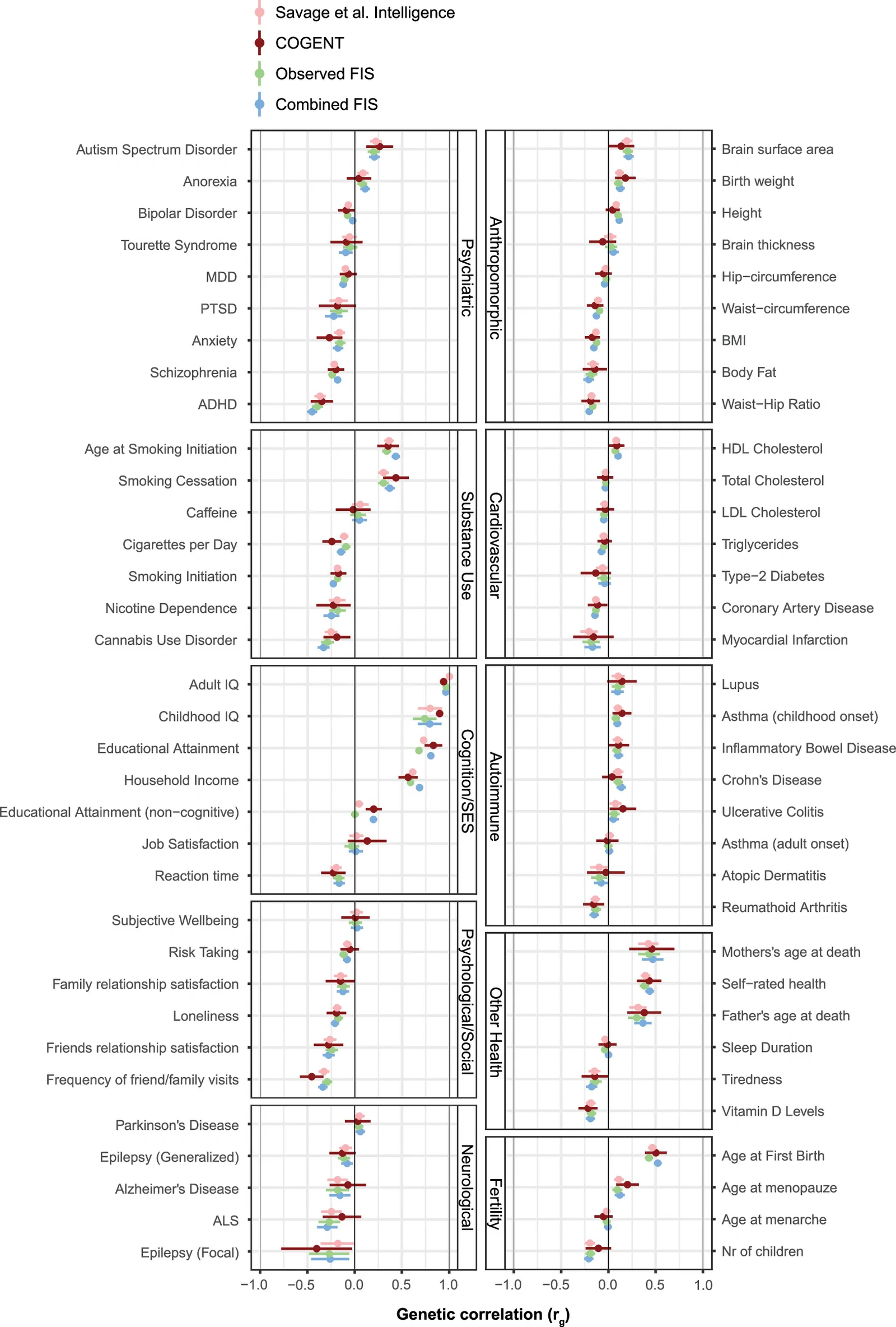
Genetic correlations of 68 traits with four intelligence GWASs. The intelligence GWASs used were those in ref. ^14^ (*n* = 269,867), the COGENT consortium (*n* = 35,298), and those based on observed FIS (*n* = 283,418) and combined FIS (*n* = 455,402) in UKB. Error bars show the 95% confidence intervals (estimate ± 1.96 × s.e.) for each point estimate. See Supplementary Table 3 for numeric values and information about the source GWASs. The sign of the *r*_g_ was flipped for self-rated health, job satisfaction, family relationship satisfaction, friends relationship satisfaction, and frequency of friend/family visits so that positive values consistently reflect a more favorable outcome.

Theory predicts that, unlike inverse-variance weighted meta-analysis, mega-analysis of sufficiently well-imputed phenotypes recovers population genetic effects even when the trait of interest correlates with the probability of having a measurement (Supplementary Notes 2 and 4). Thus, we combined the imputed measure with observed average FIS (henceforth ‘observed FIS’) in a mega-analysis that we will henceforth call ‘combined FIS’. This increased the sample size to 455,402 individuals while maintaining an LDSC-SNP heritability estimate (*h*^2^_pop_ = 0.16, s.e. = 0.005) comparable to that of observed FIS, with minimal attenuation of *h*^2^_direct_ relative to *h*^2^_pop_ (Fig. 1b). Notably, it also resulted in 550 lead SNPs, compared to only 390 obtained using observed FIS (Fig. 1d and Extended Data Fig. 2). The genetic-correlation profile of combined FIS was highly similar to that of the COGENT GWAS (Extended Data Fig. 3 and Supplementary Fig. 3), including consistent genetic correlations with height and NonCogEA, showing that this necessary condition of the imputation was approximately met (Supplementary Note 2). Additionally, the point estimate for *r*(direct, NTC) was closer to zero than was observed using observed FIS alone (Δ = −0.201, *P* = 0.046) and was not significantly different from zero (Fig. 1f), suggesting that imputation further ameliorated ascertainment bias. However, we note that combined FIS showed slightly higher genetic correlation with EA, NonCogEA and household income than observed FIS (Fig. 1c and Extended Data Fig. 3), indicating that some residual noncognitive signal (for example, socioeconomic status) was probably introduced through imputation. Nonetheless, the close correspondence with COGENT suggests that combined FIS represents a reasonable approximation of the cognitive construct measured by intelligence GWAS, and our results below suggest it is useful despite its limitations.

### Improved power for common-variant analyses of intelligence

**Fig. 2:**
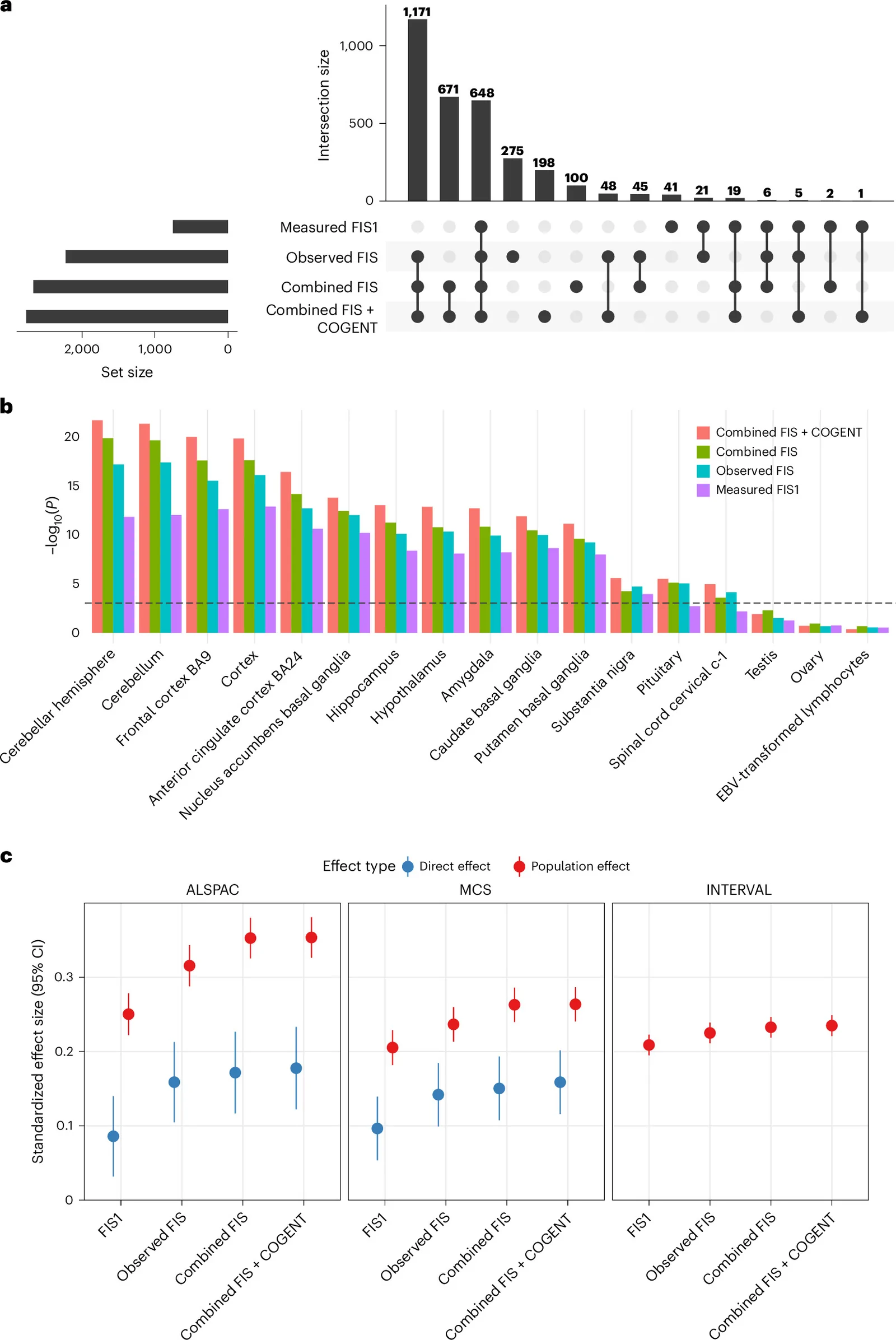
Integration and imputation increase power for downstream analyses of GWAS results. We compare the results of GWASs for FIS1 (*n* = 146,467), observed FIS (*n* = 283,418), combined FIS (*n* = 455,402) and combined FIS + COGENT (*n* = 490,700). **a**, An UpSet plot showing the overlap of protein-coding genes prioritized by each GWAS using MAGMA (FDR < 1%). **b**, −log_10_*P* values for enrichment analyses of MAGMA-prioritized genes among 54 GTEx (v8) tissues using FUMA. MAGMA gene-property analysis was performed using gene-based *P* values and tested one sided (greater), conditioning on average expression across all tissue types. The dashed line indicates a Bonferroni-corrected −log_10_*P* threshold of 0.05 (54 tissues tested). The tissues shown are those that passed Bonferroni correction, as well as three others (testis, ovary, Epstein–Barr virus (EBV)-transformed lymphocytes) for comparison (see full results in Supplementary Table 10). **c**, Associations between PGIs constructed with summary statistics from the various GWAS and cognitive measures in three independent cohorts. Points show standardized effect sizes with lines indicating 95% CI (estimate ± 1.96 × s.e.). In ALSPAC, we used full-scale IQ measured at age 8 years (*n* = 5,283). In MCS, we used a cognitive performance measure derived from a factor analysis using a combination of various cognitive tests administered across childhood (age = 3–7 years; *n* = 5,621). In INTERVAL, we used the average of the 2 FI tests administered 24 and 48 months after recruitment (*n* = 20,328). The population effects in red indicate results from a regression of the PGIs on the cognitive ability measure, controlling for sex and 20 PCs. The direct effects were estimated in the two birth cohorts by additionally controlling for parental PGIs in the regressions.

To boost power further, we meta-analyzed combined FIS with the COGENT GWAS (combined FIS + COGENT). This revealed 620 lead SNPs, while the LDSC-SNP heritability remained 0.16 (s.e. = 0.005). Across GWAS of FIS1 (*n* = 146,467), observed FIS (*n* = 283,418), combined FIS (*n* = 455,402) and combined FIS + COGENT (*n* = 490,700), larger GWAS sample sizes consistently improved gene prioritization (Fig. 2a and Supplementary Table 8) and brain-tissue enrichment (Fig. 2b and Supplementary Table 10). Notably, polygenic index (PGI) prediction of measured cognitive ability in three independent cohorts showed that both the variance explained by the PGIs (Fig. 2c and Supplementary Table 9) and direct effect estimates obtained within families increased monotonically with sample size (Supplementary Note 6).

### Improved power for rare variant analyses of FI

As expected from previous work^20^, the burden of rare (minor allele frequency (MAF) < 0.001%) PTVs and damaging missense variants was negatively associated with imputed FIS (Supplementary Note 7 and Supplementary Figs. 10 and 11). Using combined FIS reduced the size of the confidence intervals compared to using observed FIS alone (Supplementary Figs. 10 and 11), while not significantly changing the effect size for these two classes of variants. This motivated us to carry out gene-based rare variant association analyses on combined FIS to take advantage of the larger sample size.

We performed gene-based rare variant association tests with both observed FIS (*n* = 273,091) and combined FIS (*n* = 438,285) using REGENIE^34^. We used both burden tests (which assume all included variants in the gene have the same effect size) and SKAT-O (which allows them to have different effect sizes, including different directions of effect) and applied these to three combinations of rare (MAF < 0.001%) variants—PTVs alone, damaging missense alone and PTV + damaging missense combined (Supplementary Table 11).

**Fig. 3:**
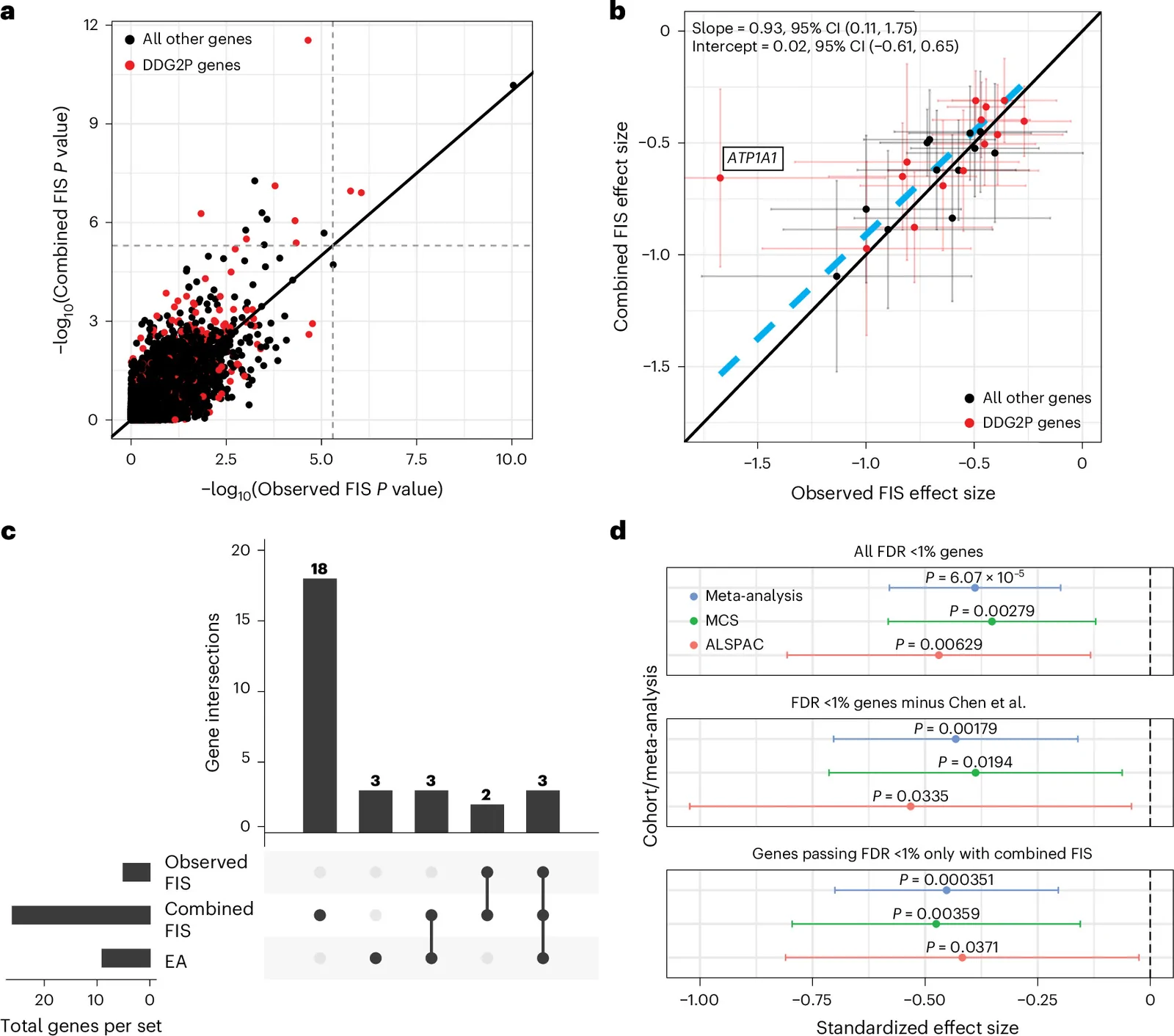
Results from gene-based rare variant association tests. **a**, −log_10_*P* obtained with observed FIS versus combined FIS from the burden test on PTVs (two-sided *t* test). **b**, Effect sizes and 95% CI (estimate ± 1.96 × s.e.) obtained with observed FIS (*n* = 273,091) versus combined FIS (*n* = 438,285) from the burden test on PTVs, for 26 genes passing FDR < 1%. The dashed blue line shows the line of best fit from a Deming regression, which takes into account the s.e. of the effect size estimates. **c**, An UpSet plot showing the overlap of protein-coding genes prioritized by rare variant analyses of observed FIS, combined FIS and EA (*n* = 434,146) at FDR <1%. **d**, Standardized effect sizes and 95% CI (estimate ± 1.96 × s.e.) for the association between PTV burden in various gene sets in ALSPAC, MCS and an inverse-variance weighted meta-analysis of both cohorts (two-sided *t* test). All meta-analysis estimates were statistically significant after Bonferroni correction (*P* < 0.05/3). ‘All FDR < 1% genes’ are the 26 genes that were significantly associated with observed and combined FIS, as detailed in Table 2. ‘FDR < 1% genes minus Chen et al.’ are the genes in the all FDR < 1% genes, excluding those that were also found to be associated at FDR < 5% with any cognitive measure assessed in ref. ^20^ (18 genes). ‘Genes passing FDR < 1% only with combined FIS’ are the genes in the all FDR < 1% genes that were only significant after additionally considering combined FIS rare variant analyses (21 genes). Gray dashed lines in **a** indicate the empirical *P-*value cutoff for an FDR < 1% in our gene association tests (*P* < 5.03 × 10^−6^).

**Extended Data Fig. 4:**
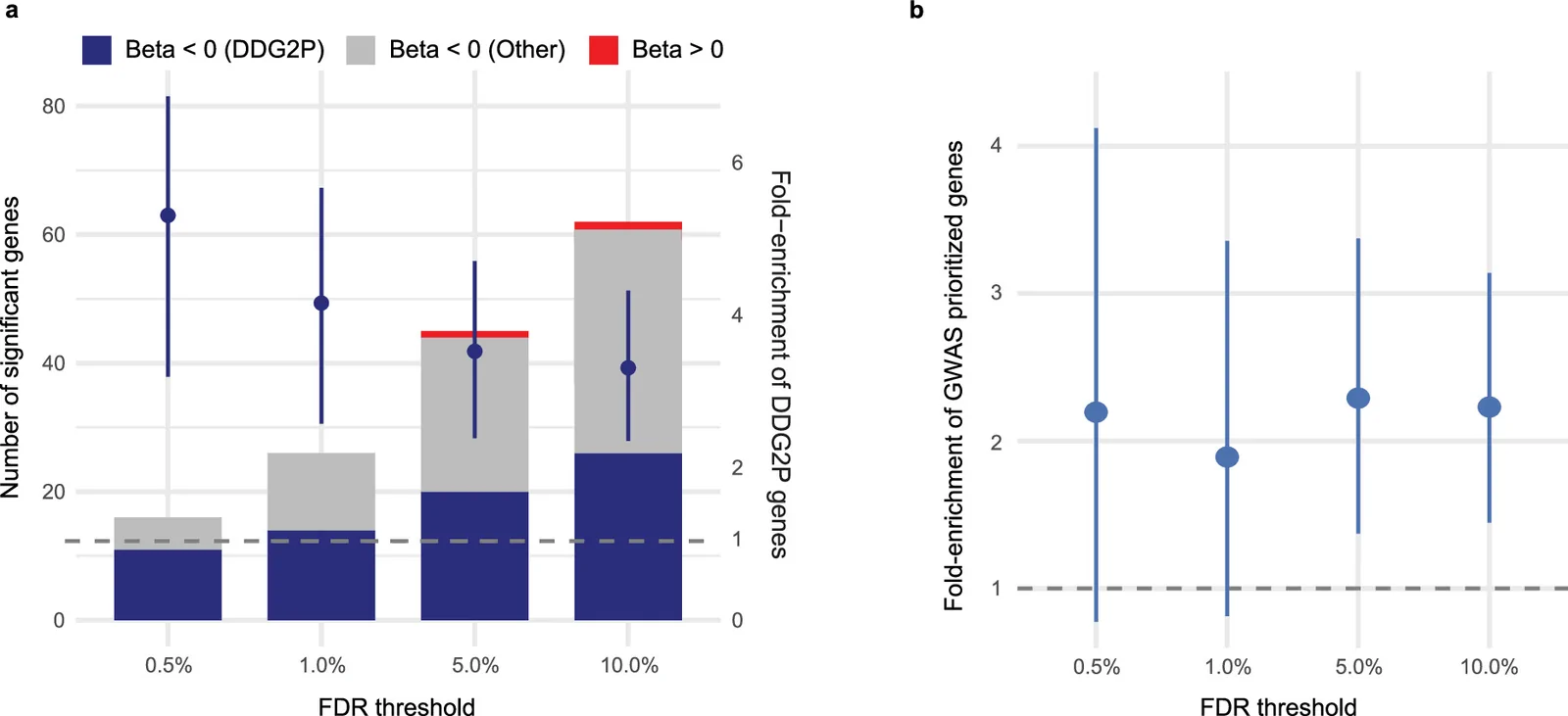
Assessing overlap between developmental disorder-associated genes and those prioritized by combined FIS + COGENT GWAS versus genes identified by rare variant burden tests. **a**, Stacked bar plots showing the number of genes significant at each rare variant FDR threshold. Dark blue segments are genes with PTV and PTV + MIS burden beta < 0 that are in the DDG2P list; gray segments are genes with PTV and PTV + MIS burden beta < 0 that are not in DDG2P; red segments are all other significant genes. Overlaid are point estimates and 95% CI (estimate ± 1.96 × s.e.) from a hypergeometric test for the fold-enrichment of DDG2P membership in each FDR-thresholded set relative to the genome-wide background, calculated as (|FDR gene set and DDG2P|/|FDR gene set|)/(|DDG2P|/|all genes|). **b**, Fold-enrichment of genes (95% CIs) implicated by the rare variant association tests at the indicated FDR threshold among MAGMA-prioritized genes passing the 1% FDR threshold from the combined FIS + COGENT GWAS (*n* = 490,700, as in Fig. 2a).

As expected, given the larger sample size, most genes were more significant using combined FIS (Fig. 3a). Among the top genes (false discovery rate (FDR) < 1%), effect sizes were highly correlated between observed and combined FISs, suggesting minimal biases being introduced by the imputation, with only one gene showing a notable difference in effect size (attenuation) after adding in imputed FIS (*ATP1A1*, which causes a dominant intellectual disability syndrome^35^; Fig. 3b). SKAT-O tended to improve power compared to burden tests (Supplementary Fig. 12). Twenty-six genes passed FDR < 1% (corresponding to *P* < 5.03 × 10^−6^; Table 2 and Supplementary Table 12), of which all had negative effects on FIS. Fourteen of these (54%) are known DD-associated genes (defined as in the DDG2P knowledge base), all causing syndromes with neurodevelopmental features, with varying modes of inheritance (Supplementary Note 8). This is a significant enrichment given that only 2,047 (13%) of 15,806 protein-coding genes we tested are in DDG2P (fold enrichment = 4.18; Fisher’s exact test, *P* = 8 × 10^−8^; Extended Data Fig. 4a).

**Table 2:** Results of gene-based rare variant tests from REGENIE

Gene	Details of most significant test				Note
	Test type	*n* carriers	*β*	*P* value	
*IGF1R*	Combined; PTV; SKAT-O	57 (27)	NA	6.9 × 10^−13^	DDG2P monoallelic and biallelic LoF^40^
*ANKRD12*	Combined; PTV; burden	154 (74)	−0.499	6.8 × 10^−11^	Associated with EA (FDR < 5%) and VNR (Bonferroni significant)^20^; paralog of DDG2P gene *ANKRD11*
*KCNMA1*	Observed; PTV + missense; burden	108	−0.523	9.6 × 10^−11^	DDG2P monoallelic altered function^41^ and biallelic LoF^42^
*CACNA1A*	Combined; PTV; SKAT-O	47 (29)	NA	1.9 × 10^−9^	DDG2P monoallelic altered function^43,44^; associated with VNR (Bonferroni significant)^20^
*TNRC6B*	Combined; PTV; SKAT-O	61 (30)	NA	5.1 × 10^−8^	DDG2P monoallelic LoF^45^
*ADGRB2*	Combined; PTV; burden	71 (35)	−0.622	5.4 × 10^−8^	Associated with EA (Bonferroni significant)^20^
*KDM5B*	Combined; PTV; SKAT-O	219 (111)	NA	6.1 × 10^−8^	DDG2P monoallelic and biallelic LoF^46^; associated with EA, RT and VNR (Bonferroni significant)^20^
*GIGYF1*	Combined; PTV; burden	103 (64)	−0.505	7.7 × 10^−8^	DDG2P monoallelic LoF^47^; associated with EA (Bonferroni significant)^20^
*ATP1A1*	Combined; PTV + missense; SKAT-O	122 (71)	NA	2.6 × 10^−7^	DDG2P monoallelic altered function^48^
*TLK1*	Combined; PTV; SKAT-O	28 (15)	NA	2.8 × 10^−7^	De novo mutations reported in two neurodevelopmental condition patients^49^; same family as *TLK2*, a DDG2P gene
*SLC8A1*	Combined; PTV; burden	19 (9)	−1.096	5.1 × 10^−7^	Associated with EA (Bonferroni significant)^20^
*SPTBN2*	Combined; PTV; burden	139 (75)	−0.403	5.3 × 10^−7^	DDG2P monoallelic altered function^50^ and biallelic LoF^51^
*PPFIA3*	Combined; PTV; burden	23 (15)	−0.972	8.8 × 10^−7^	DDG2P monoallelic altered function^52^
*MYCBP2*	Combined; PTV + missense; burden	169 (107)	−0.302	1.2 × 10^−6^	DDG2P monoallelic LoF (limited evidence)^53^
*IPO9*	Combined; PTV; SKAT-O	25 (17)	NA	1.3 × 10^−6^	No clear link to neurodevelopmental conditions
*CACNA1E*	Combined; PTV; SKAT-O	41 (24)	NA	1.4 × 10^−6^	DDG2P monoallelic altered function^43,44^
*ANK3*	Combined; PTV; burden	76 (40)	−0.524	1.7 × 10^−6^	Implicated in ID through both recessive and de novo variants^54,55^; GWAS hit for bipolar disorder^56^
*RB1CC1*	Combined; PTV; SKAT-O	47 (22)	NA	2.0 × 10^−6^	Duplications associated with schizophrenia^57^
*RC3H2*	Combined; PTV; burden	32 (18)	−0.796	2.1 × 10^−6^	Associated with VNR (Bonferroni significant)^20^
*DPP8*	Combined; PTV + missense; burden	62 (33)	−0.507	2.3 × 10^−6^	No clear link to neurodevelopmental conditions
*CHD9*	Combined; PTV; SKAT-O	78 (44)	NA	3.6 × 10^−6^	De novo missense mutations reported in autism^58–60^; in the same family of chromatin remodelers as *CHD7* (ref. ^61^) and *CHD8* (ref. ^62^), well-known neurodevelopmental condition genes
*BCAS3*	Combined; PTV; SKAT-O	69 (38)	NA	3.8 × 10^−6^	DDG2P biallelic LoF^63^; associated with EA (Bonferroni significant)^20^
*NF1*	Combined; PTV; SKAT-O	121 (69)	NA	4.01 × 10^−6^	DDG2P monoallelic LoF^64^
*ROBO2*	Combined; PTV; burden	49 (26)	−0.621	4.7 × 10^−6^	Recurrent deletions reported in autism^65^; GWAS hit for expressive vocabulary in infancy^66^
*GABRA2*	Combined; PTV + missense; SKAT-O	80 (49)	NA	4.8 × 10^−6^	DDG2P monoallelic altered function^67^
*PCLO*	Observed; PTV; burden	45	−0.706	4.9 × 10^−6^	Involved in synaptic transmission; GWAS hit for depression^68^ and post-traumatic stress disorder^69^

For genes passing FDR < 1%. Multiple tests were conducted per gene (two-sided *t* test for gene burden associations and nondirectional omnibus test for SKAT-O), and the full results are given in Supplementary Table 12. Further discussion of the associated genes is in Supplementary Note 8. This table gives the results of the most significant test for each gene. The ‘test type’ column indicates whether the test was on observed or combined FIS, whether it considered PTVs alone or PTV + missense variants and whether it was a SKAT-O or burden test. The *β* indicates the associated change in s.d. of the phenotype given one qualifying variant in the gene. Please note that SKAT-O does not produce an effect size. The ‘*n* carriers’ column indicates the number of individuals carrying a rare variant in the most significant test; for genes for which the most significant test was using combined FI, the number in parentheses is the number of carriers with an observed FI measure.

LoF, loss of function; ID, intellectual disability; EA, educational attainment; RT, reaction time; VNR, verbal-numerical reasoning.

The following five genes passed FDR < 1% using observed FIS alone (Fig. 3c): *ANKRD12*, *PCLO*, *KDM5B*, *TNRC6B* and *KCNMA1*, of which the latter three are DDG2P genes. *ANKRD12* and *KDM5B* were previously identified in a similar UKB study on cognitive phenotypes^20^ (Supplementary Note 7). An additional 21 genes passed FDR < 1% only when analyzing combined FIS, of which 11 are DDG2P genes, and 2 additional genes (*RC3H2* and *SLC8A1*) were also reported in ref. ^20^. Among the eight genes passing FDR < 1% that are neither DDG2P genes nor already reported in ref. ^20^, there are varying levels of prior evidence for their relevance to neurodevelopment (Supplementary Note 8).

Among the 12 non-DDG2P genes, we observed a significant enrichment of damaging de novo PTVs in a cohort of probands with neurodevelopmental conditions compared to null expectation^36^ (observed/expected = 2.59, *P* = 6.8 × 10^−4^, one-tailed Poisson test; Supplementary Note 8). Furthermore, we found that, in aggregate, PTVs in the newly discovered genes were significantly negatively associated with measured cognitive ability in two birth cohorts (ALSPAC, *n* = 5,283; Millennium Cohort Study (MCS), *n* = 5,621 (ref. ^37^); Fig. 3d). Thus, damaging variants in genes discovered after incorporating imputed FI are associated with direct measures of cognitive ability and with elevated risk of neurodevelopmental conditions.

**Extended Data Fig. 5:**
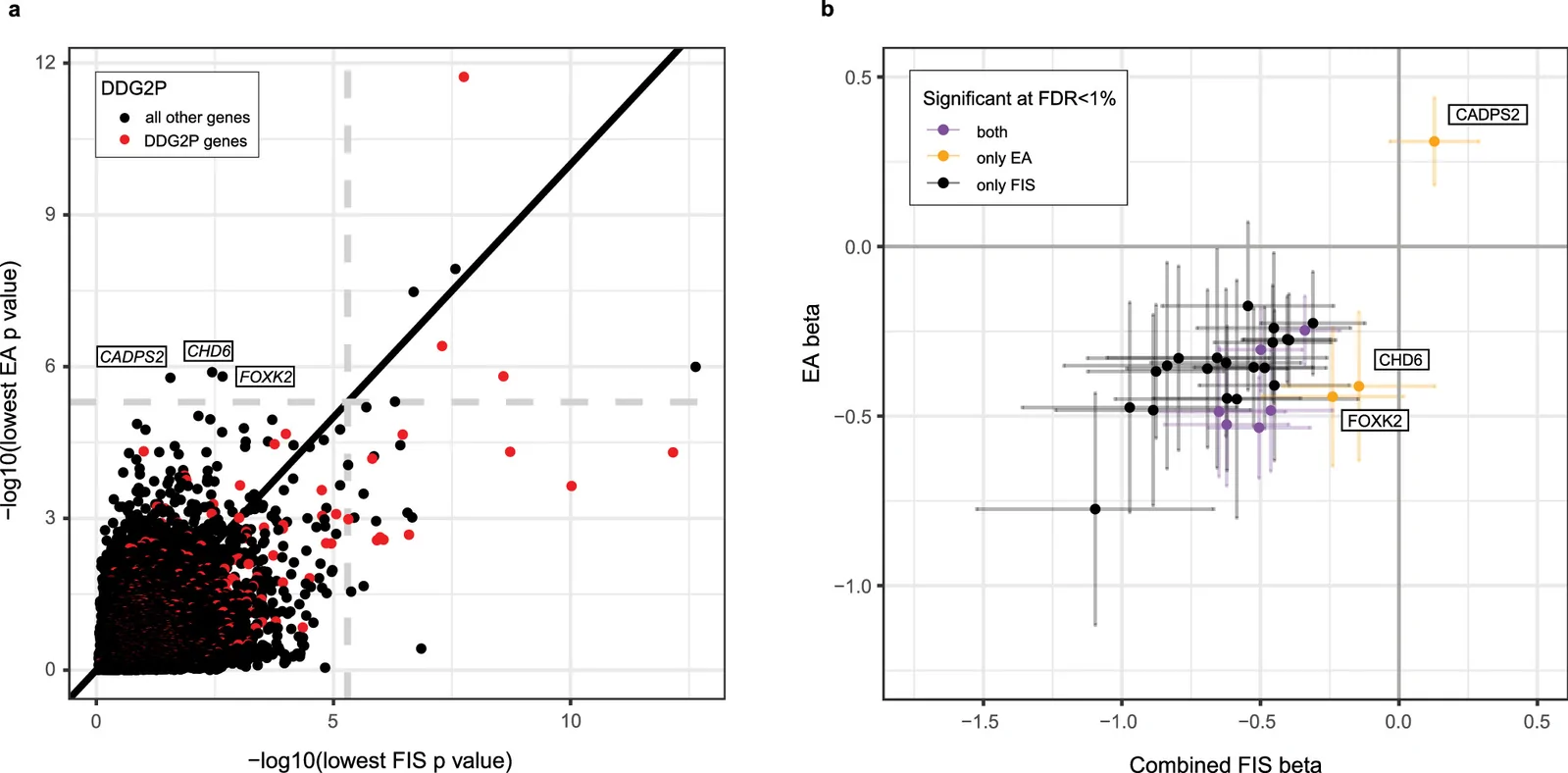
Results from gene-based rare variant burden and SKAT-O tests using FIS phenotypes versus educational attainment. **a**, On the *x*-axis is the −log_10_(*P*) for the lowest *P* value attained in any test for any of the FIS phenotypes, and on the *y*-axis is the −log_10_(*P*) for the lowest *P* value attained in any test for educational attainment (two-sided *t*-test for burden associations, nondirectional omnibus test for SKAT-O). Gray dashed lines indicate the empirical *P-*value cutoff for an FDR < 1% in our gene association tests (*P* < 5.03 × 10^−6^). **b**, On the *x*-axis is the beta for the pLoF burden test on combined FIS (*n* = 438,285), and on the *y*-axis is the pLoF burden test on EA (*n* = 434,146), with points colored based off whether the gene was significant at 1% FDR in either FIS only, EA only, or both. Error bars indicate 95% confidence intervals (estimate ± 1.96 × s.e.) for both beta estimates per gene (two-sided *t*-test).

To evaluate the advantage of using imputed FI over using EA, we compared rare variant discovery power between them (Extended Data Fig. 5 and Supplementary Note 9). We found only nine FDR-significant genes for EA compared to 26 for combined FIS. Six of these were overlapping, while three were specific to EA (*FOXK2*, *CHD6* and *CADPS2*), none of which were known neurodevelopmental condition-associated genes. This suggests that our imputation approach boosts power over what can be achieved with EA alone; however, some rare variants may influence EA through noncognitive mechanisms or by influencing other aspects of cognition not captured by the UKB FI test.

## Discussion

In this study, we evaluated and applied strategies for integration and imputation of FI phenotypes in UKB. We demonstrated that our approach yields a phenotype that is highly genetically similar to measured intelligence (Fig. 1 and Supplementary Fig. 3), while reducing ascertainment bias (Fig. 1f). Adding imputed FIS to observed FIS increased power across multiple analyses: the number of lead SNPs in the GWAS increased by 1.4-fold (Fig. 1d), MAGMA-prioritized genes by 1.2-fold (Fig. 2a), PGI prediction accuracy in both population-based and within-family analyses in external cohorts by 1.15-fold (Fig. 2c) and gene-level rare variant associations by 5.2-fold (Fig. 3c).

Our study highlights the importance of evaluating the properties of an imputed phenotype using both phenotypic and genetic evidence. Refining our predictor set (154 to 82 variables; Supplementary Note 3 and Supplementary Figs. 7 and 8) reduced the imputation accuracy modestly (*r* = 0.52 to *r* = 0.46) but simultaneously reduced the inflated genetic correlation with traits such as NonCogEA and height we observed in the initial imputation (Supplementary Fig. 4). This reinforces the notion that including more correlated phenotypes in the imputation can sometimes increase accuracy at the cost of specificity^27^. This accuracy of 0.46 was below the test–retest reliability (~0.6), although downstream analyses of the combined FIS GWAS preserved patterns of downstream genetic associations with other traits (for example, Fig. 1c). These observations suggest that the imputation introduced additional noise but not systematic bias of the kind that would distort LDSC estimates, consistent with the theory we develop in Supplementary Note 2. The modestly lower LDSC-SNP *h*^2^ observed for combined FIS than observed FIS (0.16 versus 0.20; Fig. 1) is likely due to this additional noise. Notably, we replicate both the common and rare variant signals obtained using the imputed values through a variety of analyses of direct measures of intelligence in external cohorts and showed that the incorporation of imputed individuals increases power (Figs. 2 and 3a).

**Extended Data Fig. 6:**
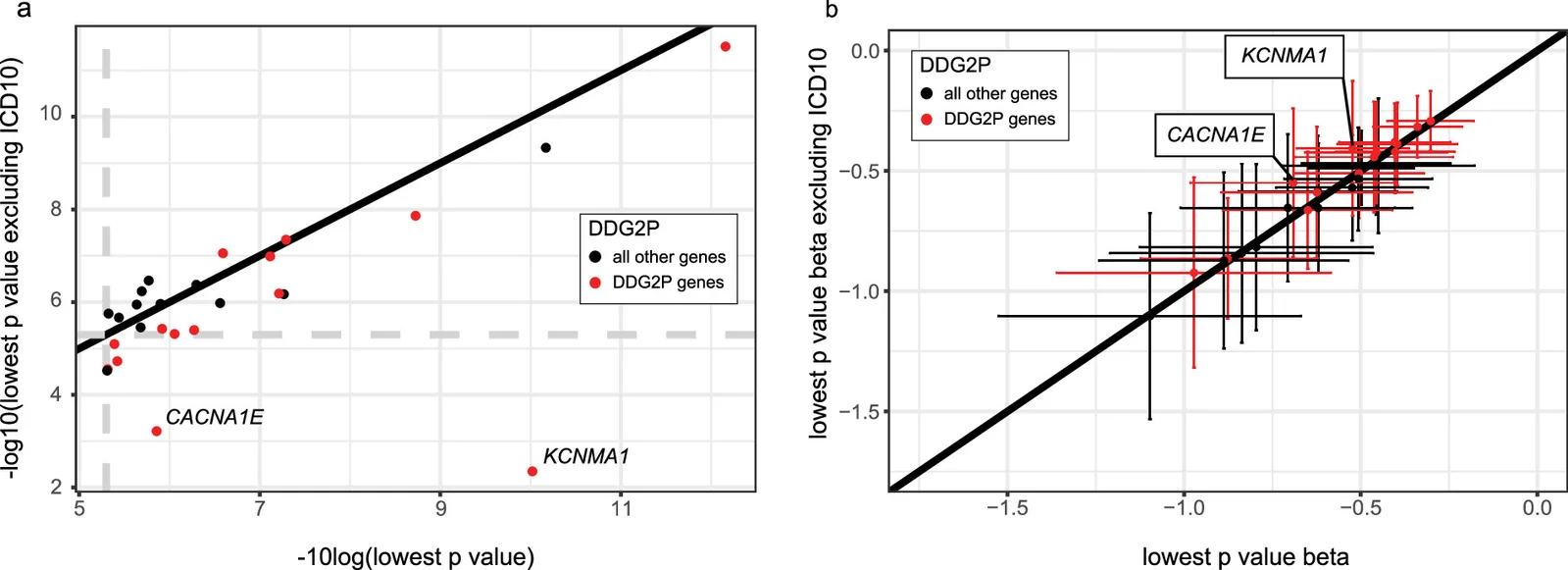
Results from gene-based rare variant burden tests before and after removing individuals with ICD-10 code indications for intellectual disability, autism, or epilepsy. **a**, On the *x*-axis is the −log_10_(*P*) for the most significant *P* value of a given gene among the 26 genes passing (FDR < 1%) across the 12 gene-based rare variant tests (two-sided *t*-test for burden associations, nondirectional omnibus test for SKAT-O). On the *y*-axis is the −log_10_(*P*) for the same test after removing individuals with ICD-10 codes indicating intellectual disability, autism, or epilepsy. **b**, On the *x*-axis is the beta from the gene burden test using the variant consequence class leading to the lowest *P* value per gene (two-sided *t*-test). On the *y*-axis is the beta from the same test after removing individuals with ICD-10 indicating intellectual disability, autism, or epilepsy. Solid black lines indicate the *y* = *x* line. Gray dashed lines indicate the empirical *P-*value cutoff for an FDR < 1% in our gene association tests (*P* < 5.03 × 10^−6^). Error bars indicate the 95% confidence intervals (estimate ± 1.96 × s.e.) for each beta.

We showed that incorporating imputed FIS into rare variant analyses enhanced power for gene discovery beyond what we could achieve using observed FI or EA alone (Supplementary Note 9), allowing us to expand the number of significant gene-level associations from 5 to 26 (Table 2), without changing effect size estimates (Fig. 3a,b). Fourteen of these significant genes are DDG2P genes and well-established causes of neurodevelopmental conditions. These associations were largely unchanged after excluding individuals with recorded neurodevelopmental condition diagnoses (Supplementary Note 8, Extended Data Fig. 6 and Supplementary Table 13), suggesting that rare variants in these genes can affect cognition without necessarily pushing individuals over the diagnostic threshold. Given our estimated effect sizes for rare variants in these genes on FI (~0.25–1 s.d.), we would not expect all carriers of such variants to have clinically recognized intellectual disability. It is likely that polygenic background^38^ and potentially environmental factors modify the penetrance of rare damaging variants in these genes for clinical phenotypes. Our rare variant findings expand our knowledge of the mechanism of action and mode of inheritance of several DD-associated genes and implicate several genes not previously known to have a role in human neurodevelopment (Supplementary Note 8). They also support the notion that there are more DD-associated genes than currently appreciated in which the ‘dominant/recessive’ dichotomy does not accurately reflect a gradient of heterozygous effects^39^.

This work has several limitations. First, because the imputation necessarily leverages variables associated with both cognitive performance and socio-environmental factors, the resulting phenotype probably reflects a mixture of cognitive and socio-environmental influences, as with measured FI (and many other traits), albeit in different proportions. Because the precise ratio of cognitive to noncognitive signal cannot be fully resolved empirically, it remains unclear the extent to which elevated genetic correlations with NonCogEA reflect model misspecification in the construction of the NonCogEA GWAS (of forcing the NonCog and Cog components to have an *r*_g_ of 0) versus importation of truly noncognitive signal. Second, we cannot rule out that the associations between rare variants in the known DD-associated genes and FI are driven by people with neurodevelopmental condition diagnoses, as these may be incompletely ascertained in the health records. Third, although we showed that these genes replicate in aggregate in ALSPAC and MCS, we have not attempted replication of individual genes because no cohort exists with sufficient power.

Finally, and more broadly, these results reflect a pragmatic response to limitations of existing biobank data rather than an endorsement of prediction/imputation as a substitute for measurement. While statistical imputation can improve power and reduce selection bias, it necessarily blurs the boundary between the construct of interest and its correlates, and imputed phenotypes should therefore not be treated as interchangeable with direct cognitive measurements. Notably, many of the conclusions depend on two assumptions that cannot be proven to hold definitively, namely, that classical measurement error in the FI test is uncorrelated with genotype and that the imputed phenotype is sufficiently calibrated to the intended FI construct in the unmeasured group, both in the conditional mean and in its covariance with each SNP (Supplementary Note 2). Thus, researchers considering this approach in other settings should weigh the power gains against the plausibility of these calibration assumptions in their specific application and should verify them empirically wherever possible by checking that external genetic-correlation profiles are preserved. Ultimately, the most robust path forward for genetic studies of cognition is improved phenotyping—the routine collection of high-quality, theory-driven cognitive measures alongside strategies to minimize selection and ascertainment bias at the design stage, rather than relying on post hoc statistical correction.

In summary, we have demonstrated how integrating and imputing FI measures in UKB improves power for genetic discovery while reducing ascertainment bias. Our findings reinforce the idea that rare variants with moderate effects on cognition are more pervasive than previously appreciated and that integrating carefully imputed phenotypes can provide a powerful avenue for identifying new contributors to neurodevelopmental processes, even among individuals without diagnosed conditions. Future studies should seek to integrate these findings with those from clinical cohorts to increase power to find new genes associated with neurodevelopmental conditions. Furthermore, studies in populations of non-European ancestries will be critical to explore the generalizability of these findings. Finally, this work motivates the broader use of phenotype imputation for genetic studies in large biobanks and contributes a formal framework deriving principled criteria for evaluating imputed phenotypic values (Supplementary Note 2).

## Methods

### Ethics oversight

UK Biobank obtained ethical approval from the NHS North West Centre for Research Ethics Committee (reference 11/NW/0382) and approved the use of data for this study.

### Sample selection

We conducted all analyses only in individuals who genetically cluster with European ancestry (*n* = 455,943). This was inferred by first projecting principal components (PCs) from 1000 Genomes onto the UKB participants and excluding participants who did not cluster with the European populations from the 1000 Genomes Project. After this, another principal component analysis was conducted to capture ancestry differences within the genetically more homogeneous individuals with European/British ancestries^72^.

### FIS score transformations

To correct for age decline in FI over time and differences in scales across FI tests, we transformed the FIS measures. For each FIS separately, we ran a regression model: FIS ~ age + age^2^, extracted the residuals and added the intercept to allow for differences in means between measures. The age used was the participant’s age at the time of the respective measurement, which was either obtained from UKB variable ‘age attended assessment center’ (Data-Field 21003) or approximated from ‘when FI test completed’ (Data-Field 20135) using the participants’ birth year and month.

For the online measures (FIS4 and FIS5), we did an additional transformation, setting all scores of 14 to 13, before running the regression model. This broadly aligns the scales of the online measures (14 questions) with those of the in-person test (13 questions; Supplementary Note 1).

### Imputation

#### SoftImpute

The R package SoftImpute^73^ was used to impute FIS. SoftImpute is a matrix completion algorithm that approximates missing values by identifying and leveraging patterns in the available data. It does so by minimizing an objective function consisting of two terms—(1) the distance between the observed entries in the observed and imputed data matrices according to the Frobenius norm, and (2) the product of a tuning parameter *λ* and the sum of the singular values of the imputed data matrix. Intuitively, this means the algorithm fills in missing values in a way that minimizes the rank of the imputed data matrix, favoring low-dimensional structure over complexity. It starts with an initial guess for the missing values, then iteratively refines this guess by applying a soft-thresholded singular value decomposition on the complete matrix. The main parameters specified in SoftImpute are rank and *λ*. Rank determines the maximum number of factors the method uses to represent the data, and should always be set to at most *N*_variables_ − 1. *λ* controls the amount of smoothing applied during imputation. A high *λ* aims at lower model complexity, making it less sensitive to noise and more prone to overlook nuances. Ideally, *λ* is set to be slightly less than the rank set.

#### Variable selection

Our imputation strategy underwent several rounds of refinement (Supplementary Note 3), one of which concerned the selection of imputation variables. Here we describe the criteria used for the initial selection and the steps taken to refine this set.

##### Initial selection

We first selected 152 UKB phenotypes among those collected at the initial assessment center visit that showed at least nominally significant correlation (*P* < 0.05) with observed FI, focusing on FIS1 as it had the largest sample available at the time. For continuous phenotypes, we chose those with an absolute phenotype correlation (|*r*_pheno_|) with FIS1 >0.05 at a significance threshold of *P* < 0.05. Categorical phenotypes were first classified into ordinal and nominal types and then further evaluated. For ordinal phenotypes, we verified whether the values could be interpreted as a quantitative variable, reordered them if necessary and then retained those with |*r*_pheno_| > 0.05. We converted nominal phenotypes with *n* categories into *n* − 1 binary variables, after which we calculated the *r*_pheno_ between each of those binary variables and FIS1. We only included variables with two or more categories having |*r*_pheno_| > 0.1. We excluded phenotypes that are components of any FIS measure. In total, we selected 90 continuous phenotypes, 59 ordinal phenotypes and 3 nominal phenotypes (Supplementary Table 4). For the three nominal phenotypes, we selected the categories showing a strong correlation with FIS1 (|*r*_pheno_| > 0.1), coded them as additional binary phenotypes and removed the original phenotypes, ultimately leading to a set of 154 variables used as our initial set. Finally, we coded all missing values (for example, ‘preferred not to answer’ or ‘unknown’) as NA. Based on the 154 resulting phenotypes, we ran imputation with SoftImpute parameters—rank = 150, *λ* = 120.

#### Final selection

In our final selection, we narrowed the initial selection of variables down to phenotypes that correlate more specifically with cognitive signal. To examine which phenotypes fall within this criterion, we derived a GWAS of the ‘noncognitive component of imputed FIS’ (NonCog-iFIS) from our first FIS imputation. To do this, we applied a GenomicSEM model (Supplementary Fig. 7) for GWAS-by-subtraction as applied in ref. ^30^. GenomicSEM is an R package that allows fitting structural equation models on summary statistics from GWAS. In GWAS-by-subtraction models, a model is fitted that includes two phenotypes (GWAS) and two latent factors. The first latent factor represents the commonalities between phenotypes by regressing both phenotypes on this latent factor. The second latent factor comprises the genetic variance unique to one of the phenotypes. This is achieved by regressing the remaining genetic variance for the phenotypes on the latent factor after regressing out the variance captured in the first latent factor. Subsequently, both latent factors can be regressed on individual SNPs to obtain GWAS. In our analysis, we ran a GWAS-by-subtraction model using intelligence as mentioned in ref. ^14^, and in our first iteration, imputed FIS values as phenotypes. The model allows us to capture the genetic variance unique to this imputed FIS in the ‘NonCog’ latent factor and subsequently regress individual SNPs on this factor to obtain the NonCog-iFIS GWAS.

We then computed genetic correlations between each of the 154 imputation variables and intelligence in ref. ^14^ and the derived NonCog-iFIS GWAS (Supplementary Table 4). We retained phenotypes in the final selection if the absolute genetic correlation was stronger with intelligence than with NonCog-iFIS (Supplementary Fig. 8), resulting in 82 phenotypes being selected.

For imputations using the final selection of variables, we adjusted our SoftImpute parameters (rank = 80, *λ* = 70) due to the reduced number of selected phenotypes. We also explored the effects of different imputation parameters on imputation accuracy, but found that rank = 80 and *λ* = 70 achieved the highest imputation accuracy (Supplementary Fig. 9, left).

#### Removing outliers

After each imputation, we identified and removed outliers using the criterion:

$${X_{i}}\notin \left(\overline{X}-3\sigma ,\,\overline{X}+3\sigma \right)$$

where *X*_*i*_ represents an imputed score, $\overline{X}$ represents the mean of the measured score and *σ* represents the s.d. of the measured score.

In other words, we removed individuals whose imputed score was more than 3 s.d. from the mean of the observed scores. This results in slightly differing sample sizes across the imputation approaches (Supplementary Table 6).

#### Evaluating accuracy

To evaluate the imputation accuracy, we randomly selected 50,000 participants as the evaluation set. We introduced ‘synthetic missingness’ by setting FIS measures for these participants to be missing. After imputation, we examined the Pearson correlation between imputed FIS values and the original measures for our evaluation set and defined that correlation as the imputation accuracy. Only participants who have the targeted FI measure were included in calculation of accuracy for each FI measure (Supplementary Table 5).

We also examined whether the imputation was more accurate in different parts of the phenotype distribution. We classified observed values of the evaluation set into low, medium and high terciles, then examined the imputation accuracy within each tercile using the same strategy as above. We found the imputation accuracy to be higher in the low and high terciles than in the medium tercile (Supplementary Fig. 9, right).

### Combining measured and imputed FIS

Combining imputed and measured FIS was done by mega-analysis in all approaches. Before mega-analysis, imputed and measured values were scaled separately to have a mean of 0 and standard deviation of 1. While this may in principle bias associations, the resulting per-SNP bias scales with allele frequency differences between measured and imputed individuals, which are likely negligible (see Supplementary Note 2—Mega-analysis recovers population effects under calibrated imputation (Remark 10) for further discussion). We also evaluated combining imputed and measured values through meta-analysis (Supplementary Note 4 and Supplementary Table 6).

### GWAS

For the GWASs, we used REGENIE^34^ (v4.1) step 1 and step 2 on the UKB research analysis platform. For step 1, we used genotyped SNPs from UKB Data-Field 22418 passing standard quality control (missingness ≤ 0.1, MAF ≥ 0.01, Hardy–Weinberg equilibrium *P* ≥ 1 × 10^−15^). Individuals with genotype missingness > 0.1 were excluded.

In step 2, we analyzed imputed SNPs from UKB Data-Field 22828 that are in the Haplotype Reference Consortium^74^ and passing quality control in unrelated European individuals (missingness < 0.05, MAF > 0.001, Hardy–Weinberg equilibrium *P* > 1 × 10^−10^). To obtain the heteroskedasticity-robust standard error estimator in REGENIE, we specified a dummy interaction covariate and set ‘--rare-mac 0’ to force the robust estimator to be used for all SNPs. The dummy interaction covariate was generated to be a random standard normal variable.

The covariates (‘-*-*covarFile’) included in both steps were 25 genetic PCs that capture ancestry differences within European individuals^72^, age (at time of respective FIS measurement), age^2^, age × sex, age^2^ × sex and the array used to measure each individual’s genotype and sex as binary variables. For the average FIS approach, we computed the average age across measures and used that as the age covariate. For individuals with imputed average FIS, age was set to the UKB variable ‘age initial assessment visit’ (Data-Field 21003, Instance 0).

### Meta-analysis

For meta-analyses, we used METAL^75^ with the ‘STDERR’ approach. This weights effect size estimates by the inverse of corresponding standard errors. We only include SNPs with *n* > 10,000.

### Genetic correlations and SNP *h*^2^

LDSC^71^ was used to compute genetic correlations and SNP heritabilities. This tool requires munged (parsed) sumstats. In munging, we aligned SNPs with those in the HapMap 3 (ref. ^76^) set using the ‘--merge-alleles’ flag. Heritabilities and genetic correlations were computed using the ‘--h2’ and ‘--rg’ flag, respectively, with default parameters. To estimate the genome-wide correlation between the direct effects and NTCs, we used the SNIPAR package correlate.py script^33^. We applied a block-jackknife procedure to test whether estimates differed significantly across imputation approaches. To do this, we derived the intersection of SNPs included in each GWAS, ordered them by chromosome and then by position, and divided them into 200 blocks of ~5,077 SNPs.

### Identifying lead SNPs

The online platform for FUMA^32^ SNP2GENE was used to identify lead SNPs. First, independent significant SNPs are identified (*P* < 5 × 10^−8^, *r*^2^ < 0.6). Subsequently, identified significant SNPs are designated lead SNPs if they are independent from each other at a second threshold of *r*^2^ < 0.1.

### Gene prioritization and tissue expression

MAGMA^77^ (as implemented in FUMA’s SNP2GENE process) was used for gene prioritization and tissue expression analyses using the results from the population-based GWAS. MAGMA aggregates SNP-level data into gene-level data and performs a gene-based association test to identify significantly associated genes. The gene window was kept at 0 kb, restricting analysis to SNPs located within a gene. Resulting gene-based *P* values were downloaded and FDR corrected using the Benjamini–Hochberg procedure. To obtain our set of prioritized genes, we selected protein-coding genes with an FDR < 1% that were also MANE select transcripts^78^. We then applied the SNP2GENE process in FUMA to test whether the significant genes were enriched in particular tissues; this takes the gene-level *P* values from MAGMA as input. We used expression data from 54 tissues from GTEx (v8; ref. ^79^) as reference data.

### Within-family GWAS

We conducted within-family GWAS in UKB using the SNIPAR package^33^ in individuals with European ancestry. SNIPAR leverages the presence of genotyped first-degree relatives to impute missing parental genotypes, allowing for their downstream use to conduct within-family GWAS. We estimated pairwise kinship coefficients using KING^80^ and used default parameters to conduct the within-family GWAS using scripts provided in SNIPAR. We controlled for the same covariates as in the population GWAS. SNP heritability estimates were estimated using LDSC as described above. We filtered to SNPs with INFO score > 0.98 for all analyses using SNIPAR-imputed individuals, including the GWAS and PGI analyses.

### Whole-exome sequencing data in UKB and quality control

Whole-exome-sequencing data were generated at the Regeneron Genetics Center, and the sequencing procedure has been described in previous studies. We used custom applets to perform quality control for the whole-exome sequencing data of 469,836 participants within the UKB research analysis platform. First, we used BCFtools norm to split and left-align multi-allelic variants in the population-level Variant Call Format files into separate alleles. Next, we performed genotype-level filtering using BCFtools filter separately for single nucleotide variants (SNVs) and insertions/deletions. Specifically, SNV genotypes with a depth below 7 and genotype quality below 20, or insertions/deletion genotypes with a depth below 10 and genotype quality below 20, were set to missing. We also applied a binomial test to check for an expected alternate allele contribution of 50% for heterozygous SNVs, and SNV genotypes with a binomial test *P* ≤ 0.0001 were set to missing. Finally, we recalculated the proportion of missing genotypes for each variant and excluded all variants with more than 50% missingness.

Next, we annotated the variants using the ENSEMBL Variant Effect Predictor (VEP; v104) with the --everything flag. For each variant, we prioritized a single ENSEMBL transcript based on whether the transcript was protein coding, MANE Select (v0.97) or the VEP canonical transcript. The variant consequence was prioritized based on severity as defined by VEP. After annotation, we grouped stop-gained, frameshift, splice acceptor and splice donor variants into a single PTV category. Missense and synonymous variant consequences were defined according to VEP criteria, and only autosomal variants within ENSEMBL protein-coding transcripts were retained for further analysis.

We further filtered variants by MAF, retaining only those with MAF <0.001%. These variants were annotated with LOFTEE, REVEL, AlphaMissense, and MPC for further filtering. For PTVs, only high-confidence PTVs defined by LOFTEE were retained. For missense variants, a damaging missense variant set was created by including variants with AlphaMissense scores > 0.56, REVEL scores > 0.5 and MPC scores > 2.

### Exome-wide burden tests of rare coding variants

To examine the association between FIS and the burden of rare coding variants, we counted the number of rare PTVs, missense variants, damaging missense variants (as described above) and synonymous variants both in exome-wide and in high loss-of-function-intolerant (pLI > 0.9) genes. The variant burden was then used as the predictor variable in linear regression models, with FIS (observed, imputed and combined) as the response variables. The models were run in unrelated participants with European ancestry (*n* = 328,795). We controlled for the top 25 PCs, as well as age, sex, age^2^ and the interactions between age and sex, and age^2^ and sex^72^.

### Gene-based burden test of rare coding variants

We performed gene-based burden tests on observed and combined FIS (standardized to mean 0 and variance 1) and EA (number of years of education) using a two-step regression analysis in REGENIE. As REGENIE accounts for relatedness and population structure, the gene-based tests were performed in all participants with European ancestry (*n* = 438,285; please note that this is lower than the sample size used for common-variant analyses, as not all participants had exome data). In the first step, REGENIE fits a stacked block ridge regression to produce a leave-one-chromosome-out genetic prediction of the focal phenotype. The association test is then carried out in the second step by fitting regression models conditioned on the leave-one-chromosome-out predictions. For both steps, we adjusted for sex, age, age^2^, sex-by-age interaction, sex-by-age^2^ interaction, the top 25 PCs and recruitment centers (as categorical variables) to control for population structure and age. The FIS scores were rank based and inverse-normal transformed, as recommended by REGENIE. EA was coded in ref. ^81^ by using individual reports of highest attained qualification, with ‘college or university degree’ counting as 20 years, ‘other professional qualifications’ as 15, ‘A levels/AS levels or equivalent’ as 13, ‘O levels/GCSEs or equivalent’, ‘CSEs or equivalent’ as 10 and ‘none of the above’ as 7. We ran both burden tests and SKAT-O tests on three consequence classes—PTV, damaging missense and PTV + damaging missense. Thus, for FIS we conducted 12 tests per gene (including 2 phenotypes, 2 statistical methods, 3 consequence classes). To account for multiple testing, we calculated the FDR using the Benjamini–Hochberg method across the vector of all *P* values and considered genes passing FDR < 1% as ‘significant’. For EA, we conducted six tests per gene as we only considered a single phenotype, and similarly accounted for multiple testing by calculating the FDR across a vector of all *P* values and considering genes passing FDR < 1% as ‘significant’. We used the DDG2P gene list downloaded on 5 February 2025 for annotating genes as ‘well-established’ developmental condition genes.

### Replication analyses in external cohorts

Information about the replication cohorts (ALSPAC, MCS and INTERVAL) is given in Supplementary Methods. Quality control and imputation of genotype data in ALSPAC and MCS were conducted in ref. ^82^ and are summarized in Supplementary Methods, as is the preparation of the exome data, which was described in ref. ^37^. Quality control in INTERVAL was conducted in refs. ^83,84^ and is summarized in Supplementary Methods.

#### Cognitive performance measures

In ALSPAC, we considered IQ measured at age 8 using the Wechsler Intelligence Scale for Children test^85^. We included 5,283 unrelated children with genetically inferred European ancestry and at least one genotyped parent in our analyses. In MCS, we derived a cognitive performance measure as previously described in ref. ^82^ by fitting a one-factor model and calculating scores using ‘factanal’ and Bartlett scoring in R using the following measures: Bracken School Readiness at age 3 years, reading vocabulary at ages 3 and 5 years, pattern construction at ages 5 and 7 years, and word reading and progress in math at age 7 years. The summarized measure explained 39% of the variance, and we included a total of 5,621 unrelated children of genetically inferred European ancestry with at least one genotyped parent. In INTERVAL, participants completed an FI test identical in nature to the one completed by UKB participants in the first in-person wave at two time points 12 months apart (test–retest correlation = 0.65, *P* < 10^−15^). We took the average of the FIS for individuals who had two measurements. We included 20,328 unrelated individuals of genetically inferred European ancestry. All variables were standardized to have a mean of 0 and variance of 1.

#### Calculating PGIs

We used LDpred2-auto^86^ to calculate PGIs in ALSPAC, MCS and INTERVAL using the GWAS for observed FIS (that is, measured average FIS), combined FIS and combined FIS + COGENT GWAS meta-analysis. We also used unrelated individuals with European ancestries (Supplementary Methods) and generated LD reference panels restricted to HapMap 3 + SNPs^86^, which are designed to maximize genome-wide tagging coverage, improving performance across diverse ancestries compared to the original set. We used default parameters to calculate SNP weights for the PGIs. We then imputed missing parental genotypes in both cohorts (that is, if one but not both parents were genotyped) using SNIPAR^33^ as previously described^82^ and calculated PGIs with the SNP weights generated above using the pgs.py script. We filtered PGI SNPs to those with INFO score > 0.98 in each given cohort.

#### Estimating direct and population effects of PGIs on cognitive performance measures

To estimate the population effects of the PGIs, we standardized the PGIs as above and regressed the phenotypes on the individual’s PGI, 20 genetic PCs and sex, and additionally age and age^2^ in INTERVAL, using the ‘lm’ function in R. To estimate the direct effects in ALSPAC and MCS, we added the two parental PGIs and additional covariates to the previous regression. The coefficient estimated for the child’s PGI is the partial correlation between the PGI and the phenotype, and represents the direct genetic effect.

In MCS, we accounted for ascertainment biases due to the cohort’s nonrandom sampling scheme and attrition by incorporating sampling weights as previously described^8^. Briefly, we generated nonresponse weights using inverse probability weighting and multiplied these weights with the full UK sampling weights generated by the study. We then used these weights in the regression analyses conducted in MCS using the weights argument in the ‘lm’ function in R.

#### Multilevel mixed-effects regression

We used a multilevel mixed-effects regression to evaluate the association between discovery GWAS sample size and standardized PGI effect sizes using the ‘metafor’ package in R^87^. We regressed the PGI *β*s, weighted by their inverse variance to account for measurement error, on the logarithm of the GWAS discovery sample size.

Random intercepts were included for the cohorts (ALSPAC, MCS and INTERVAL) and for effect type (population and direct) nested within each cohort to account for heterogeneity in effect estimates across these groups. The model was specified as:

$${\beta }_{\left(\mathrm{ijk}\right)}={\gamma }_{0}+{\gamma }_{1}\mathrm{log}\left({n}_{\left(\mathrm{ijk}\right)}\right)+{u}_{{\rm{i}}}+{v}_{{\rm{j}}}\left(i\right)+{\varepsilon }_{\left(\mathrm{ijk}\right)}$$

where *γ*_0_ is the overall intercept, *γ*_1_ represents the fixed effect of log(*n*), *u*ᵢ is the random effect for the *i*th cohort, *v*ⱼ(*i*) is the random effect for effect type nested within the *i*th cohort and *ε*₍ᵢⱼₖ₎ is the residual error. We also ran the model using direct effects estimates alone, dropping the *v*ⱼ(*i*) term.

#### Enrichment of de novo damaging variants in probands with neurodevelopmental conditions

We used previously published data from ref. ^21^ on three cohorts totaling over 31,000 exome-sequenced probands with developmental conditions and their parents to assess enrichment of damaging de novo mutations in the set of 12 genes with rare variant associations with FIS (FDR < 1%) that are not DDG2P genes.

We evaluated enrichment of de novo synonymous and nonsynonymous variants in the probands as follows. We used the null mutational model^36^ to compute, for each consequence class *i*, the cumulative per-site mutation rate *μ*_*i,g*_ across all callable variants of that class in a given gene *g*, that is, the summed mutation rate across all sites in a gene. The expected burden of de novo mutations of class *i* for a given gene-set *G* was then calculated as:

$${E}_{i,G}=\sum _{g\in G}{n}_{g}{\mu }_{i,g},$$

where *n*_*g*_ is the total number of probands. We compared the observed count *O*_*i*_ of de novo mutations in class *i* to *E*_*i*_ by performing a one-sided Poisson test with rate parameter *λ* = *E*_*i*_, calculating *P*(*X* *≥* *O*_*i*_|*X* *~* Pois(*λ*)) as the significance of any excess de novo mutations. This framework allows assessment of whether damaging protein-truncating or missense variants occur more often than expected by chance, with synonymous variants serving as an internal negative control.

#### Replication of gene-based burden test results in ALSPAC and MCS

To replicate the results of our gene-based tests in UKB, we performed gene-set burden tests in ALSPAC and MCS. For both cohorts, we included only variants with a MAF lower than 0.1% in participants with European ancestry and had a gnomAD (v3; ref. ^18^) allele frequency of < 3 × 10^−5^ (corresponding to allele count < 5). As in UKB, we only retain high-confidence PTVs defined by LOFTEE^18^ and missense variants with an MPC score > 2. We then calculated the burden of PTVs for the following three gene sets: (1) all 26 genes discovered in UKB at FDR < 1%; (2) the FDR < 1% genes excluding the eight already identified in ref. ^20^; and (3) the 21 FDR < 1% genes that we only discovered when using combined FIS. We used linear regression models to examine the association between cognitive measures and the gene-set burden of PTVs in unrelated children with European ancestry in both the Avon Longitudinal Study of Parents and Children (ALSPAC; *n* = 5,283) and the MCS (*n* = 5,621), controlling for sex and population structure with the top ten PCs.

### Reporting summary

Further information on research design is available in the Nature Portfolio Reporting Summary linked to this article.

## Online content

Any methods, additional references, Nature Portfolio reporting summaries, source data, extended data, supplementary information, acknowledgements, peer review information; details of author contributions and competing interests; and statements of data and code availability are available at https://doi.org/10.1038/s41588-026-02787-5.

## Supplementary information

Supplementary Information Supplementary Glossary, Notes 1–9, Figs. 1–13 and Methods.

Reporting Summary

Peer Review file

Supplementary Tables Supplementary Tables 1–14.

## Data Availability

UKB data are available to researchers upon application (https://www.ukbiobank.ac.uk/enable-your-research/apply-for-access). Summary statistics from population- and within-family-based GWAS of combined FIS, measured FIS, imputed FIS and the meta-analysis of combined FIS and COGENT can be found on Zenodo (https://doi.org/10.5281/zenodo.21167984)^28^. Full results of rare variant analyses, including gene-level association statistics, are available in the Supplementary Tables.

## Code availability

The imputed FI phenotype is not publicly available due to participant privacy and UKB’s data sharing policies but can be regenerated by approved researchers using the code provided on GitHub (https://github.com/dmvandenberg/fluid-intelligence-imputation) and Zenodo^88^.
