# Supplementary material for: Imputation of fluid intelligence scores reduces ascertainment bias and increases power for analyses of common and rare variants
Source: Nat Genet. 2026 Oct 5;58(11):2784–93. doi: 10.1038/s41588-026-02787-5 (PMC13754570; doi:10.1038/s41588-026-02787-5)
Supplement: Supplementary file 1 [file 41588_2026_2787_MOESM1_ESM.pdf]

nature genetics

Supplementary information https://doi.org/10.1038/s41588-026-02787-5

Imputation of fluid intelligence scores reduces ascertainment bias and increases power for analyses of common and rare variants

In the format provided by the authors and unedited

Index

Index 1
Supplementary Glossary 3
Supplementary Box: Glossary of Abbreviations and Terms 3
Supplementary Notes 4
Supplementary Note 1: Available Fluid Intelligence data in UKB 4
Supplementary Note 2: Theoretical Framework for Interpreting Genetic Analyses of Observed and Imputed Phenotypes Subject to Measurement Error and Ascertainment Bias 5
Supplementary Note 3: Comparison of imputation strategies 22
Initial round of imputation focusing on the baseline FIS measure 22
Refining imputation variables to attempt to reduce “non-cognitive” signal 22
Using additional FIS measures to further reduce “non-cognitive” signal 23
Supplementary Note 4: Combining measured and imputed data 24
Supplementary Note 5: Performance of the All 5 imputed FIS strategy in different subsets 25
Supplementary Note 6: Power gains from increasing GWAS sample size 25
Supplementary Note 7: Rare variant analyses 26
Exome-wide burden analyses 26
Sensitivity analyses controlling for the number of singleton synonymous variants 27
Comparison of our results with those of Chen et al. 27
Supplementary Note 8: Rare variant associations with FIS 27
Characterization of the FIS-prioritized genes 27
Neurodevelopmental conditions association of twelve FIS-associated non-DDG2P genes 28
Rare variant gene associations in UKB after excluding individuals with neurodevelopmental conditions 29
Replication in birth cohorts 29
Supplementary Note 9: Rare variant associations with educational attainment 29
Imputed FIS versus educational attainment 29
Association between *CADPS2* and educational attainment 30
Supplementary Tables 31
Supplementary Table 1 31
Supplementary Table 2 31
Supplementary Table 3 32
Supplementary Table 4 32
Supplementary Table 5 32
Supplementary Table 6 32
Supplementary Table 7 32
Supplementary Table 8 32
Supplementary Table 9 32
Supplementary Table 10 33
Supplementary Table 11 33
Supplementary Table 12 33

Supplementary Table 13 33
Supplementary Table 14 33
Supplementary Figures 34
Supplementary Figure 1 34
Supplementary Figure 2 35
Supplementary Figure 3 36
Supplementary Figure 4 37
Supplementary Figure 5 38
Supplementary Figure 6 39
Supplementary Figure 7 40
Supplementary Figure 8 41
Supplementary Figure 9 42
Supplementary Figure 10 43
Supplementary Figure 11 44
Supplementary Figure 12 45
Supplementary Figure 13 46
Supplementary Methods 47
Introduction to the replication cohorts 47
Quality control and imputation of ALSPAC and MCS genotype data 47
Quality control of ALSPAC and MCS whole-exome sequencing data 48
Quality control and imputation of INTERVAL genotype data 48
Calculating PGIs in INTERVAL 49
Attempting replication of the rare variant association in CADPS2 with EA in All of Us 49
References 50

Supplementary Glossary

Supplementary Box: Glossary of Abbreviations and Terms

Abbreviation	Main text abbreviation	Description
**FIS**		Fluid intelligence score
***Observed FIS1**	FIS1	UKB baseline measured FIS (20016-0.0)
***Earliest observed FIS**	Earliest FIS	Earliest measured FIS across 5 measured tests
***Average observed FIS**	Average FIS/Observed FIS	Average measured FIS across 5 measured tests
****imputed average FIS**	Imputed FIS	Imputed FIS only, from imputation using the average of available FIS measures
****imputed earliest FIS**		Imputed FIS only, from imputation using the earliest of available FIS measures
****imputed All5 FIS**		Imputed FIS only, from imputation using the all 5 available FIS measures
***MegaFIS-vA**		Mega analysis of FIS1 and Imputed FIS1 using 154 imputation variables
***MegaFIS-vB**		Mega analysis of Observed FIS1 and Imputed FIS1 using 82 variables
***MegaFIS-average**	Combined FIS	Mega analysis of Average observed FIS and imputed average FIS
***MegaFIS-earliest**		Mega analysis of Earliest observed FIS and imputed earliest FIS
***MegaFIS-all5**		Mega analysis of FIS1 and imputed All5 FIS
**COGENT**	COGENT	GWAS meta-analysis of intelligence across 24 cohorts in the COGENT consortium by Trampush et al.[1] No UKB participants included.
**Savage et al. Intelligence**	Savage et al. Intelligence	GWAS meta-analysis of intelligence in 14 cohorts, including UKB, by Savage et al.[2]
**NonCog-iFIS**		GWAS-by-subtraction derived GWAS of Intelligence (Savage et al.) minus the first iteration of imputed FIS

See *Supplementary Figure 4 and **Supplementary Table 5 for sample sizes

Supplementary Notes

Supplementary Note 1: Available Fluid Intelligence data in UKB

Currently, the UK Biobank has six measurements of Fluid Intelligence Scores (FIS) (see Supplementary Table 1 and Supplementary Figure 1). Four of these measurements were taken in person during different types of visits, and the other two were taken via an online questionnaire. The in-person and online measures are on different scales; in the in-person measurements, thirteen questions had to be answered within two minutes, whereas in the online version, fourteen questions had to be answered in the same amount of time. The order of questions was the same across in-person and online measures, hence the fourteenth measure in the online questionnaire is additional. Only nine people attempted this additional question and answered it correctly within the two-minute time constraint. Thus, we decided to set FI scores of fourteen to thirteen to broadly align scales between measures, retaining these people in the top performing percentile.

We calculated the test-retest correlations among individuals with multiple FIS measurements (Supplementary Table 2). The test-retest correlations were fairly modest across all FIS measurements, in the order of r=~0.6, as previously reported[3]; thus, they are less reliable than standard IQ tests which have test-retest correlations closer to 0.9[4]. To verify whether the different FIS measurements capture similar genetic signals, we ran GWASs for each measure and computed genetic correlations between them (Supplementary Table 2). None of them had a genetic correlation with any other that was significantly lower than 1, except FIS5 which has a genetic correlation with FIS1 and FIS3 slightly below 1.

We noted that the mean and variance were largely consistent across FIS measures, with the exception of FIS5 having a slightly lower mean (Supplementary Figure 2A). This slightly lower mean is likely to be due to several factors, including participants experiencing cognitive decline as time went on as well as ascertainment biases in the characteristics of people taking the different tests. Notably, we observed that having completed the various fluid intelligence tests was associated with higher educational attainment, particularly for FIS2 to FIS5 (Supplementary Figure 2B). It has been shown that non-random ascertainment can influence heritability and genetic correlation estimates. Thus, we decided to impute fluid intelligence scores for UK Biobank participants for whom they were not measured, with two goals in mind: i) to increase power for genetic studies of fluid intelligence, and ii) to ameliorate the ascertainment bias whereby more educated individuals were more likely to complete the tests.

Supplementary Note 2: Theoretical Framework for Interpreting Genetic Analyses of Observed and Imputed Phenotypes Subject to Measurement Error and Ascertainment Bias

1 Introduction and Overview

This note develops a theoretical framework for genome-wide association analyses when the phenotype may be observed with error (measured scores with varying precision) or imputed from auxiliary variables. We formalize the genetic model and the respective roles of measurement error and imputation error, and prove identities linking imputation accuracy to correlation with the latent trait. We analyze phenotype-dependent selection into "measured" status and its implications (collider bias) under selection as a monotone increasing function of Y. We provide a proof that pooled ("mega-analysis") analysis of measured and imputed phenotypes consistently recovers population effects under phenotype-dependent selection, given the stated assumptions. We explain why errors are intrinsically heteroskedastic in the mega-analysis and why heteroskedasticity-consistent (sandwich) standard errors yield valid inference. Finally, we derive implications for LDSC single-trait SNP heritability and LDSC cross-trait genetic correlations, showing what should be observed when imputation adds noise without altering SNP-phenotype covariances. This yields a principled validation criterion: the mega-analysis r_g profile with external traits should match that from an independent intelligence GWAS.

Throughout, all random quantities are defined on a common probability space. Expectations, variances and covariances are population quantities; sample statistics converge in probability to their population analogues under standard regularity conditions. We write plim for probability limits as $n \to \infty$.

2 Basic Model and Notation

2.1 Genetic architecture and the latent trait

Let Y denote the (latent) continuous phenotype of interest for an individual, with an additive decomposition

$$Y = G + E, \tag{1}$$

where G is the additive genetic component tagged by the available genotypes and E includes non-additive genetic, environmental, and untagged effects. We model

$$G = \sum_{p=1}^{P} \beta_p X_p, \tag{2}$$

with $X_p \in \{0, 1, 2\}$ the coded count of a reference allele at SNP p and β_p its (population) additive effect on Y. Under Hardy-Weinberg equilibrium with allele frequency f_p, $\mathbb{E}[X_p] = 2f_p$ and

$\mathrm{Var}(X_p) = 2f_p(1 - f_p)$. We assume $\mathbb{E}[E] = 0$ and $\mathrm{Var}(E) = \sigma_E^2$, $\mathrm{Cov}(X_p, E) = 0$ for all p after residualizing for covariates, and

$$\mathrm{Var}(G) = \sum_{p,p'} \beta_p \beta_{p'} \,\mathrm{Cov}(X_p, X_{p'}) = \sigma_G^2,$$

so that $\mathrm{Var}(Y) = \sigma_G^2 + \sigma_E^2 = \sigma_Y^2$.

2.2 Measurement error versus imputation error

We distinguish *instrumental measurement error* from *imputation error*. Let $S \in \{0,1\}$ indicate whether a measured phenotype is available (S=1 measured; S=0 otherwise).

Definition 1 (Measured phenotype)**.** *A measured score is*

$$Y^m \;=\; Y + \epsilon^m \;=\; G + E + \epsilon^m.$$

Definition 2 (Imputed phenotype and calibration error)**.** *Let* $\mathbf{W}$ *be auxiliary variables used to impute the phenotype for individuals with* $S = 0$*. Let* $\hat{Y}$ *denote the imputed phenotype, and define the calibration error*

$$\delta := \hat{Y} - \mathbb{E}[Y \mid \mathbf{W}, S = 0].$$

Equivalently,

$$\hat{Y} = \mathbb{E}[Y \mid \mathbf{W}, S = 0] + \delta.$$

The term δ *measures the discrepancy between the imputed phenotype and the conditional mean of the target phenotype given the auxiliary variables in the unmeasured group.*

Assumption 1 (Classical measurement error)**.** *The instrument error satisfies*

$$\mathbb{E}[\epsilon^m \mid Y] = 0, \qquad \mathrm{Cov}(\epsilon^m, X_p) = 0 \;\; \forall p, \qquad \mathrm{Cov}(\epsilon^m, \hat{Y}) = 0.$$

For results that condition on measured status, including Theorem 5, we additionally assume

$$\mathbb{E}[\epsilon^m \mid S = 1] = 0, \qquad \mathrm{Cov}(\epsilon^m, X_p \mid S = 1) = 0 \quad \textit{for every } p.$$

We assume

$$0 < \mathrm{Var}(\epsilon^m) = \sigma_\epsilon^2 < \infty$$

within a given measurement instrument.

Assumption 2 (Mean and SNP-covariance preservation in the imputed phenotype)**.** *The imputed phenotype is calibrated in the unmeasured group in the following two population-moment senses. First,*

$$\mathbb{E}[\hat{Y} \mid S = 0] = \mathbb{E}[Y \mid S = 0].$$

Second, for each analyzed SNP p*,*

$$\mathrm{Cov}(X_p, \hat{Y} \mid S = 0) = \mathrm{Cov}(X_p, Y \mid S = 0).$$

Remark 1 (Interpretation)**.** *Assumption 2 is violated when the imputation changes the mean or SNP-covariance structure of the latent target in the unmeasured group, for example by importing genetically correlated non-target signal. Importing purely mean-zero, genotype-uncorrelated noise need not violate Assumption 2, although it can reduce precision and observed-scale SNP heritability. Reproduction of instrument-specific noise can also violate the orthogonality condition used in Theorem 1. This is why, in the empirical analysis, we treat downstream genetic checks as essential complements to phenotypic imputation accuracy.*

Assumptions 1 and 2 are the core assumptions for the main empirical use of the note. Assumption 1 underlies the measurement and reliability results and the interpretation of holdout correlations with measured FIS. Assumption 2 is the key assumption for the pooled GWAS of observed and imputed phenotypes, for the preference for mega-analysis over IVW meta-analysis, and for the LDSC validation criterion based on preserving the external r_g profile. The selection assumptions introduced in Section 6 are used only to derive interpretable within-group collider formulas and to motivate the direction of the direct-versus-NTC patterns. They are not needed for the mega-analysis consistency theorem or the LDSC invariance result.

3 Estimation of Genetic Effects in Population GWAS

3.1 Population regression with the latent Y

Define the (population) marginal slope for SNP p by

$$\beta_p^{\text{marg}} = \frac{\operatorname{Cov}(Y, X_p)}{\operatorname{Var}(X_p)}. \tag{3}$$

Using $Y = G + E = \sum_{p'} \beta_{p'} X_{p'} + E$,

$$\begin{aligned} \operatorname{Cov}(Y, X_p) &= \sum_{p'} \beta_{p'} \operatorname{Cov}(X_{p'}, X_p) + \underbrace{\operatorname{Cov}(E, X_p)}_{=0} \\ &= \beta_p \operatorname{Var}(X_p) + \sum_{p' \neq p} \beta_{p'} \operatorname{Cov}(X_{p'}, X_p). \end{aligned} \tag{4}$$

Thus

$$\beta_p^{\text{marg}} = \beta_p + \sum_{p' \neq p} \beta_{p'} \frac{\operatorname{Cov}(X_{p'}, X_p)}{\operatorname{Var}(X_p)}, \tag{5}$$

which equals β_p in the absence of LD ($\operatorname{Cov}(X_{p'}, X_p) = 0$ for $p' \neq p$).

3.2 Using a measured score Y^m

Under Assumption 1, with $Y^m = Y + \epsilon^m$ and classical measurement error (Definition 1),

$$\beta_p^{\text{marg,meas}} = \frac{\operatorname{Cov}(Y^m, X_p)}{\operatorname{Var}(X_p)} = \frac{\operatorname{Cov}(Y, X_p) + \operatorname{Cov}(\epsilon^m, X_p)}{\operatorname{Var}(X_p)} = \beta_p^{\text{marg}}, \tag{6}$$

so instrumental noise does not change the (population) coefficient; under the stated conditions, it adds σ_ϵ^2 to the residual variance that would obtain using the latent phenotype and therefore reduces precision. In practice, if the measured phenotype Y^m is standardized to have mean 0 and variance 1 before analysis, the population effect rescales to $\beta_p^{\text{marg,meas(std)}} = \beta_p^{\text{marg}} / \sigma_{Y^m}$, where $\sigma_{Y^m} = \sqrt{\sigma_Y^2 + \sigma_\epsilon^2}$. Sample OLS estimates $\hat{\beta}_p$ consistently estimate their corresponding population quantities.

4 Classical Test Theory and Reliability

Test-retest reliability connects measured scores to the latent trait. As shown by Propositions 1 and 2 below, when independent measurements have equal error variance σ_ϵ^2, the reliability $\rho =$

$\sigma_Y^2/(\sigma_Y^2+\sigma_\epsilon^2)$ determines the correlation between repeated measurements. When individuals have different numbers of replicate measurements k_i, the resulting heteroskedastic reliabilities $\rho_{k_i} = k_i\rho/(1+(k_i-1)\rho)$ lead to inherently heteroskedastic errors in the pooled analysis, necessitating robust inference procedures (Section 8).

4.1 Classical Test Theory Results

Proposition 1 (Test-retest reliability equals true-score fraction)**.** *Under Assumption 1, if* $Y_1^m = Y + \epsilon_1^m$ *and* $Y_2^m = Y + \epsilon_2^m$ *with independent replicate errors and* $\mathrm{Var}(\epsilon_j^m) = \sigma_\epsilon^2$*, then*

$$\mathrm{Cor}(Y_1^m, Y_2^m) \;=\; \rho \;=\; \frac{\sigma_Y^2}{\sigma_Y^2+\sigma_\epsilon^2}.$$

Proof. $\mathrm{Cov}(Y_1^m, Y_2^m) = \mathrm{Var}(Y) = \sigma_Y^2$ and $\mathrm{Var}(Y_j^m) = \sigma_Y^2+\sigma_\epsilon^2$, so the correlation is $\sigma_Y^2/(\sigma_Y^2+\sigma_\epsilon^2)$. □

From ρ, one recovers the error variance $\sigma_\epsilon^2 = \sigma_Y^2(1-\rho)/\rho$ and $\mathrm{Cor}(Y^m, Y) = \sqrt{\rho}$.

Proposition 2 (Spearman-Brown)**.** *Under Assumption 1, for* $\bar{Y}_k^m = k^{-1}\sum_{i=1}^k Y_i^m$ *with independent replicate errors, the reliability is*

$$\rho_k \;=\; \frac{k\rho}{1+(k-1)\rho}.$$

Proof. $\bar{Y}_k^m = Y + \bar{\epsilon}_k$, $\mathrm{Var}(\bar{\epsilon}_k) = \sigma_\epsilon^2/k$, and reliability is $\sigma_Y^2/(\sigma_Y^2+\sigma_\epsilon^2/k)$. Substitute $\sigma_\epsilon^2/\sigma_Y^2 = (1-\rho)/\rho$. □

5 Imputation Theory

5.1 Linking imputation accuracy to latent correlation

Let $r_{\text{imp}} = \mathrm{Cor}(\hat{Y}, Y^m)$ denote the population correlation between imputed and measured values in a masked holdout set. Let ρ denote the instrument test-retest reliability from Proposition 1.

Theorem 1 (Imputation accuracy and latent correlation)**.** *Under Assumption 1,*

$$\mathrm{Cor}(\hat{Y}, Y) \;=\; \frac{r_{imp}}{\sqrt{\rho}}.$$

Proof. With $Y^m = Y + \epsilon^m$ and $\mathrm{Cov}(\hat{Y}, \epsilon^m) = 0$,

$$\mathrm{Cor}(\hat{Y}, Y^m) = \mathrm{Cor}(\hat{Y}, Y)\sqrt{\frac{\mathrm{Var}(Y)}{\mathrm{Var}(Y)+\sigma_\epsilon^2}} = \mathrm{Cor}(\hat{Y}, Y)\sqrt{\rho}.$$

Rearrange. □

Remark 2 (Connection to empirical analysis)**.** *In the empirical application,* ρ *is estimated from repeated measured FI tests (Supplementary Table 2 of the paper) and* r_{imp} *from masked-holdout prediction experiments (Supplementary Table 5). The theorem explains how those two quantities jointly calibrate the latent-trait correlation being targeted by the imputation.*

6 Phenotype-Dependent Selection and Its Consequences

6.1 Selection mechanism

Let $S \in \{0, 1\}$ again denote whether a measured phenotype is available.

Definition 3 (Phenotype-dependent selection)**.** *Define*

$$\Pr(S = 1 \mid Y, \mathbf{C}) \;=\; h(Y, \mathbf{C}), \tag{7}$$

where $\mathbf{C}$ *collects other contributors to selection. We call selection phenotype-dependent if, for some fixed* $\mathbf{c}$*, the function* $h(\cdot, \mathbf{c})$ *is non-constant. We call the selection* monotone in Y *if, for each fixed* $\mathbf{c}$*, the function* $h(\cdot, \mathbf{c})$ *is non-decreasing.*

The explicit sign results below use the monotone case because it gives a directional interpretation of the collider term. The mega-analysis consistency result in Section 7 does not require monotonicity.

Assumption 3 (Selection moment invariance given Y and $\mathbf{C}$)**.** *Conditioning on* $S{=}1$ *does not change the conditional expectations of* G *and* E*, or their conditional covariance, once* $(Y, \mathbf{C})$ *are fixed. Specifically,*

$$\mathbb{E}[G \mid Y, \mathbf{C}, S{=}1] = \mathbb{E}[G \mid Y, \mathbf{C}], \qquad \mathbb{E}[E \mid Y, \mathbf{C}, S{=}1] = \mathbb{E}[E \mid Y, \mathbf{C}],$$

and

$$\mathrm{Cov}(G, E \mid Y, \mathbf{C}, S{=}1) = \mathrm{Cov}(G, E \mid Y, \mathbf{C}).$$

Remark 3 (Relation to stronger exclusion assumptions)**.** *A stronger but sufficient condition is that the full conditional distribution of* (G, E) *given* $(Y, \mathbf{C}, S{=}1)$ *equals that given* $(Y, \mathbf{C})$*. The results below need only the conditional means and conditional covariance stated in Assumption 3.*

Assumption 4 (Linear conditional projections)**.** *There exist constants* a_G, a_E *and vectors* γ_G, γ_E *such that*

$$\mathbb{E}[G \mid Y, \mathbf{C}] = a_G Y + \gamma_G^\top \mathbf{C}, \qquad \mathbb{E}[E \mid Y, \mathbf{C}] = a_E Y + \gamma_E^\top \mathbf{C}.$$

Remark 4 (A sufficient condition for Assumption 4)**.** *Joint Gaussianity of* $(G, E, Y, \mathbf{C})$ *is sufficient for Assumption 4, but the theorem below only needs linear conditional projections.*

Assumption 5 (Closed-form residual structure)**.** *The pair* (G, E) *is jointly Gaussian with means zero, variances* σ_G^2 *and* σ_E^2*, and* $\mathrm{Cov}(G, E) = 0$*. In addition, conditioning on* $\mathbf{C}$ *does not change the conditional expectations of* G *and* E*, or their conditional covariance, once* Y *is fixed:*

$$\mathbb{E}[G \mid Y, \mathbf{C}] = \mathbb{E}[G \mid Y], \qquad \mathbb{E}[E \mid Y, \mathbf{C}] = \mathbb{E}[E \mid Y],$$

and

$$\mathrm{Cov}(G, E \mid Y, \mathbf{C}) = \mathrm{Cov}(G, E \mid Y).$$

Remark 5 (Connection to empirical analysis)**.** *Assumptions 3, 4, and 5 are used only in this section, where the goal is to interpret the within-group collider patterns and, in particular, the direction of the direct-versus-NTC correlations. They are not used for the pooled combined-FIS GWAS, for the robust-standard-error argument, or for the LDSC validation criterion.*

6.2 Collider bias under selection as a monotone increasing function of Y

Theorem 2 (Decomposition of $\mathrm{Cov}(G, E \mid S{=}1)$). *Assume the relevant second moments exist. Then*

$$\mathrm{Cov}(G, E \mid S{=}1) \;= \mathbb{E}\big[\,\mathrm{Cov}(G, E \mid Y, \mathbf{C}, S{=}1)\,\big|\,S{=}1\big] \;+\mathrm{Cov}\big(\,\mathbb{E}[G \mid Y, \mathbf{C}, S{=}1],\, \mathbb{E}[E \mid Y, \mathbf{C}, S{=}1] \mid S{=}1\big). \tag{8}$$

If Assumption 3 holds, this simplifies to

$$\mathrm{Cov}(G, E \mid S{=}1) \;= \mathbb{E}\big[\,\mathrm{Cov}(G, E \mid Y, \mathbf{C})\,\big|\,S{=}1\big] \;+ \mathrm{Cov}\big(\,\mathbb{E}[G \mid Y, \mathbf{C}],\, \mathbb{E}[E \mid Y, \mathbf{C}] \mid S{=}1\big). \tag{9}$$

If Assumptions 3 and 4 also hold, then the same constants a_G, a_E and vectors γ_G, γ_E from Assumption 4 satisfy $a_G + a_E = 1$ and $\gamma_G + \gamma_E = \mathbf{0}$, and

$$\begin{aligned}\mathrm{Cov}\big(\,\mathbb{E}[G \mid Y, \mathbf{C}], \mathbb{E}[E \mid Y, \mathbf{C}] \mid S{=}1\big) = {} & a_G a_E \;\mathrm{Var}(Y \mid S{=}1) \\ & +\; (a_G \gamma_E^\top + a_E \gamma_G^\top)\;\mathrm{Cov}(Y, \mathbf{C} \mid S{=}1) \\ & +\; \gamma_G^\top \;\mathrm{Cov}(\mathbf{C} \mid S{=}1)\,\gamma_E. \end{aligned} \tag{10}$$

Proof. Equation (8) is the law of total covariance applied conditional on $(Y, \mathbf{C})$ within the selected group $S{=}1$. If Assumption 3 holds, the conditional means and conditional covariance in Equation (8) can be written without $S{=}1$, yielding Equation (9). Under Assumption 4,

$$\mathbb{E}[G \mid Y, \mathbf{C}] = a_G Y + \gamma_G^\top \mathbf{C}, \qquad \mathbb{E}[E \mid Y, \mathbf{C}] = a_E Y + \gamma_E^\top \mathbf{C}.$$

Since $Y = G + E$, taking conditional expectations given $(Y, \mathbf{C})$ gives

$$Y = (a_G + a_E)Y + (\gamma_G + \gamma_E)^\top \mathbf{C},$$

so $a_G + a_E = 1$ and $\gamma_G + \gamma_E = \mathbf{0}$. Taking the covariance of the two linear forms conditional on $S{=}1$ yields Equation (10). □

Corollary 3 (Special case giving a closed-form collider term). *Under Assumptions 3 and 5,*

$$\mathbb{E}\big[\,\mathrm{Cov}(G, E \mid Y, \mathbf{C})\,\big|\,S{=}1\big] \;=\; \mathrm{Cov}(G, E \mid Y) \;=\; -\frac{\sigma_G^2 \sigma_E^2}{\sigma_Y^2}.$$

Consequently,

$$\mathrm{Cov}(G, E \mid S = 1) \;=\; -\frac{\sigma_G^2 \sigma_E^2}{\sigma_Y^2} \;+ \frac{\sigma_G^2 \sigma_E^2}{\sigma_Y^4}\;\mathrm{Var}(Y \mid S = 1). \tag{11}$$

In particular, if $\mathrm{Var}(Y \mid S{=}1) < \sigma_Y^2$ *(variance contraction), then* $\mathrm{Cov}(G, E \mid S{=}1) < 0$*; if* $\mathrm{Var}(Y \mid S{=}1) > \sigma_Y^2$ *the sign flips.*

Proof. For a mean-zero bivariate Gaussian pair (G, E) with $Y = G + E$ and $\mathrm{Cov}(G, E) = 0$,

$$\mathbb{E}[G \mid Y] = \frac{\sigma_G^2}{\sigma_Y^2} Y, \qquad \mathbb{E}[E \mid Y] = \frac{\sigma_E^2}{\sigma_Y^2} Y,$$

and

$$\mathrm{Cov}(G, E \mid Y) = -\frac{\sigma_G^2 \sigma_E^2}{\sigma_Y^2}.$$

Assumption 5 states that conditioning further on $\mathbf{C}$ does not change these conditional expectations or the conditional covariance, so

$$\mathbb{E}[G \mid Y, \mathbf{C}] = \frac{\sigma_G^2}{\sigma_Y^2} Y, \qquad \mathbb{E}[E \mid Y, \mathbf{C}] = \frac{\sigma_E^2}{\sigma_Y^2} Y, \qquad \mathrm{Cov}(G, E \mid Y, \mathbf{C}) = -\frac{\sigma_G^2 \sigma_E^2}{\sigma_Y^2}.$$

Substituting these expressions into Equation (9) gives

$$\begin{aligned}
\mathrm{Cov}(G, E \mid S{=}1) &= \mathbb{E}[\mathrm{Cov}(G, E \mid Y, \mathbf{C}) \mid S{=}1] \\
&\quad + \mathrm{Cov}(\mathbb{E}[G \mid Y, \mathbf{C}], \mathbb{E}[E \mid Y, \mathbf{C}] \mid S{=}1) \\
&= -\frac{\sigma_G^2 \sigma_E^2}{\sigma_Y^2} + \mathrm{Cov}\left(\frac{\sigma_G^2}{\sigma_Y^2} Y, \frac{\sigma_E^2}{\sigma_Y^2} Y \mid S{=}1\right) \\
&= -\frac{\sigma_G^2 \sigma_E^2}{\sigma_Y^2} + \frac{\sigma_G^2 \sigma_E^2}{\sigma_Y^4} \mathrm{Var}(Y \mid S{=}1),
\end{aligned}$$

which is Equation (11). □

Remark 6 (Where the stronger closed-form assumptions matter)**.** *Assumption 5 is used only to obtain the closed-form expression in Corollary 3 and the associated sign intuition. Empirically, this part of the note is meant as a qualitative explanation for why the direct-versus-NTC correlation can become negative under selective measurement (Figure 1F in the main paper). These stronger distributional restrictions are not used in the mega-analysis or LDSC results later in the note.*

Remark 7 (Interpretation and working convention)**.** *Equation* (9) *shows that* any *selection component operating through* $\mathbf{C}$ *can generate additional collider paths via the three terms in* (10)*. For the remainder of the note, when we discuss the sign of the within-group collider term, we use the simplifying special case in Corollary 3 because it isolates the role of variation in* Y*. This special case may fail empirically if selection depends on ancestry- or environment-linked variables in* $\mathbf{C}$ *that remain informative about* G *or* E *after conditioning on* Y*.*

Remark 8 (When does $\mathrm{Var}(Y \mid S{=}1) < \sigma_Y^2$?)**.** *Variance contraction holds for many practically relevant monotone selection schemes from Definition 3 (e.g., one-sided thresholds* $S = \mathbb{1}\{Y > c\}$*). Note that the Gaussianity assumption is sufficient for the exact formula in Equation* (11)*.*

6.3 Bias in SNP effects estimated within $S = 1$ and $S = 0$

For expositional clarity, we assume that the LD-tagging component of the marginal association is absent, i.e.,

$$\mathrm{Cov}(X_{p'}, X_p) = 0 \text{ for } p' \neq p \quad \text{and} \quad \mathrm{Cov}(X_{p'}, X_p \mid S = s) = 0 \text{ for } p' \neq p, \; s \in \{0, 1\}.$$

In the Appendix we provide a full derivation with general LD, showing the exact form of the LD-tagging bias within $S = s$.

Theorem 4 (Average sign of within-group bias)**.** *Under the no-LD working simplification above, define for* $s \in \{0, 1\}$ *the within-group per-SNP* collider term

$$b_{p,s} \;\equiv\; \frac{\mathrm{Cov}(E, X_p \mid S = s)}{\mathrm{Var}(X_p \mid S = s)}.$$

Then

$$\sum_{p=1}^{P} \beta_p \operatorname{Var}(X_p \mid S = s)\, b_{p,s} \;=\; \operatorname{Cov}(E, G \mid S = s).$$

Consequently, under the special case of Corollary 3, if $\operatorname{Var}(Y \mid S{=}1) < \sigma_Y^2$ *(variance contraction), then*

$$\sum_{p=1}^{P} \beta_p \operatorname{Var}(X_p \mid S{=}1) \left(\beta_p^{\text{marg}|S=1} - \beta_p\right) \;<\; 0.$$

Proof. Within $S = s$,

$$\beta_p^{\text{marg}|S=s} = \frac{\operatorname{Cov}(Y, X_p \mid S = s)}{\operatorname{Var}(X_p \mid S = s)} = \beta_p \;+\; b_{p,s}.$$

Subtract β_p, multiply by $\beta_p \operatorname{Var}(X_p \mid S = s)$, and sum over p:

$$\sum_{p=1}^{P} \beta_p \operatorname{Var}(X_p \mid S = s) \left(\beta_p^{\text{marg}|S=s} - \beta_p\right) = \sum_{p=1}^{P} \beta_p \operatorname{Cov}(E, X_p \mid S = s) = \operatorname{Cov}(E, G \mid S = s).$$

Apply Corollary 3. □

Remark 9 (Connection to empirical analysis)**.** *The sign result is used only as a diagnostic interpretation, where a negative correlation between direct effects and non-transmitted coefficients is consistent with selective measurement. In the paper, movement of this correlation toward zero after integrating and imputing FIS is taken as evidence that ascertainment bias is being reduced, not as an identifying assumption for the pooled GWAS.*

7 Mega-Analysis Versus Meta-Analysis

7.1 Formal definitions

Define the mega-analysis phenotype

$$Y^* \;=\; \begin{cases} Y^m & \text{if } S = 1 \text{ (measured)}, \\ \hat{Y} & \text{if } S = 0 \text{ (imputed)}. \end{cases}$$

We now switch from population moments to the sample estimator. Here $i = 1, \ldots, n$ indexes individuals in the pooled sample and bars denote sample means. The mega-analysis OLS slope is

$$\hat{\beta}_p^{\text{mega}} \;=\; \frac{\sum_{i=1}^{n} (X_{pi} - \bar{X}_p)\,(Y_i^* - \bar{Y}^*)}{\sum_{i=1}^{n} (X_{pi} - \bar{X}_p)^2}\,. \tag{12}$$

One can also compute separate group-specific estimates and combine them, but we emphasize below that the mega-analysis has a clear population probability limit under phenotype-dependent selection.

7.2 A covariance identity for mixtures

We will use the identity, valid for any variables X, Y and a binary S with $\pi = \Pr(S = 1)$,

$$\operatorname{Cov}(X, Y) \;=\; \pi \operatorname{Cov}(X, Y \mid S = 1) + (1 - \pi) \operatorname{Cov}(X, Y \mid S = 0) + \pi(1 - \pi)\, \Delta_X \Delta_Y, \tag{13}$$

where $\Delta_X = \mathbb{E}[X \mid S = 1] - \mathbb{E}[X \mid S = 0]$ and $\Delta_Y = \mathbb{E}[Y \mid S = 1] - \mathbb{E}[Y \mid S = 0]$.

7.3 Mega-analysis recovers population effects under calibrated imputation

Theorem 5 (Mega-analysis consistency under phenotype-dependent selection)**.** *Let* $\pi = \Pr(S = 1)$*. Suppose Assumptions 1 and 2 hold and* $0 < \mathrm{Var}(X_p) < \infty$*,* $\mathbb{E}[Y^2] < \infty$*. Then, as* $n \to \infty$*,*

$$\operatorname{plim}\ \hat{\beta}_p^{\text{mega}} \;=\; \frac{\mathrm{Cov}(X_p, Y)}{\mathrm{Var}(X_p)}.$$

Under the working simplification assuming no LD tagging, this reduces to $\operatorname{plim}\ \hat{\beta}_p^{\text{mega}} = \beta_p$*. In the Appendix we retain general LD and show that the same argument yields the full LD expansion.*

Proof. Let $\mathbf{x}_i$ denote the regression row containing the focal SNP X_{pi} and any fixed covariates (which are residualized away in what follows). By standard derivations, $\operatorname{plim}\ \hat{\beta}_p^{\text{mega}} = \mathrm{Cov}(X_p, Y^*)/\,\mathrm{Var}(X_p)$, where Y^* is the mixture defined above. We therefore compute $\mathrm{Cov}(X_p, Y^*)$ at the population level.

First, decompose by S using (13):

$$\mathrm{Cov}(X_p, Y^*) \;=\; \pi\ \mathrm{Cov}(X_p, Y^m \mid S{=}1) + (1-\pi)\ \mathrm{Cov}(X_p, \hat{Y} \mid S{=}0) + \pi(1-\pi)\Delta_X \Delta_{Y^*}.$$

By the within-$S = 1$ restrictions in Assumption 1,

$$\mathbb{E}[Y^m \mid S = 1] = \mathbb{E}[Y \mid S = 1]$$

and

$$\mathrm{Cov}(X_p, Y^m \mid S = 1) = \mathrm{Cov}(X_p, Y \mid S = 1).$$

By the mean and SNP-covariance conditions in Assumption 2,

$$\mathbb{E}[\hat{Y} \mid S = 0] = \mathbb{E}[Y \mid S = 0]$$

and

$$\mathrm{Cov}(X_p, \hat{Y} \mid S = 0) = \mathrm{Cov}(X_p, Y \mid S = 0).$$

Therefore

$$\Delta_{Y^*} = \mathbb{E}[Y \mid S = 1] - \mathbb{E}[Y \mid S = 0] = \Delta_Y.$$

Thus

$$\mathrm{Cov}(X_p, Y^*) \;=\; \pi\ \mathrm{Cov}(X_p, Y \mid S{=}1) + (1-\pi)\ \mathrm{Cov}(X_p, Y \mid S{=}0) + \pi(1-\pi)\Delta_X \Delta_Y.$$

But the same mixture identity (13) applied directly to (X_p, Y) yields

$$\mathrm{Cov}(X_p, Y) \;=\; \pi\ \mathrm{Cov}(X_p, Y \mid S{=}1) + (1-\pi)\ \mathrm{Cov}(X_p, Y \mid S{=}0) + \pi(1-\pi)\Delta_X \Delta_Y.$$

Therefore $\mathrm{Cov}(X_p, Y^*) = \mathrm{Cov}(X_p, Y)$, and hence

$$\operatorname{plim}\ \hat{\beta}_p^{\text{mega}} \;=\; \frac{\mathrm{Cov}(X_p, Y)}{\mathrm{Var}(X_p)}.$$

□

Remark 10 (Practical implication and group-wise recentering)**.** *Theorem 5 is why the main analysis combines observed and imputed FIS in a single mega-analysis rather than by IVW meta-analysis. In practice, some implementations first recenter measured and imputed phenotypes within group before pooling (as done here), or otherwise impose a common group mean even when* $\mathbb{E}[Y \mid S{=}1] \neq \mathbb{E}[Y \mid S{=}0]$*. Doing so removes the between-group term* $\pi(1-\pi)\,\Delta_X\,\Delta_Y$ *in Equation* (13)*. The resulting per-SNP slope bias is*

$$\text{plim } \hat{\beta}_{p,forced} - \beta_p^{\text{marg}} = -\frac{\pi(1-\pi)\,\Delta_{X_p}\,\Delta_Y}{\text{Var}(X_p)}.$$

$$\textit{where } \Delta_{X_p} = \mathbb{E}[X_p \mid S{=}1] - \mathbb{E}[X_p \mid S{=}0].$$

The size of this bias depends jointly on Δ_Y *and on genotype-frequency differences between the measured and imputed groups through* Δ_{X_p}*. It is expected to be small when selection mainly acts through highly polygenic differences in* Y*, because each individual SNP contributes little to* Δ_{X_p}*, but it need not be small if measured and imputed groups differ for reasons tied to residual population structure or other ancestry-correlated factors, such that allele frequencies differ substantially between measured and imputed individuals. In our empirical application we reduce this risk by restricting to a relatively homogeneous ancestry subset, adjusting for genetic principal components, checking that combined FIS does not show an altered* r_g *profile with a large panel of traits relative to an independent intelligence GWAS, and checking that* $r(\textit{direct}, \textit{NTC})$ *attenuates toward* 0*. We therefore treat any remaining bias from group-wise recentering as likely limited in this application.*

7.4 Why inverse-variance meta-analysis does not, in general, recover the pooled population association

For group $s \in \{0, 1\}$, let

$$\theta_{p,s} \equiv \text{plim } \hat{\beta}_p^{S=s} = \frac{\text{Cov}(X_p, Y \mid S = s)}{\text{Var}(X_p \mid S = s)},$$

where the equality follows for the measured and imputed outcomes under Assumptions 1 and 2, respectively.

With general LD, write

$$T_{p,s} = \sum_{q \neq p} \beta_q \frac{\text{Cov}(X_q, X_p \mid S = s)}{\text{Var}(X_p \mid S = s)}, \qquad C_{p,s} = \frac{\text{Cov}(E, X_p \mid S = s)}{\text{Var}(X_p \mid S = s)}.$$

Then

$$\theta_{p,s} = \beta_p + T_{p,s} + C_{p,s}.$$

Let

$$T_p = \sum_{q \neq p} \beta_q \frac{\text{Cov}(X_q, X_p)}{\text{Var}(X_p)},$$

so that

$$\beta_p^{\text{marg}} = \beta_p + T_p.$$

It follows that

$$\theta_{p,s} = \beta_p^{\text{marg}} + d_{p,s}, \qquad d_{p,s} = (T_{p,s} - T_p) + C_{p,s}.$$

Suppose the normalized inverse-variance weights converge to $\omega_{p,1}$ and $\omega_{p,0}$, where $\omega_{p,1} + \omega_{p,0} = 1$. Then

$$\text{plim } \hat{\beta}_p^{\text{meta}} = \omega_{p,1}\theta_{p,1} + \omega_{p,0}\theta_{p,0} = \beta_p^{\text{marg}} + \omega_{p,1} d_{p,1} + \omega_{p,0} d_{p,0}.$$

Recovery of the population marginal coefficient would therefore require

$$\omega_{p,1}d_{p,1} + \omega_{p,0}d_{p,0} = 0.$$

No assumption in the present framework imposes this identity. The inverse-variance weights are determined by the group-specific sampling variances, whereas $d_{p,s}$ is determined by changes in LD tagging and by collider-induced SNP–residual covariance. Thus inverse-variance meta-analysis does not, in general, reproduce the pooled population marginal association.

Under the no-LD working simplification used in Theorem 4, $T_{p,s} = T_p = 0$ and hence $d_{p,s} = C_{p,s} = b_{p,s}$. Even in that special case, cancellation would require

$$\omega_{p,1}b_{p,1} + \omega_{p,0}b_{p,0} = 0,$$

which is not guaranteed by the stated assumptions.

8 Heteroskedastic Regression Framework

Throughout this section, all variables are understood to have been adjusted using the same pooled set of fixed GWAS covariates. For each focal SNP p, let

$$\mathbf{q}_{pi} = (1, X_{pi}, \mathbf{Z}_i^\top)^\top,$$

where $\mathbf{Z}_i$ contains the fixed adjustment covariates. Define the population linear-projection coefficient vector by

$$\boldsymbol{\theta}_p^* = \arg\min_{\boldsymbol{\theta}} \mathbb{E}\left[\left(Y_i^* - \mathbf{q}_{pi}^\top\boldsymbol{\theta}\right)^2\right],$$

and define the corresponding projection residual by

$$u_{pi} = Y_i^* - \mathbf{q}_{pi}^\top\boldsymbol{\theta}_p^*.$$

By the normal equations,

$$\mathbb{E}[\mathbf{q}_{pi}u_{pi}] = \mathbf{0}.$$

Under Assumptions 1 and 2, and under the common residualization convention stated above, the focal-SNP component of $\boldsymbol{\theta}_p^*$ equals the corresponding population coefficient obtained using the latent phenotype Y, subject to the group-centering qualification.

Because Y_i^* is generated from different sources, the conditional variance of u_{pi} will generally differ between measured and imputed individuals. Within the measured group it may also differ with the number of replicate measurements used to construct Y_i^m. Thus

$$\mathrm{Var}(u_{pi} \mid \mathbf{q}_{pi})$$

need not be constant across individuals. Equality of these conditional variances is possible in special cases, so the errors are more precisely described as generally, rather than logically necessarily, heteroskedastic.

We assume independent sampling across individuals, finite fourth moments, a nonsingular matrix $\mathbb{E}[\mathbf{q}_{pi}\mathbf{q}_{pi}^\top]$, and a fixed number of adjustment covariates as $n \to \infty$.

8.1 Robust inference with sandwich standard errors

Assumptions 1 and 2 give the covariance restrictions needed for consistency of the GWAS slope estimates, with population limits given in Theorem 5. For inference under heteroskedasticity, we use the standard HC3 (leverage-adjusted) heteroskedasticity-consistent variance estimator. If $\mathbf{Q}_p$ is the design matrix with rows $\mathbf{q}_{pi}^\top$, let

$$\mathbf{H}_p = \mathbf{Q}_p(\mathbf{Q}_p^\top \mathbf{Q}_p)^{-1}\mathbf{Q}_p^\top$$

and let $h_{p,ii}$ denote its ith diagonal entry. Then

$$\widehat{\mathrm{Var}}_{\mathrm{HC3}}(\hat{\boldsymbol{\theta}}_p) = (\mathbf{Q}_p^\top \mathbf{Q}_p)^{-1}\left(\sum_{i=1}^{n} \frac{\hat{u}_{pi}^2}{(1-h_{p,ii})^2}\mathbf{q}_{pi}\mathbf{q}_{pi}^\top\right)(\mathbf{Q}_p^\top \mathbf{Q}_p)^{-1}.$$

where $\hat{\boldsymbol{\theta}}$ collects the estimated coefficient on the focal SNP and the coefficients on the adjustment covariates.

Remark 11 (Connection to empirical analysis)**.** *This is why the population GWAS analyses of the measured and combined FIS phenotypes use heteroskedasticity-robust standard errors in Regenie.*

9 SNP Heritability Under Mixed Measurement and Imputation

Recall that

$$Y^* = SY^m + (1-S)\hat{Y},$$

so that $Y^* = Y^m$ for measured individuals and $Y^* = \hat{Y}$ for imputed individuals. We use G_{tagged} to denote the additive genetic component tagged by the analyzed SNPs, as defined in Equations (1)–(2).

Throughout this section, we maintain Assumptions 1 and 2. For the variance calculation, we additionally assume that a measured phenotype based on k_i replicates can be written as

$$Y_i^m = Y_i + \bar{\epsilon}_{i,k_i}^m, \qquad \mathrm{Var}(\bar{\epsilon}_{i,k_i}^m \mid k_i, S_i = 1) = \frac{\sigma_\epsilon^2}{k_i},$$

where the replicate errors are conditionally mean-zero, mutually independent, and orthogonal to the latent phenotype and analyzed genotypes. We also assume that all relevant second moments are finite and write

$$m(\mathbf{W}) = \mathbb{E}[Y \mid \mathbf{W}, S = 0], \qquad \sigma_\delta^2 = \mathrm{Var}(\delta \mid S = 0).$$

Let $\pi = \Pr(S = 1)$ and

$$\Delta_{Y^*} = \mathbb{E}[Y^* \mid S = 1] - \mathbb{E}[Y^* \mid S = 0].$$

The mixture-variance identity therefore gives

$$\begin{aligned}\mathrm{Var}(Y^*) = \pi &\left\{\mathrm{Var}(Y \mid S = 1) + \mathbb{E}\left[\frac{\sigma_\epsilon^2}{k_i} \,\middle|\, S = 1\right]\right\} \\ &+ (1-\pi)\left\{\mathrm{Var}\big(m(\mathbf{W}) \mid S = 0\big) + \sigma_\delta^2 + 2\,\mathrm{Cov}\big(m(\mathbf{W}), \delta \mid S = 0\big)\right\} + \pi(1-\pi)\Delta_{Y^*}^2. \quad (14)\end{aligned}$$

If measured and imputed phenotypes are separately centered before pooling, the final between-group term is absent. As discussed in Remark 10, exact preservation of per-SNP effects after such group-wise centering additionally requires $\Delta_{X_p} = 0$ after covariate adjustment for every analyzed SNP; otherwise the centering-induced bias derived in that remark applies.

Under the covariance-preservation conditions of Theorem 5, and in the absence of centering-induced bias, the observed-scale SNP coefficients and hence the tagged-genetic numerator are preserved. On the observed scale,

$$h^2_{\mathrm{SNP}}(Y^*) = \frac{\mathrm{Var}(G_{\mathrm{tagged}})}{\mathrm{Var}(Y^*)}.$$

Holding the other components of Equation (14) fixed, increasing the number of measured replicates or reducing σ^2_δ reduces the phenotype variance attributable to error and therefore increases observed-scale SNP heritability. Conversely, a lower observed-scale h^2_{SNP} for the combined phenotype can be consistent with additional measurement or imputation error. The decomposition also makes clear that the change is not uniquely determined by error variance, because the variance of the imputed conditional-mean component $m(\mathbf{W})$ and any group-wise centering operation also contribute. This is the interpretation relevant to the modest attenuation of h^2 from observed FIS to combined FIS in Figure 1B of the main paper.

10 Evaluating Imputation via Genetic Correlations with LDSC

This section derives what LDSC cross-trait regression should estimate for the mega-analyzed phenotype Y^* when imputation adds noise but introduces no systematic genetically correlated signal with other traits. It follows that a mega-analysis phenotype that truly targets Y will reproduce the r_g profile from an independent intelligence GWAS.

10.1 LDSC set-up and invariance under calibrated imputation

For trait $t \in \{1, 2\}$, let $\mathbf{y}_t$ be the N_t-vector of residualized, unit-variance phenotypes. Let $\mathbf{x}$ denote the vector of standardized SNP genotypes in a common reference population and let

$$\mathbf{R} = \mathrm{Cov}(\mathbf{x})$$

be the corresponding LD matrix. Write the tagged genetic component of trait t as

$$g_t = \mathbf{x}^\top \boldsymbol{\alpha}_t,$$

where $\boldsymbol{\alpha}_t$ is a vector of standardized-scale joint projection effects. Define

$$h^2_t = \mathrm{Var}(g_t) = \boldsymbol{\alpha}_t^\top \mathbf{R} \boldsymbol{\alpha}_t,$$

$$\rho_g = \mathrm{Cov}(g_1, g_2) = \boldsymbol{\alpha}_1^\top \mathbf{R} \boldsymbol{\alpha}_2,$$

and

$$r_g = \frac{\rho_g}{\sqrt{h^2_1 h^2_2}}.$$

For SNP j, let z_{tj} be the marginal GWAS z statistic and let

$$\ell_j = \sum_{k=1}^{M} r^2_{jk}$$

be its LD score. Under the standard LDSC random-effects assumptions, including the assumptions connecting effect-size second moments to LD scores, and suppressing intercept terms due to confounding or sample overlap,

$$\mathbb{E}[z_{tj}^2 \mid \ell_j] \approx 1 + \frac{N_t h_t^2}{M}\ell_j,$$

and

$$\mathbb{E}[z_{1j} z_{2j} \mid \ell_j] \approx \frac{\sqrt{N_1 N_2}\rho_g}{M}\ell_j.$$

Theorem 6 (Tagged genetic components and genetic correlations under calibrated imputation)**.** *Suppose Y^* is formed from Y^m and $\hat{Y}$ as above, and Assumptions 1 and 2 hold for every analyzed SNP. Then the population linear projection of Y^* onto the analyzed genotype space is the same as the population linear projection of Y onto that space. Consequently, if*

$$c = \sqrt{\frac{\mathrm{Var}(Y)}{\mathrm{Var}(Y^*)}},$$

the tagged genetic component on the unit-variance scale satisfies

$$g_{Y^*} = c\, g_Y.$$

It follows that

$$h^2_{\mathrm{SNP}}(Y^*) = c^2 h^2_{\mathrm{SNP}}(Y),$$

and, for any external trait T with positive tagged genetic variance,

$$\rho_g(Y^*, T) = c\,\rho_g(Y, T), \qquad r_g(Y^*, T) = r_g(Y, T).$$

Proof. Assumption 2, together with the corresponding measurement-error restrictions in the measured group, implies

$$\mathrm{Cov}(\mathbf{X}, Y^*) = \mathrm{Cov}(\mathbf{X}, Y),$$

where $\mathbf{X}$ is the vector of analyzed SNPs. Therefore Y^* and Y have the same normal equations for their population linear projections onto the genotype span. Their fitted tagged genetic components on the original phenotype scale are consequently identical.

Standardizing Y^* and Y divides their common tagged genetic component by $\mathrm{sd}(Y^*)$ and $\mathrm{sd}(Y)$, respectively. Hence the unit-variance tagged component for Y^* equals

$$c = \frac{\mathrm{sd}(Y)}{\mathrm{sd}(Y^*)}$$

times that for Y. Genetic variance therefore scales by c^2, and genetic covariance with an external trait scales by c. These factors cancel in the normalized ratio defining r_g. □

Remark 12 (Connection to empirical analysis)**.** *This theorem is the main reason the paper treats the external r_g profile as a specification check. The relevant empirical comparisons are the preservation of the r_g pattern in Figure 1C and Extended Data Figure 3, together with the modest drop in observed-scale h^2 in Figure 1B. Systematic deviations would instead suggest that the imputation imported genetically correlated non-target signal.*

11 Conclusions and Implications

This note formalizes when and why a pooled ("mega-analysis") GWAS of observed and imputed phenotypes recovers the same per-SNP population effects as if the latent trait Y were observed for all individuals. Assumptions 1 and 2 are the core assumptions for that claim and for the empirical combined-FIS analyses. By contrast, Assumptions 3, 4, and 5 are used only in the selection subsection to obtain interpretable within-group collider formulas. Two features of the set-up are key. First, we distinguish *measurement error*, arising from classical instrument noise (Section 2.2), from *imputation error*, arising from the construction of $\hat{Y}$. Instrument noise ϵ^m is ancillary for identification of SNP effects: it inflates residual variance but, under the stated conditions, does not bias slopes. By contrast, the adequacy of the imputation is summarized by the calibration conditions in Assumption 2. Second, we make explicit how phenotype-dependent selection into "measured" status can induce collider terms within S=1 and S=0 (Theorem 2), yet cancels in the mixture when one directly regresses the pooled phenotype Y^* on genotypes (Theorem 5). Inference then proceeds robustly with sandwich standard errors to accommodate the inherent heteroskedasticity of the mixture.

A related conceptual distinction is that measurement error is a property of the measurement instrument, whereas imputation error is a property of the imputation rule together with the joint distribution of $(Y, \mathbf{W}, X, S)$. The former admits a compact correction, for example translating an imputation accuracy r_{imp} into a latent correlation via $r_{\text{imp}}/\sqrt{\rho}$ (Theorem 1). The latter admits no universal correction: its adequacy is established by design (choosing $\mathbf{W}$ to target Y rather than the idiosyncrasies of Y^m) and diagnosis (checking genetic invariances predicted by the theory).

The LDSC consequences clarify this diagnostic. Because the observed-scale marginal per-SNP effects are identical for Y and Y^* under Assumption 2, the tagged-genetic numerator is preserved under the stated assumptions and in the absence of group-centering distortion, while observed-scale heritability may be attenuated by the measurement and imputation components of $\text{Var}(Y^*)$, while the observed-scale SNP covariance pattern is unchanged and the LDSC genetic correlation with an external trait W is unchanged (Theorem 6). Thus cross-trait $r_g(Y^*, W)$ should agree with $r_g(Y, W)$, whereas $h^2_{\text{SNP}}(Y^*)$ can be modestly smaller than $h^2_{\text{SNP}}(Y)$ due to measurement/imputation noise in the denominator (Section 9). In the empirical application, the pooled "Combined FIS" shows this pattern: a modest reduction in observed-scale h^2 relative to measured FIS (approximately 0.16 versus 0.20 to 0.23) together with a cross-trait r_g profile that closely mimics external cognition GWAS across many traits; at the same time, the enlarged N substantially increases locus and gene discovery power, all as predicted by the theory.

Where might the LDSC invariances fail? If the imputation imports genetically correlated non-target signal (e.g., by including auxiliary variables that capture education-specific or anthropometric correlates of FIS), then the pooled GWAS can show inflated r_g with such external traits even as r_{imp} increases. The empirical remedy is to curate $\mathbf{W}$ to target the cognitive signal and to verify that the r_g profile with respect to canonical controls (e.g., height; non-cognitive EA) remains stable; in practice, paring the auxiliary set from 154 to 82 variables achieves exactly this without sacrificing much accuracy. Combining measured and imputed phenotypes also introduces assumptions regarding the relative means and variances of the phenotype between observed and imputed individuals. Re-scalings that differ between groups (e.g., enforcing equal means across S) introduce a between-group term that can bias SNP effect estimates. However, this would also alter patterns of genetic correlations with other traits, offering an indirect means of assessing that assumption.

Taken together, the theory yields a practical guideline for constructing and validating imputed phenotypes for GWAS. Build the imputation to satisfy the calibration condition in Assumption 2, combine observed and imputed values in a single regression with heteroskedasticity-consistent er-

rors, and treat LDSC cross-trait correlations as a specification check that should be preserved even as observed-scale heritability attenuates with added noise. The empirical results align with these predictions: the pooled analysis increases discovery (common and rare variants) while maintaining a genetic correlation profile that matches external intelligence GWAS, consistent with adding mostly genotype-uncorrelated imputation noise rather than a different genetic construct.

However, it is important to note that the stated assumptions are impossible to test fully in practice; one must rely on downstream consequences of their violations. We therefore caution against treating mega-analyzed phenotypes as interchangeable with direct measurements. External validation of the GWAS results in downstream applications such as PGI and tissue-enrichment analyses should be used to provide additional evidence regarding the validity of the imputation.

Appendix: Full LD-tagging derivations and mega-analysis cancellation

Throughout the appendix we retain all standing assumptions and notation from the main text. $\mathbf{X} = (X_1, \ldots, X_P)^\top$ denotes the centered genotype vector.

Group-specific marginal slopes with LD. Fix $s \in \{0, 1\}$. Using $Y = \sum_q \beta_q X_q + E$,

$$\beta_p^{\text{marg}|S=s} = \frac{\text{Cov}(X_p, Y \mid S = s)}{\text{Var}(X_p \mid S = s)} = \beta_p + \underbrace{\sum_{q \neq p} \beta_q \frac{\text{Cov}(X_q, X_p \mid S = s)}{\text{Var}(X_p \mid S = s)}}_{\text{LD tagging } T_{p,s}} + \underbrace{\frac{\text{Cov}(E, X_p \mid S = s)}{\text{Var}(X_p \mid S = s)}}_{\text{collider } C_{p,s}}. \quad (15)$$

A weighted identity. Multiplying (15) by $\beta_p \, \text{Var}(X_p \mid S = s)$ and summing over p gives

$$\sum_{p=1}^{P} \beta_p \, \text{Var}(X_p \mid S = s) \left(\beta_p^{\text{marg}|S=s} - \beta_p\right) = \underbrace{\sum_{p \neq q} \beta_p \beta_q \, \text{Cov}(X_q, X_p \mid S = s)}_{\mathcal{L}_s \text{ (pure LD term)}} + \underbrace{\text{Cov}(E, G \mid S = s)}_{\mathcal{C}_s \text{ (collider term)}}. \quad (16)$$

Recovery of the population LD-tagging association in the mega-analysis. Let $\pi = \Pr(S{=}1)$ and recall the mega phenotype Y^* equals Y^m in $S{=}1$ and $\hat{Y}$ in $S{=}0$. Using the mixture identity (13) from the main text,

$$\text{Cov}(X_p, Y^*) = \pi \, \text{Cov}(X_p, Y^m \mid S{=}1) + (1 - \pi) \, \text{Cov}(X_p, \hat{Y} \mid S{=}0) + \pi(1 - \pi) \, \Delta_{X_p} \, \Delta_{Y^*},$$

where $\Delta_Z = \mathbb{E}[Z \mid S{=}1] - \mathbb{E}[Z \mid S{=}0]$. Because $\mathbb{E}[Y^m \mid S{=}1] = \mathbb{E}[Y \mid S{=}1]$ and, by Assumption 2, $\text{Cov}(X_p, \hat{Y} \mid S{=}0) = \text{Cov}(X_p, Y \mid S{=}0)$, we have $\Delta_{Y^*} = \Delta_Y$ and

$$\begin{aligned} \text{Cov}(X_p, Y^*) &= \pi \, \text{Cov}(X_p, Y \mid S{=}1) + (1 - \pi) \, \text{Cov}(X_p, Y \mid S{=}0) + \pi(1 - \pi) \, \Delta_{X_p} \, \Delta_Y \\ &= \sum_q \beta_q \Big[\pi \, \text{Cov}(X_q, X_p \mid S{=}1) + (1 - \pi) \, \text{Cov}(X_q, X_p \mid S{=}0)\Big] \\ &\quad + \Big[\pi \, \text{Cov}(E, X_p \mid S{=}1) + (1 - \pi) \, \text{Cov}(E, X_p \mid S{=}0)\Big] + \pi(1 - \pi) \, \Delta_{X_p} \, \Delta_Y. \quad (17) \end{aligned}$$

Apply the same mixture identity (13) to the pairs (X_q, X_p) and (E, X_p):

$$\text{Cov}(X_q, X_p) = \pi \, \text{Cov}(X_q, X_p \mid S{=}1) + (1 - \pi) \, \text{Cov}(X_q, X_p \mid S{=}0) + \pi(1 - \pi) \, \Delta_{X_q} \Delta_{X_p},$$

$$\mathrm{Cov}(E, X_p) = \pi\ \mathrm{Cov}(E, X_p \mid S{=}1) + (1-\pi)\ \mathrm{Cov}(E, X_p \mid S{=}0) + \pi(1-\pi)\,\Delta_E\Delta_{X_p},$$

and note $\Delta_Y = \Delta_G + \Delta_E$ with $\Delta_G = \sum_q \beta_q \Delta_{X_q}$. Substituting these two identities into (17) yields

$$\begin{aligned}\mathrm{Cov}(X_p, Y^*) = \sum_q \beta_q \Big[\,\mathrm{Cov}(X_q, X_p) - \pi(1-\pi)\,\Delta_{X_q}\Delta_{X_p}\Big] \\ +\ \Big[\mathrm{Cov}(E, X_p) - \pi(1-\pi)\,\Delta_E\Delta_{X_p}\Big]\ +\ \pi(1-\pi)\,\Delta_{X_p}\,\big(\Delta_G + \Delta_E\big).\end{aligned}$$

The cross terms cancel because

$$-\sum_q \beta_q\,\Delta_{X_q}\,\Delta_{X_p}\ -\ \Delta_E\,\Delta_{X_p}\ +\ \Delta_G\,\Delta_{X_p}\ +\ \Delta_E\,\Delta_{X_p}\ =\ 0.$$

Therefore

$$\mathrm{Cov}(X_p, Y^*) = \sum_q \beta_q\ \mathrm{Cov}(X_q, X_p) + \mathrm{Cov}(E, X_p) = \mathrm{Cov}(X_p, Y),$$

so that

$$\mathrm{plim}\ \hat{\beta}_p^{mega}\ =\ \frac{\mathrm{Cov}(X_p, Y^*)}{\mathrm{Var}(X_p)}\ =\ \frac{\mathrm{Cov}(X_p, Y)}{\mathrm{Var}(X_p)}\ =\ \beta_p + \sum_{q\neq p} \beta_q \frac{\mathrm{Cov}(X_q, X_p)}{\mathrm{Var}(X_p)}.$$

Thus, the pooled regression reproduces the full population LD expansion for SNP p; any distortions of LD within $S{=}1$ and $S{=}0$ (and any collider paths) cancel in the mixture once the imputed group is calibrated as in Assumption 2.

Supplementary Note 3: Comparison of imputation strategies

We evaluated multiple strategies to impute missing fluid intelligence scores in UK Biobank. In three iterations we refined our imputation approach and evaluated each iteration using imputation accuracy, population-based and within-family SNP heritability, genetic correlations with external traits, and correlations between direct effects and NTC estimates from the within-family GWAS (See Methods). Below, we describe each imputation approach in turn and explain how these evaluations informed our final decision.

Initial round of imputation focusing on the baseline FIS measure

In the first iteration of FIS imputation we sought to impute individuals without a baseline FIS (FIS1) measurement using 154 correlated variables that had lower missingness (e.g., educational attainment, time spent on a computer, and household income) (Supplementary Table 4). This imputation achieved an accuracy of r=0.52 (SE = .006) (Supplementary Figure 4A, Supplementary Table 5), which is slightly lower than the test-retest correlation of FIS we found in UK Biobank (r ≈ 0.6).

Measured FIS1 (FIS1, N=146,526) and imputed FIS (N=309,128) were then standardized separately and mega-analysed in a single GWAS (MegaFIS-vA, N=455,654), which we found performed better than meta-analyzing separate GWASs of measured and imputed FIS1 (Supplementary Note 2 and 4; Supplementary Figure 6; Supplementary Table 6). MegaFIS-vA had an estimated h^2_{pop} of 0.18 (SE = .005), which was similar to that of Savage et al. Intelligence (h^2_{pop}=0.19) though significantly lower than that for FIS1 (h^2_{pop}=0.23; p = 1.07×10^{-6}, z-test). Subsequently, we computed genetic correlations between 68 traits and MegaFIS-vA and compared the genetic correlation profile to that derived by correlating the COGENT GWAS with those same 68 traits (Supplementary Figure 3). We noted that MegaFIS-vA showed higher genetic correlations with socioeconomic-related variables such as non-cognitive educational attainment (~0.33 versus ~0.2) and height (~0.23 versus ~0.05) than COGENT (Supplementary Figure 4D). The within-family GWAS on MegaFIS-vA showed that the h^2_{direct} was about a third lower than the heritability obtained in the population-based GWAS (Supplementary Figure 4C). We hypothesized that these results may be due to some of the selected imputation variables correlating with measured FI via paths not mediated by the latent FI construct of interest (see Supplementary Note 2 for further discussion).

Refining imputation variables to attempt to reduce “non-cognitive” signal

To reduce the “non-cognitive” educational attainment signal being introduced by some of the imputation variables, and to more closely reflect the intended latent fluid intelligence trait, we refined the set of imputation variables based on whether their genetic signal aligned more strongly with cognitive ability than with non-cognitive correlates of intelligence. We used GWAS-by-subtraction[5] (Supplementary Figure 7) to subtract the signal of the Savage et al. Intelligence GWAS from our GWAS on imputed FIS (N=309,128). This resulted in a GWAS of “non-cognitive imputed FIS” (NonCog-iFIS). The NonCog-iFIS GWAS captured signals associated with imputed FIS that are not observed in the Savage et al. Intelligence GWAS, and thus likely reflected confounding due to correlations between FIS1 and e.g. socioeconomic variables. We then calculated genetic correlations between the aforementioned 154 imputation variables used in our initial round of imputation and both

NonCog-iFIS and the Savage et al. intelligence GWAS (Supplementary Figure 8, Supplementary Table 4). Based on these results, we refined the set of imputation variables to a subset of 82 variables that showed a stronger genetic correlation with intelligence than with NonCog-iFIS.

These variables were then used for a new imputation of FIS1, achieving an accuracy of r = 0.49 (SE=.006), which was comparable to (albeit slightly lower) than that obtained in the first imputation (Supplementary Table 5, Supplementary Figure 4A). We again combined imputed FIS with measured FIS1 in a GWAS via mega-analysis (MegaFIS-vB). The resulting GWAS had an estimated h^2_{pop} = 0.17 (SE = .005) (Supplementary Figure 4C), similar to that obtained with MegaFIS-vA. Additionally, MegaFIS-vB had a similar pattern of r_g with external trait GWASs as MegaFIS-vA, except that it had a substantially lower r_g with height (Supplementary Figure 4D). However, the elevated genetic correlation with NonCogEA remained (r_g=0.34, SE=.02) and after conducting a within-family GWAS of MegaFIS-vB, we estimated h^2_{direct} to be about three-quarters that of population-based GWAS (Supplementary Figure 4C), suggesting the imputed phenotype still contained substantial confounding signal.

Using additional FIS measures to further reduce "non-cognitive" signal

In the final iteration, we tried three different strategies to further refine the imputation and reduce the "non-cognitive" signal, by including additional UKB FIS measures (FIS2-5). In the first two strategies, we put either the average or the earliest of FIS measures available for a UKB participant into the imputation as single variables, alongside the 82 variables used above. Imputed values (for participants without any FIS measure) were then taken for downstream analysis. In the third strategy, we put all five FIS measures into the imputation as separate variables, alongside the aforementioned 82 variables ("all5" strategy). In this approach, values across all 5 FIS measures were imputed but we only took imputed values for FIS1 into downstream analyses (Supplementary Figure 5). These three strategies produced phenotypic accuracies of ~0.45-0.55, similar to the first two imputation attempts (Supplementary Figure 4A), the highest accuracy coming from the imputation including all five FIS measures. It is important to note, however, that the overall accuracy of this "all5" imputation is inflated because some of the individuals who were imputed for FIS1 had other FIS measures, and can thus be imputed more accurately (Supplement 4, Supplementary Table 5). In all approaches, the highest accuracy was observed for individuals in the top and bottom terciles of the measured phenotype distribution (Supplementary Figure 9).

We then conducted a GWAS mega-analysis of each of these imputation approaches with their respective observed parts: Average observed FIS with imputed average FIS (MegaFIS-average); Earliest observed FIS with imputed earliest FIS (MegaFIS-earliest); observed FIS1 with imputed FIS from the "all5" strategy (MegaFIS-all5). Imputation increased the sample size to around 455,400 individuals with estimated h^2_{pop} values of 0.15 (SE = .005) for MegaFIS-earliest, 0.16 for MegaFIS-average (SE = .005), and 0.17 (SE = .005) for MegaFIS-all5 (Supplementary Figure 4D). We also conducted within-family GWAS mega-analyses on the three phenotypes and found minimal attenuation of point estimates of h^2_{direct} relative to h^2_{pop} (Supplementary Figure 4C), consistent with the results for the measured phenotypes. For all three of these imputation approaches, the genetic correlation with NonCogEA attenuated to ~0.16 (SD=0.02), which was comparable with the r_g estimated using the COGENT cognition GWAS[1,2] (r_g = 0.20, SE=.04) (Supplementary Figure 4D), and the r_g with height remained in

line with that using measured FIS1 (r_g=0.11, SE = .02). Lastly, we found that MegaFIS-average and MegaFIS-earliest yielded a correlation between direct genetic effects and non-transmitted coefficient (r(direct,NTC)) which was not significantly different from 0 (Supplementary Figure 4G). MegaFIS-all5 had a significant negative r(direct-NTC), so we decided not to take this "all5" imputation strategy forward into downstream analyses. Collectively, these results suggested that the MegaFIS-average and MegaFIS-earliest imputation approaches yielded traits that were highly genetically similar to the measured FIS while reducing ascertainment biases. We observed a slightly higher phenotypic imputation accuracy, more significant lead SNPs, and slightly higher h^2_{pop} for MegaFIS-average than for MegaFIS-earliest, so decided to use that for the main downstream analyses.

Supplementary Note 4: Combining measured and imputed data

The goal of imputation was to combine the measured and imputed values to obtain a single GWAS with the largest effective sample size possible. We examined whether combining them should be done by meta- or mega-analysis by applying both strategies to the imputed and measured values from the last three imputation approaches i.e. imputed earliest FIS, imputed average FIS and All 5 imputed FIS. For the mega-analyses, we scaled imputed and measured values separately before combining them and then ran a GWAS as described in the Methods. In the meta-analysis we used GWASs conducted on imputed and measured values separately and then meta-analyzed them using METAL[6] following the procedure described in the Methods. Ultimately, we ended up with six GWASs: a meta- and mega-analysis derived-GWAS for each of the three imputation approaches.

Generally, scaling measured and imputed values separately and combining them by mega-analysis showed better results than meta-analysis across all approaches, namely lower genetic correlations with NonCogEA and higher genetic correlations with the measured intelligence GWASs (Supplementary Figure 6). In addition, the mega-analyses also demonstrated higher heritability estimates (Supplementary Table 6), and showed better out-of-sample prediction when PGI constructed with their summary statistics were used to predict IQ between and within families in an external cohort (Supplementary Table 9). Furthermore, mega-analysis retains desirable statistical properties, such as correcting for ascertainment biases under certain conditions, which meta-analysis does not, making it a more principled analytical approach (Supplementary Note 2). When combined with the observed measures via mega-analysis, the three imputation strategies performed extremely similarly in terms of their genetic correlations with other traits.

We acknowledge that this mega-analysis approach of scaling the measured and imputed values separately and then combining them is flawed in that it is unlikely that these two groups truly have the same mean FI, given that they differ in average educational attainment (Supplementary Figure 2). The imputed FI scores show considerably less variance than the measured scores, as a consequence of the imputation tending to push people towards the middle of the distribution. In theory, the meta-analysis approach gets around these differences in mean and variance between the observed and imputed values, but since in practice, we found that the mega-analysis worked slightly better, we decided to use this for downstream analyses.

Supplementary Note 5: Performance of the All 5 imputed FIS strategy in different subsets

Combining measurements before imputation (as in the imputed average FIS and imputed earliest FIS approaches) causes a discrepancy in what is imputed between these approaches and the approach in which all FIS measures are included in imputation (imputed All5 FIS). In the imputed average and imputed earliest FIS approaches, only individuals without any FIS will be imputed, whereas in the imputed All5 FIS approach, all individuals without a FIS1 score are imputed, including those that have scores in FIS2-5. To be able to directly compare imputed FIS across approaches, we partitioned imputed All 5 FIS individuals into two groups (Supplementary Figure 5): (i) people who *do* have a score available, but not at FIS1 (ever-measured); and (ii) people *without any* available score (never-measured). This allows the never-measured group to be compared fairly with the imputed average- and earliest FIS approaches.

We compared these subsets based on imputation accuracies, estimated SNP h^2, and r_g with NonCogEA and Savage et al. Intelligence (Supplementary Table 5 & 7). Although imputed All5 FIS appeared to perform better overall (most notably having higher imputation accuracy, lower r_g with NonCogEA and higher r_g with Intelligence), we saw that distinguishing between ever-measured and never-measured individuals attenuates this advantage. While the ever-measured group shows r_g and estimated SNP h^2 values similar to measured value GWASs, the never-measured group shows r_g and estimated SNP h^2 values in line with those of imputed average and imputed earliest FIS. These findings indicated that all imputation approaches work similarly when evaluated within the same set of individuals (i.e. those with no FIS measure), which allowed for appropriate comparisons of corresponding mega-analyses. Consequently, we decided to proceed with MegaFIS-average in downstream analysis.

Supplementary Note 6: Power gains from increasing GWAS sample size

We carried out GWASs of observed FIS1 (N=146,467), Average observed FIS (referred to as "observed FIS" in the main text; N=283,418), MegaFIS-average (referred to as "combined FIS" in the main text; N=455,402) and MegaFIS-average meta-analysed with COGENT ("combined FIS+COGENT" in the main text; N=490,700), and compared the results from these. The number of prioritized genes (FDR<1%; Supplementary Table 8) increased substantially and monotonically with the size of the GWAS (Figure 2A), indicating that the integration, imputation, and cross-cohort meta-analytic approaches each increased power. In particular, the number of prioritized genes increased by a factor of 3.6 between the observed FIS1 (smallest) and cross-cohort (largest) analyses. The vast majority of genes were prioritized by at least two different GWAS. MAGMA gene-based p-values were then used to assess enrichment for expression in 54 GTEx tissues (Figure 2B; Supplementary Table 10). Across each GWAS, prioritized genes were only significantly ($p<0.05/54$) enriched in brain tissues, and the degree of enrichment became more significant as the GWAS sample size increased, suggesting that the integration of missing FI measures was producing a biologically-relevant phenotype.

We derived PGIs based on the various GWAS and tested them in three independent UK-based cohorts with genetically-inferred European ancestries (Supplementary Methods). Specifically, we examined IQ at age 8 in the Avon Longitudinal Study of Parents and Children (ALSPAC; N=5,283)[7,8], a combined measure of cognitive performance in early childhood (ages 3-7) in the Millennium Cohort Study[9] (MCS; N=5,621), and the same FI measure as UKB in the INTERVAL study of blood donors (N=20,328)[9]. We observed significant associations between all PGIs and the intelligence measure in all cohorts, with the greatest variance explained in ALSPAC, likely because the measure of intelligence used there (the Wechsler Intelligence Scale Test) has lower measurement error than the measures used in MCS and INTERVAL. In all three cohorts, the variance explained by the PGIs increased monotonically with sample size. (Figure 2C, Supplementary Table 9). The same trend was observed for direct effect estimates obtained by controlling for parental PGIs in ALSPAC and MCS, suggesting the increased variance explained reflects improved prediction of direct genetic effects. This association between discovery GWAS log sample size and predictive power of the PGI was significant in a multilevel mixed effects model when considering both the population and direct effect estimates (beta = 0.0374, p = 1.64×10^{-10}) or the direct effect estimates alone (beta = 0.058, p = 0.0011). These results suggest that the combined FIS GWAS resulted in increased power for genetic prediction of measured cognitive ability in external cohorts, providing additional validation for the use of imputed FI in genetic studies.

Supplementary Note 7: Rare variant analyses

Exome-wide burden analyses

The burden of rare (MAF<0.001%) PTVs and damaging missense variants was negatively associated with average observed FIS and with imputed average FIS (Supplementary Figure 10). As expected, rare PTVs and damaging missense variants in constrained genes (pLI>0.9) showed stronger associations than all such variants exome-wide (Supplementary Figure 10). However, the exome-wide burden of rare synonymous variants, which we expect to show no association, was significantly positively associated with both imputed and observed average FIS, with a significantly larger effect size for imputed average FIS (Supplementary Figure 10). This is likely due to fine-scale population structure that is not controlled for by the inclusion of 25 common variant-based principal components; this fine-scale structure is likely correlated with variables used in the imputation and hence amplified when using imputed rather than observed FIS. As proxy for this fine-scale structure, we added the number of singleton synonymous variants exome-wide as a covariate, since individuals carrying an unusually high number of singletons relative to the sample may have subtly more distant ancestries than the majority of participants; we use synonymous variants as they are likely to be largely functionally neutral and hence not associated with the phenotype other than through population stratification. This rendered the association between observed average FIS and rare synonymous burden non-significant; it also reduced the association with imputed average FIS, while barely altering the negative association with rare PTV and damaging missense variants (Supplementary Figure 11), indicating that the latter is likely to be causal.

Sensitivity analyses controlling for the number of singleton synonymous variants

Given the concerns about subtle population stratification highlighted above in the analyses of exome-wide rare variant burden, we repeated the gene-based rare variant tests of average observed FIS and MegaFIS-average while controlling for the number of singleton synonymous per individual. We found this made very little difference to the significance of our associations (Supplementary Figure 13), suggesting that our gene discovery analyses are robust to any subtle stratification. This is presumably because we are only powered to find large effects which are most likely to be truly causal.

Comparison of our results with those of Chen et al.

At our FDR<1% threshold, we replicate all of the genes reported by Chen et al. as associated with FI (which they call 'verbal-numerical reasoning', VNR) at Bonferroni significance (*KDM5B*, *ANKRD12*, *RC3H2, CACNA1A*); of these, all passed this same p-value threshold ($p<4.2\times10^{-6}$) in our analysis using the PTV burden test on observed FIS - the closest comparison with Chen et al. - except for *CACNA1A* ($p=1.8\times10^{-3}$ on this test, although much more significant when considering combined FIS ($p=6.4\times10^{-6}$ on the PTV burden test; Supplementary Table 12). The additional five genes they reported at FDR<5% did not pass our FDR<1% threshold (*NDUFA6*, *ARHGEF7*, *C11orf94*, *KIF26A* and *MAP1A*).

Differences between our results and those of Chen et al. can likely be explained by several factors. They integrated the various FI measures in a different way to us: specifically, they considered the initial assessment visit, first repeat assessment visit and first imaging visit (FIS1, 2 and 3 in Supplementary Table 1) and took the highest measure of these. They also only considered unrelated individuals. Hence, their sample size for VNR was only N=128,812 unrelated Europeans, whereas ours was N=273,091 for observed FIS. Furthermore, they considered only burden tests on PTVs, whereas we also conducted tests incorporating missense variants, as well as SKAT-O tests. Finally, they used slightly different variant and genotype QC to us, and also presumably calculated their minor allele frequency for filtering on a different set of individuals. However, the most important difference between our studies is our incorporation of imputed FIS; this explains most of the increase in the number of significant genes.

Supplementary Note 8: Rare variant associations with FIS

Characterization of the FIS-prioritized genes

Fourteen genes passed FDR<1% in the rare variant association tests with FI and were also DDG2P genes, a 4.18-fold enrichment (Fisher's exact test $p=8\times10^{-8}$). This enrichment was also more pronounced than that observed for MAGMA-prioritized genes from the Combined FIS + COGENT GWAS (Extended Data Figure 4b). Among the twenty-six genes identified through rare variant testing, seven (*IGF1R*, *KCNMA1*, *GIGYF1*, *IPO9*, *CACNA1E*, *DPP8*, *NF1*) were also prioritized by gene-level enrichment for common variants in the Combined FIS + COGENT GWAS, but this overlap was not significant (Fisher's exact test p=0.09).

All of the fourteen DDG2P genes that we identified as associated with FI have been reported to cause syndromes with neurodevelopmental features, although in the case of *IGF1R* and *NF1*, these features are not always present[10,11]. Our findings expand our knowledge of the mechanism of action of several of these genes. Seven have been reported to show dominant inheritance with an altered-function mechanism (*CACNA1A*[12,13], *CACNA1E*[12,13], *ATP1A1*[14], *PPFIA3*[15], *GABRA2*[16], *KCNMA1*[17] and *SPTBN2*[18]), of which the latter two also show recessive inheritance with a LoF mechanism[19,20] Four of the fourteen DDG2P genes have previously only been reported to show dominant inheritance with a LoF mechanism (*TNRC68*[21], *GIGYF1*[22], *NF1*[23] and *MYCBP2*[24]) and one shows only recessive inheritance with a LoF mechanism (*BCAS3*[25]).wo of the fourteen genes (*IGF1R*[11] and *KDM5B*[26]) show both dominant and recessive inheritance with a LoF mechanism. This is an unusual property observed for only 31 of 2447 (1.2%) DDG2P genes to date; observing this in 2 of 14 DDG2P genes that passed FDR<1% here represents a significant enrichment (Fisher's exact test p=0.01; odds ratio=12.9). These findings support those of Fridman et al. who found that heterozygous carriers of pathogenic variants in recessive intellectual disability genes had, on average, lower educational attainment in UKB[27] and that selection against damaging variants in genes associated with autosomal-recessive disease is likely acting on heterozygotes[28]. They also suggest that there may be more DD-associated genes than currently appreciated that show a gradient of heterozygous effects rather than being clearly "dominant" or "recessive"[29].

Eight genes passing FDR<1% in the rare variant association tests with FI are neither DDG2P genes nor already reported by Chen *et al.*[30] (Table 1). Both mono- and biallelic disruption of *ANK3* has been reported to cause non-syndromic ID, with the biallelic form being apparently more severe[31]. Recurrent deletions in *ROBO2*, encoding a receptor expressed in the growth cones of extending axons, have been reported in autism[32], and this gene is also the most likely causal gene near a GWAS hit for expressive vocabulary in infancy[33] which is associated with later cognitive ability[34]. *TLK1* encodes a serine/threonine kinase in the same family as *TLK2*, a gene known to cause a neurodevelopmental condition [35]; *de novo* missense mutations in *TLK1* have been reported in two neurodevelopmental condition patients[36]. *CHD9* is in the same family of chromatin remodelers as *CHD7* and *CHD8*, both well-known neurodevelopmental condition-associated genes[37,38]; all three of these genes are in gene modules that are enriched for expression in the prenatal human brain[39]. Some *de novo* missense mutations in *CHD9* have been reported in autism[40–42]. Duplications in *RB1CC1* are associated with schizophrenia[43]. *PCLO* encodes a protein involved in synaptic transmission and is a GWAS hit for major depression[44] and post-traumatic stress disorder[45]. There is no prior genetic evidence directly linking *DPP8* and *IPO9* to neurodevelopment in humans, although *Dpp8*-deficient mice showed some behavioural abnormalities[46] and brain development was severely disrupted in *Ipo9*-deficient mice[47].

Neurodevelopmental conditions association of twelve FIS-associated non-DDG2P genes

Given the strong enrichment for DDG2P genes amongst our FIS-associated genes, we asked whether the twelve genes passing FDR<1% that were not DDG2P genes also showed direct evidence for association with neurodevelopmental conditions. Specifically, we tested whether they were enriched for *de novo* mutations amongst ~31,000 probands with neurodevelopmental conditions from three published cohorts[48]. We compared the observed

number of *de novo* mutations in these genes to the number expected to occur by chance alone, based on a statistical model that accounts for the background mutation rate at each genomic position. Across the twelve genes, we observed a significant enrichment of damaging *de novo* PTVs compared to the expectation under a null mutation model[49] (observed/expected = 2.59, p=6.8x10^{-4}, one-tailed Poisson test) but, reassuringly, no enrichment of *de novo* synonymous mutations (observed/expected = 1.08, p=0.40). This finding strongly suggests that damaging *de novo* variants in these FIS-prioritized genes elevate risk of neurodevelopmental conditions.

Rare variant gene associations in UKB after excluding individuals with neurodevelopmental conditions

Given many of the significant genes we find are known to cause neurodevelopmental conditions, we wondered whether their associations with FI in UKB were driven by individuals recorded to have these conditions. Of the 2,045 individuals with rare PTV or damaging missense variants driving the signals for the significant genes in Table 1, 303 (~15%) had healthcare records indicating intellectual disability, epilepsy, or autism. When we removed individuals with these codes and reran the rare variant tests, the p-values and effect sizes for most genes changed minimally (Extended Data Figure 5; Supplementary Table 13). All genes still passed multiple-testing correction except *CACNA1E* and *KCNMA1*, for which the p-values dropped from 1x10^{-6} to 6x10^{-4} and from 1x10^{-10} to 4x10^{-3} respectively. This suggests that rare damaging variants in most of these genes are associated with reduced FI even in individuals who do not have recorded diagnoses of neurodevelopmental conditions.

Replication in birth cohorts

We sought to replicate FIS-associated genes using recently-generated exome sequence data from two birth cohorts: ALSPAC (N=5283) and MCS (N=5621)[50]. Since we did not expect to have power to investigate any individual gene given the much smaller sample sizes of the replication cohorts, we collapsed the genes into three gene sets: the twenty-six genes passing FDR<1%, the eighteen of those that were not found by Chen et al. at FDR<5% with any of their cognitive phenotypes, and the twenty-one genes that passed FDR<1% in our analyses only after adding in the imputed FIS. Rare PTVs in all three of these gene sets showed significant negative effects on childhood cognition in both cohorts that became more significant after meta-analysis (Figure 3D). Thus, rare damaging variants in the novel genes discovered after incorporating imputed FI also associate with direct measures of cognitive ability.

Supplementary Note 9: Rare variant associations with educational attainment

Imputed FIS versus educational attainment

We explored whether analysing MegaFIS-average gave us more power for rare variant analyses than analysing educational attainment (EA) on essentially the same sample (N=434,146; slightly smaller N than for combined FIS due to missing EA values). We ran the same set of rare variant tests as described for FIS (Supplementary Table 14) and then calculated the FDR across all tests. We found nine EA-associated genes passing FDR<1%,

in contrast to the twenty-six that were found using average observed FIS or MegaFIS-average (Figure 3C). Of these nine genes, six were overlapping with our analysis of average observed FIS or MegaFIS-average (*ANKRD12*, *KDM5B*, *ADGRB2*, *BCAS3*, *TNC6B* and *GIGYF1*), but three were not (*FOXK2, CHD6* and *CADPS2;* all with $p<1x10^{-3}$ for all FIS tests) (Extended Data Figure 6). None of these three novel genes are known to be associated with neurodevelopmental conditions. However, *CHD6* is in the same family of chromatin remodelers as the aforementioned neurodevelopmental condition-associated genes *CHD7* and *CHD8*, and *FOXK2* encodes a transcription factor with roles in neural cell proliferation and neural precursor differentiation[51,52]. Notably, PTVs in *CADPS2* (calcium-dependent secretion activator 2) showed a *positive* association with EA (burden test; beta = 0.31, p-value = $2.5x10^{-6}$), in contrast to the other twenty-eight FDR-significant genes reported here, in which PTVs all showed *negative* effects on EA and/or FIS. PTVs in *CADPS2* showed no significant association with FIS (p>0.05 on all tests; for observed FIS, PTV burden beta=0.076; p=0.48). This gene also shows common variant associations with EA but not FIS, and colocalization with expression quantitative trait loci (eQTLs) in the brain suggests that decreased gene expression is associated with higher EA (see "Association between CADPS2 and educational attainment"), concordant with our rare variant findings.

In summary, incorporating imputed FIS boosted our power for rare variant-based gene discovery analyses over what could be achieved with EA alone. This may be due to several reasons, critically that EA may have a lower rare variant heritability than MegaFIS-average; certainly the twin-heritability (52% versus 67%)[53] and the common SNP heritability (14.3% versus 18.6%)[54] have been reported to be slightly lower for EA than cognitive performance. Notably, the genetic correlation estimated from direct effects between EA and cognitive ability is only 0.47[54], implying that these two phenotypes have considerably distinct genetic contributions. This supports our findings that the genes that show rare variant associations with EA only partially overlap with those for FI, including *CADPS2.*

Association between *CADPS2* and educational attainment

We found a significant positive association between rare PTVs in *CADPS2* and educational attainment (burden test beta = 0.31, p value = $2.5x10^{-6}$), but found no effect on FIS (p>0.05 on all tests). This finding is consistent with results attained by Chen et al., which also showed a suggestive positive association between PTVs in *CADPS2* and EA (beta = 0.25, p value = $4.9x10^{-4}$). The absence of impact on FIS provides molecular evidence that EA is genetically distinct from FIS, suggesting *CADPS2* may influence traits specifically related to educational persistence or achievement rather than cognitive ability per se. Consistent with these rare variant findings, *CADPS2* has been implicated in a MAGMA gene prioritization analysis of EA using a common variants GWAS[55] (FDR-adjusted p-value=$7.55x10^{-14}$), but we find no evidence for gene prioritization using MAGMA using any of our FIS GWASs (FDR adjusted p-value>0.05). Additionally, a conditional and joint (COJO) multi-SNP analysis of the largest GWAS of EA[56] identified two lead SNPs at this locus (rs6466819 and rs2430048, COJO p-value < 10^{-11} for both), both of which had p > 0.01 in our FIS GWASs as well as p > 0.6 in the GWAS for the cognitive component of EA[5]. Interestingly, rs6466819 showed a suggestive association (p=$1.5x10^{-4}$) with the non-cognitive component of EA [5], and other SNPs at this locus have been associated with risk taking[57] (rs10252114; p=5×10^{-11}) as well as with brain imaging traits[58] .

The rs6466819 GWAS hit for EA has been colocalized with an eQTL for *CADPS2* in cerebellum by OpenTargets[59]. This analysis found that variants associated with decreased expression of *CADPS2* were associated with increased EA. For example, the A allele at the lead eQTL at the locus, rs10241928, is associated with decreased *CADPS2* expression (eQTL beta = -0.28, p value = 1.7×10^{-6}) and with increased EA in Okbay et al. (GWAS beta = 0.00904, p value = 3.1×10^{-7}). The same conclusion was reached when using either the Okbay et al. or Lee et al. GWAS results. Coupled with our observation, this suggests we have identified a directionally concordant allelic series at *CADPS2* for EA.

CADPS2 is strongly expressed in various brain regions[60,61] has been implicated in autistic-like behavioural phenotypes in mice with heterozygous loss-of-function mutations [62]. Additionally, the gene's LOEUF score of 0.7 (gnomAD v4.1.0) indicates mild depletion of heterozygous pLoF carriers, consistent with subtle but not strong constraint against loss-of-function variation.

Owing to the novelty of this finding, we sought to replicate the association of EA and *CADPS2* using whole-genome sequencing data in the All of Us Research (AoU) program (AoU). We observed no significant association for rare PTVs in *CADPS2* and educational attainment in AoU (beta = 0.0060, p value = 0.14), although the direction of effect was positive (*cf* rare damaging missense *CADPS2* variants: beta = -0.0014, p value = 0.42). Several factors may explain the lack of replication. First, simple power differences from a smaller population cohort. Second, AoU yielded a greater number of rare PTVs (PTV carrier rate 0.047%, n=72 carriers) than in UKB (0.031%, n=138 carriers), due to the greater coverage at splice sites and intron-exon boundaries. A higher number of these predicted PTVs may be annotated incorrectly compared to UKB and reduce the specificity of the association analyses. Third, there was a large difference in the distribution of EA scores between cohorts, likely due to differences in recruitment which may have resulted in diminishing power to identify positive associations.

In summary, we found some evidence from both rare and common variants for a potential role for *CADPS2* in EA via traits other than cognition. The rare variant association needs to be confirmed in an independent dataset. If confirmed, it is notable for being the first gene (to our knowledge) in which rare loss-of-function variants are associated with increased rather than decreased EA.

Supplementary Tables

Supplementary Table 1

Overview of available FIS measures in UKB. Datafield 20016 is taken at the assessment centre via touch screen whereas 20191 is an online web-based questionnaire. We excluded the data collected at the first repeat imaging visit from our analyses due to the small sample size.

Supplementary Table 2

In both tables, the diagonal shows the correlation between raw values and transformed values (in which FIS was regressed on age and age2 then the intercept was added to the residual (see Methods) for each measure. Correlations for raw FIS measures are shown above the diagonal, and below the diagonal are the correlations for transformed FIS values. Standard errors are given in brackets. Note that the phenotypic correlations are effectively

the test-retest reliability, and are based on the subset of individuals who did both of the relevant tests (shown in Supplementary Figure 1). The genetic correlations are based on GWASs of each respective measure and therefore contain only partially overlapping sets of individuals.

Supplementary Table 3

Table showing LDSC calculated genetic correlations for Observed FIS1; Integrated GWASs Earliest observed FIS and Average observed FIS; Mega analyzed GWASs MegaFIS-vA, MegaFIS-vB, MegaFIS-earliest, MegaFIS-average, MegaFIS-all5; and intelligence reference GWASs by Savage et al. and the COGENT consortium.

Supplementary Table 4

Overview of UKB variables used for imputation in this study. Per variable the phenotypic correlation with FIS1 is shown, along with the genetic correlation with NonCogIFIS and Savage et al. Intelligence. The final selection of imputation variables covers a subset of all variables listed. Inclusion is shown in the last column.

Supplementary Table 5

Accuracy for the different imputation runs, and number of individuals used to assess this. Accuracy is computed as correlation between observed and imputed values for N individuals set to missing prior to imputation. Note that N differs per imputation run due to differing numbers of individuals amongst our static evaluation set of 50,000 randomly-chosen individuals having a measured value.

Supplementary Table 6

Common variant based comparison of combining imputed and observed measures. The top shows statistics of combining GWAS summary statistics via meta-analysis using METAL. The bottom shows statistics of combining via mega-analysis after first scaling imputed and measured values separately. Mega-analysis shows both higher SNP heritability and more identified lead SNPs using FUMA. Note that the sample sizes differ slightly between the various imputation runs due to how the outlier removal was done (Methods).

Supplementary Table 7

Here we compare downstream GWAS analyses of imputed and measured individuals separately within each imputation approach. Imputed average and imputed earliest FIS perform very similarly across all metrics. Although at first glance imputed All5 FIS appears to achieve the best imputed set, splitting this up (Supplementary Figure 5) shows the results are inflated by measured individuals

Supplementary Table 8

MAGMA results produced by the SNP2GENE tool in FUMA. The type column indicates the GWAS used as input. Per GWAS only FDR<1% significant protein-coding genes are shown.

Supplementary Table 9

Statistics of PGIs constructed with GWAS summary statistics from the various GWAS, tested in three independent cohorts. In ALSPAC we used full-scale IQ measured at age 8. In MCS we used a cognitive performance measure derived from a factor analysis using a combination of various cognitive tests administered across childhood. In INTERVAL we used the average of the two fluid intelligence tests administered 24 and 48 months after recruitment. The population effects indicate results from a regression of the PGIs on the cognitive ability measure, controlling for sex and 20 PCs. The direct effects were estimated in the two birth cohorts by additionally controlling for parental PGIs in the regressions.

Supplementary Table 10

Results from MAGMA gene-level tissue enrichment implemented in FUMA SNP2GENE. Per Intelligence measure -log10(P) values are shown for each GTEx v8 Tissue.

Supplementary Table 11

Regenie results for observed FIS (i.e. average measured FIS) and combined FIS (i.e. MegaFIS-average), for all tested genes. The column headers indicate the phenotype tested in the first row, then the variant consequence included in the second row (PTV, damaging missense (MIS) or PTV+MISS). N_carrier: number of rare variant carriers; BETA, SE, p are the effect size, standard error and p-value from the burden test; P_SKATO is the p-value from the SKAT-O test.

Supplementary Table 12

Regenie results for observed FIS (i.e. average measured FIS) and combined FIS (i.e. MegaFIS-average), for genes passing FDR<1%. (This is a subset of Supplementary Table 11, provided for convenience). The column headers indicate the phenotype tested in the first row, then the variant consequence included in the second row (PTV, damaging missense (MIS) or PTV+MISS). N_carrier: number of rare variant carriers; BETA, SE, p are the effect size, standard error and p-value from the burden test; P_SKATO is the p-value from the SKAT-O test.

Supplementary Table 13

Regenie results for observed FIS (i.e. average measured FIS) and combined FIS (i.e. MegaFIS-average), for genes passing FDR<1% after excluding individuals with ICD10 codes for intellectual disability, epilepsy or autism. The column headers indicate the phenotype tested in the first row, then the variant consequence included in the second row (PTV, damaging missense (MIS) or PTV+MISS). N_carrier: number of rare variant carriers; BETA, SE, p are the effect size, standard error and p-value from the burden test; P_SKATO is the p-value from the SKAT-O test.

Supplementary Table 14

Regenie results for educational attainment. The column headers in row 4 indicate the variant consequence included (PTV, damaging missense (MIS) or PTV+MISS). BETA, SE, p are the effect size, standard error and p-value from the burden test; P_SKATO is the p-value from the SKAT-O test.

Supplementary Figures

Supplementary Figure 1

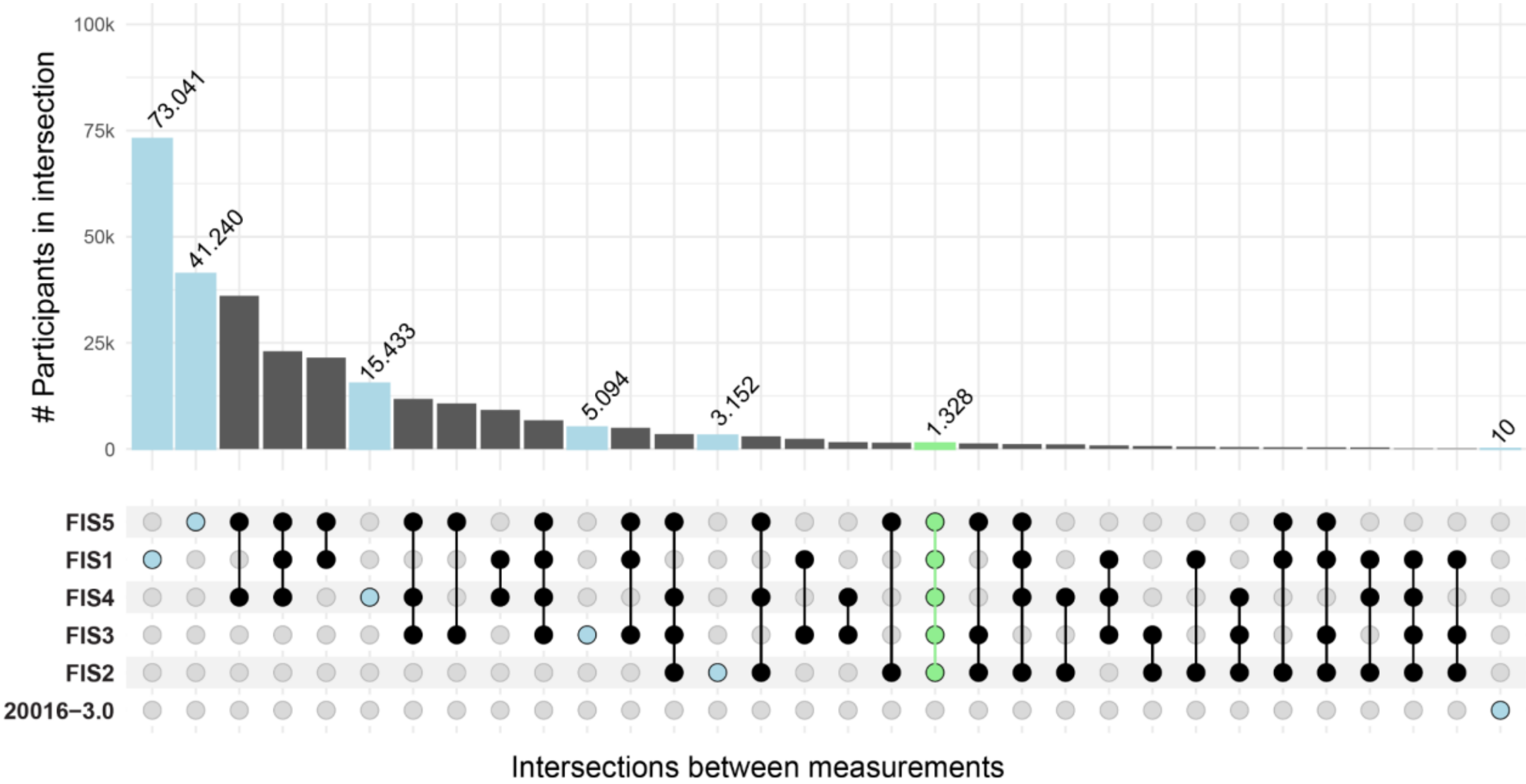

***Intersections between available FIS measurements*.** Highlighted in blue: Set of participants who only have the indicated measure; Highlighted in green: Intersection of participants measured at all FIS measures. Most participants have a FIS measure either at 20016-0.0 (FIS1) or one of the online FIS measures 20191-0.0 (FIS4), 20191-1.0 (FIS5), or a combination of those. 10 people were measured only during measurement 20016-3.0, which is why we omitted this measure in downstream analyses.

Supplementary Figure 2

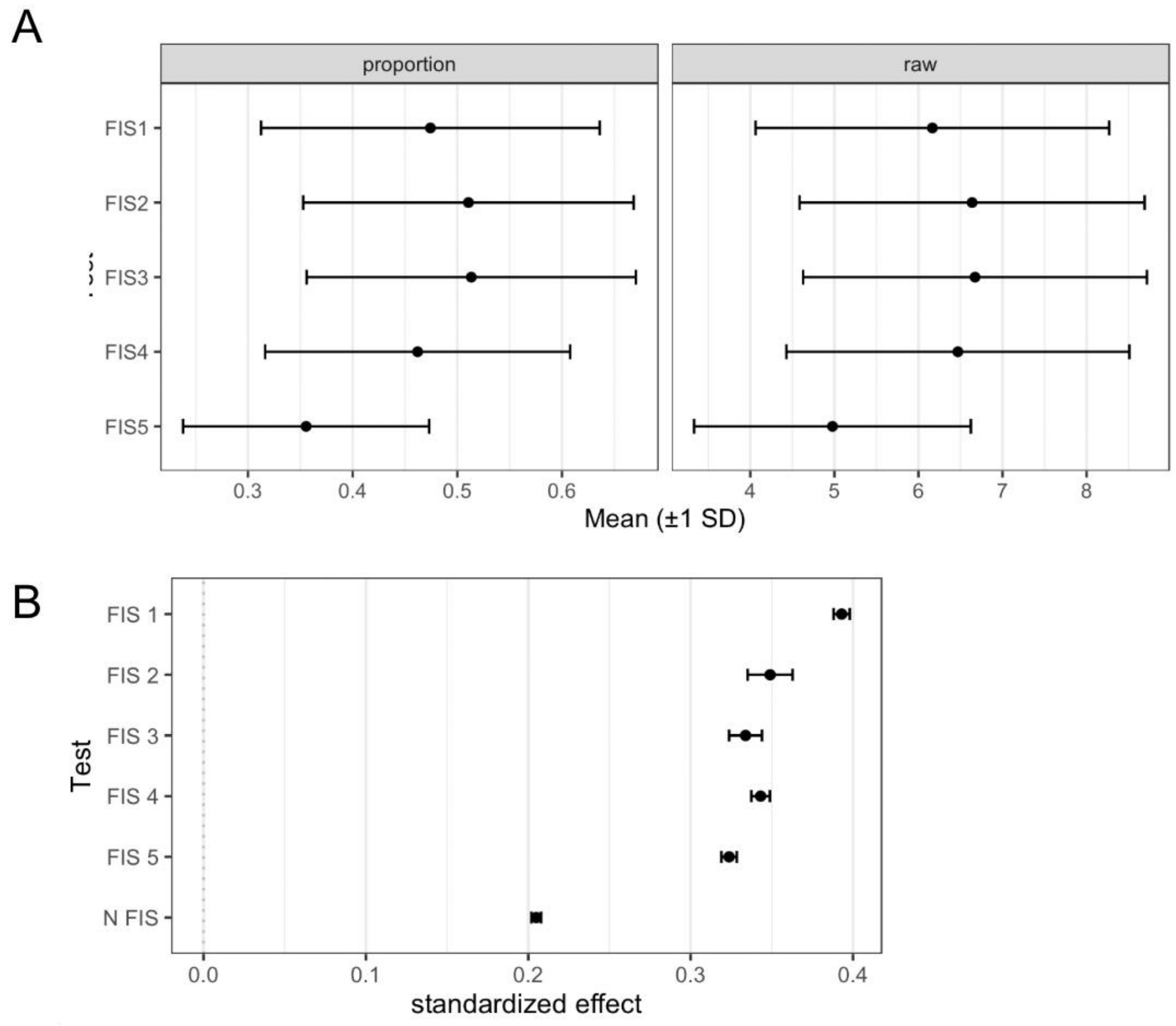

Average scores on the various FIS tests and association between FI test completion and number of FIS measures taken with EA. **A)** Mean and 1 standard deviation bands of FIS measures divided by total number of questions asked (proportion) and of the unscaled scores (raw). **B)** Associations between educational attainment (EA) and having a measured value for a given FIS measure and the total number of FI measures recorded using logistic and linear regressions, respectively. Error bars are 95% confidence intervals of the regression coefficients.

Supplementary Figure 3

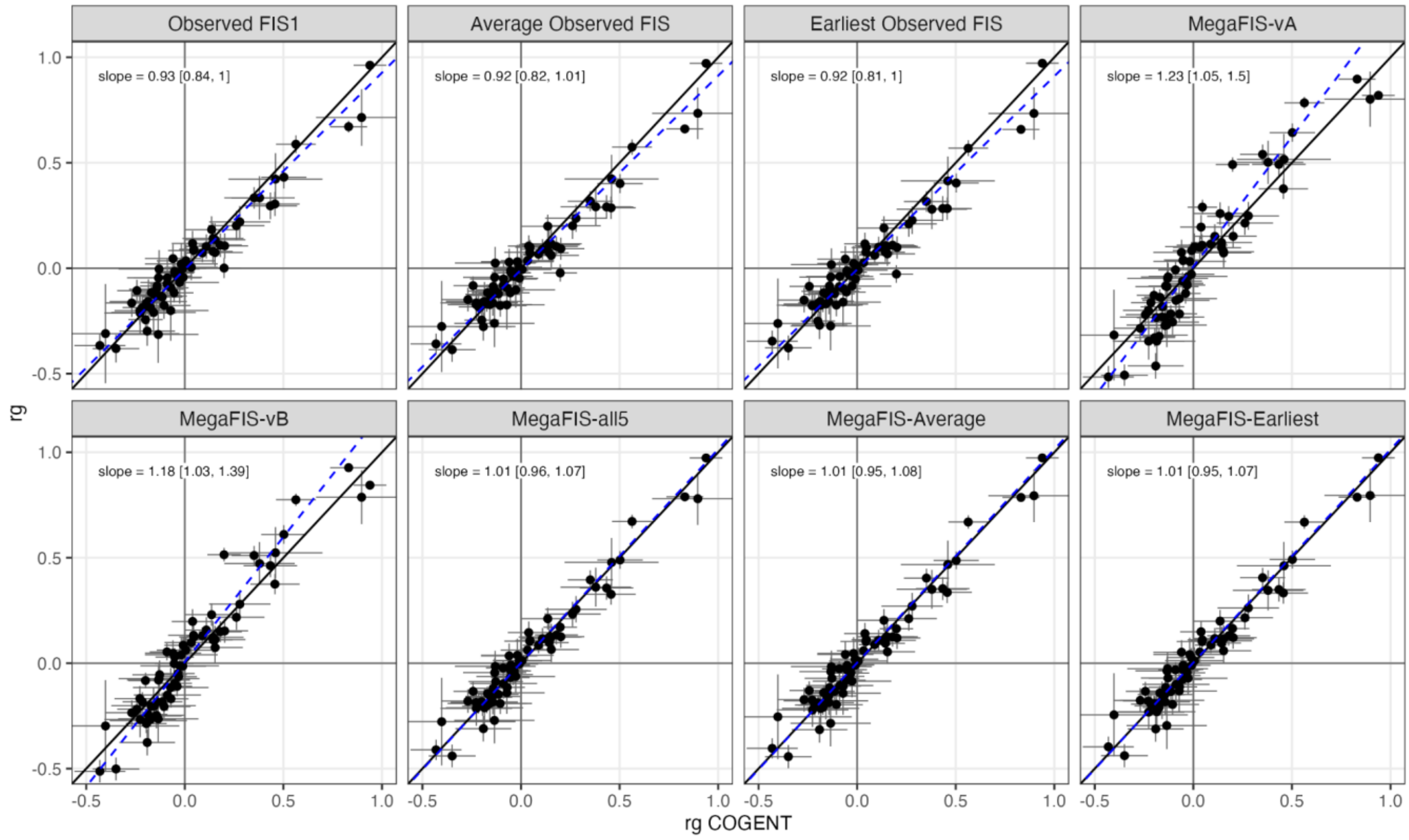

Genetic correlation profile comparison between derived FIS GWAS and COGENT. Genetic correlations are estimated between derived FIS GWASs and 68 UKB phenotypes and plotted against genetic correlations estimated between COGENT and the same 68 UKB phenotypes. The black line indicates the y=x line and the dashed blue line indicates the Deming regression line of best fit, which takes into account the fact that genetic correlations are estimated with error. Error bars are 95% confidence intervals of the point estimates

Supplementary Figure 4

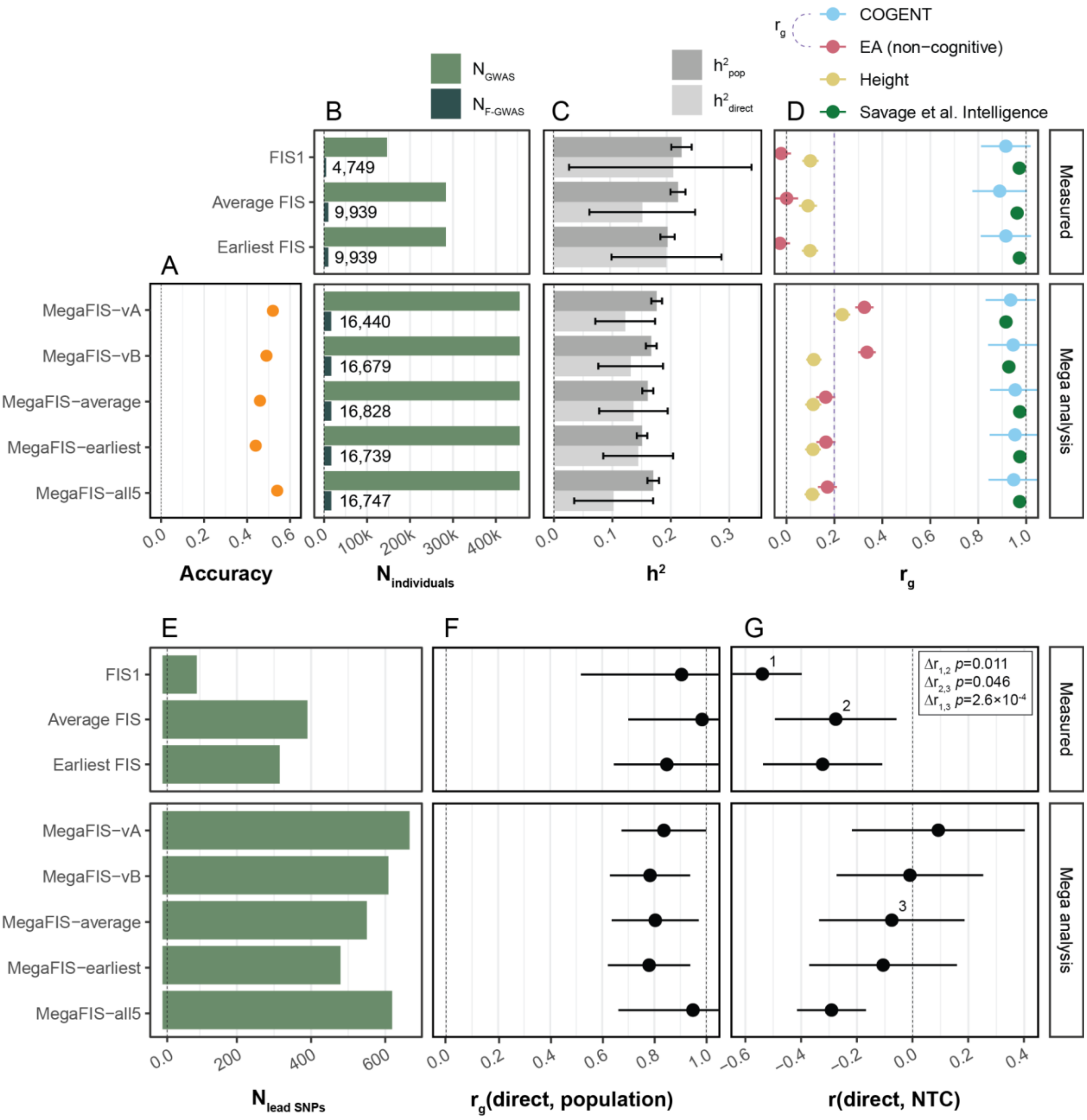

Evaluation of different data integration and imputation approaches. This is an extended version of Figure 1, adding statistics on MegaFIS-vA, MegaFIS-vB, MegaFIS-earliest and MegaFIS-all5. All panels give various summary statistics from either population-based or within-family GWASs of measured FI phenotypes (top three results) and our via mega-analyses combined GWAS (bottom result). **A)** Imputation accuracies of the imputed variables in each GWAS **B)** Sample sizes of population GWASs (N_{GWAS}) and within-family GWASs ($N_{F\text{-}GWAS}$); **C)** Population and direct effect heritabilities estimated using LDSC[63]; **D)** Population effect genetic correlations estimated using LDSC[64] with traits from external studies of intelligence (blue and green), the non-cognitive component of educational attainment (NonCogEA) (red), and height (yellow). The purple dashed line indicates the genetic correlation between the COGENT GWAS and NonCogEA; **E)** Number of significant ($p<5x10^{-8}$) independent lead SNPs from the population-based GWAS computed using FUMA[65]. Association was tested using linear regression, with two-sided tests of association. **F)** Genome-wide correlations estimated using LDSC between the population effects and the direct effects from a within-family GWAS conducted on the indicated trait; **G)** Correlations between direct effects and non-transmitted coefficients (NTC) estimated using snipar[66] for each corresponding trait..Inset p-values indicate the significance of two-sided z-tests for pairwise differences between estimates. Standard errors were obtained from a block jackknife procedure. P values were not adjusted for multiple comparisons. Panels b,c,e and f display point estimates ± 95% confidence interval (estimate ± 1.96 × s.e.).

Supplementary Figure 5

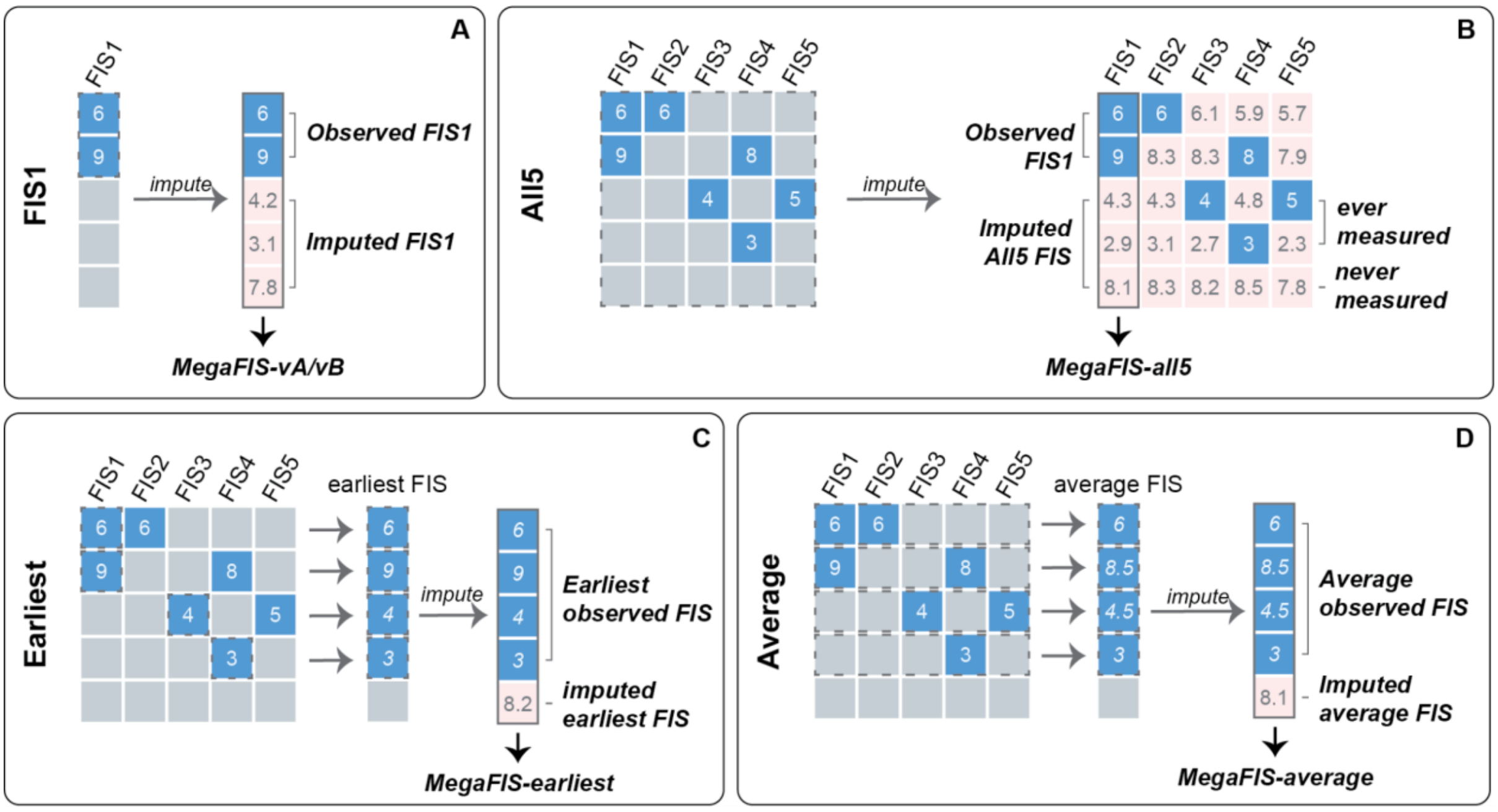

Schematic illustrating the various strategies for integration and imputation of fluid intelligence. (This is an expanded version of Extended Data Figure 1.) **A)** Missing values (grey) are imputed (red) using observed (blue) FIS1 and selected imputation variables. **B)** Five FIS are separately used in imputation along with selected imputation variables. Only imputed values for FIS1 are used in downstream analyses. **C)** Five FIS are integrated by taking the earliest available measure, which is then used for imputation along with selected imputation variables. **D)** Our selected approach: five FIS are integrated by taking the average of available measures, which is then used for imputation along with selected imputation variables. Observed and Imputed values are combined via mega-analysis as described in Supplementary Notes 2 and 3 respectively. Although raw FIS scores are shown for clarity, actual scores were transformed prior to imputation (see Methods).

Supplementary Figure 6

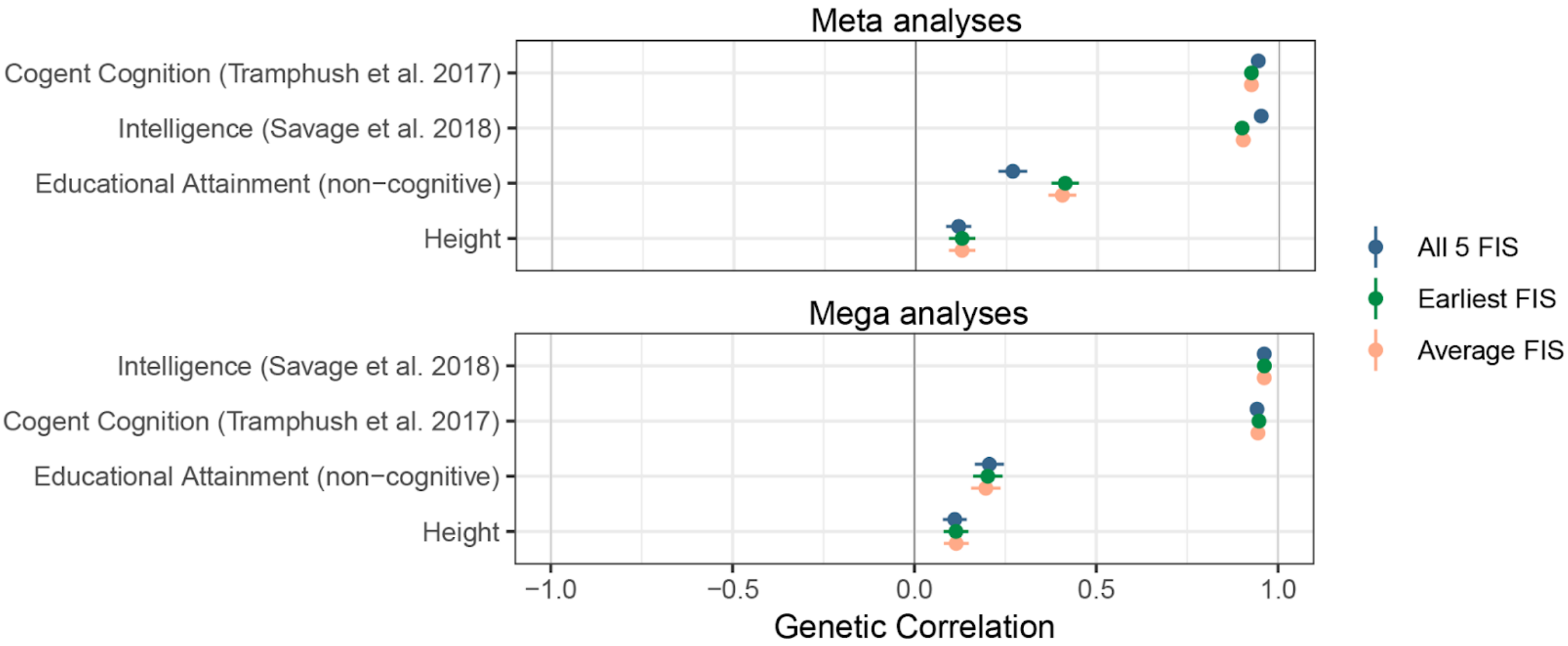

Genetic correlation for combined GWASs. Top panel: imputed and measured values were combined by meta-analysis for each of the three imputation approaches. Bottom panel: Imputed and measured values were scaled separately and combined in mega-analyses for each of the three imputation approaches.Error bars indicate the 95% confidence intervals of the point estimates (estimate ± 1.96 × s.e.)

Supplementary Figure 7

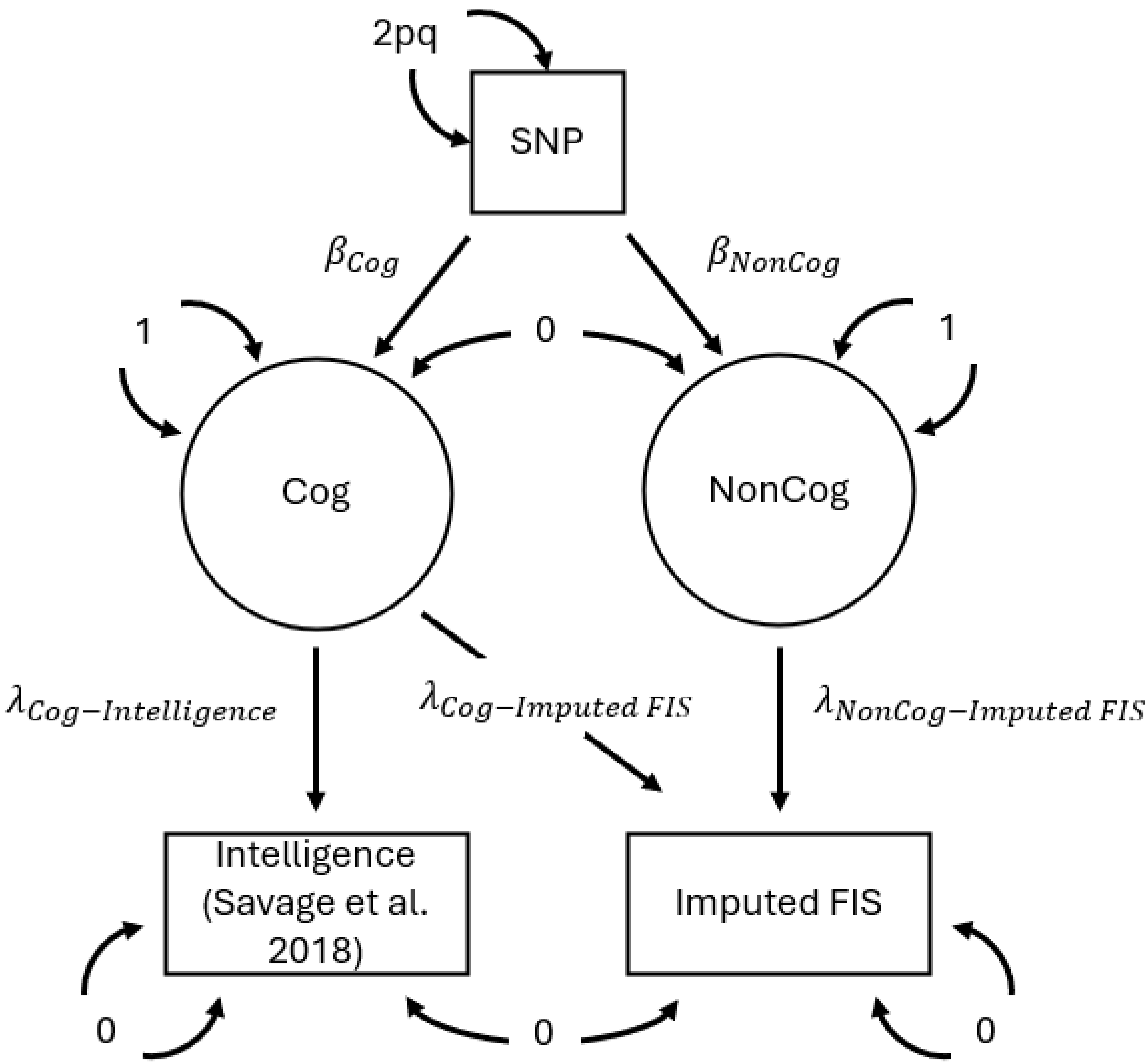

GWAS-by-subtraction model used to derive non-cognitive imputed FIS (NonCogIFIS). Boxes indicate observed variables from GWAS, circles represent latent factors for cognitive (Cog) and non-cognitive (NonCog) signals. Bidirectional arrows represent variances for the respective variables. The variance of SNP is defined as 2pq, representing genetic variance under Hardy-Weinberg equilibrium. Latent factor variances are fixed to 1 and observed variable variances and covariances are fixed to 0. This is to ensure that all variance of the model is explained by the defined latent factors. β parameters indicate effect sizes of a SNP on respective latent factors, λ parameters indicate path loadings obtained from regressing observed variables on the latent variables. Genetic signals that are shared between GWASs of Intelligence and imputed FIS (first version, 154 imputation variables) are assigned to latent factor Cog, the residual signal in imputed FIS is assigned to NonCog.

Supplementary Figure 8

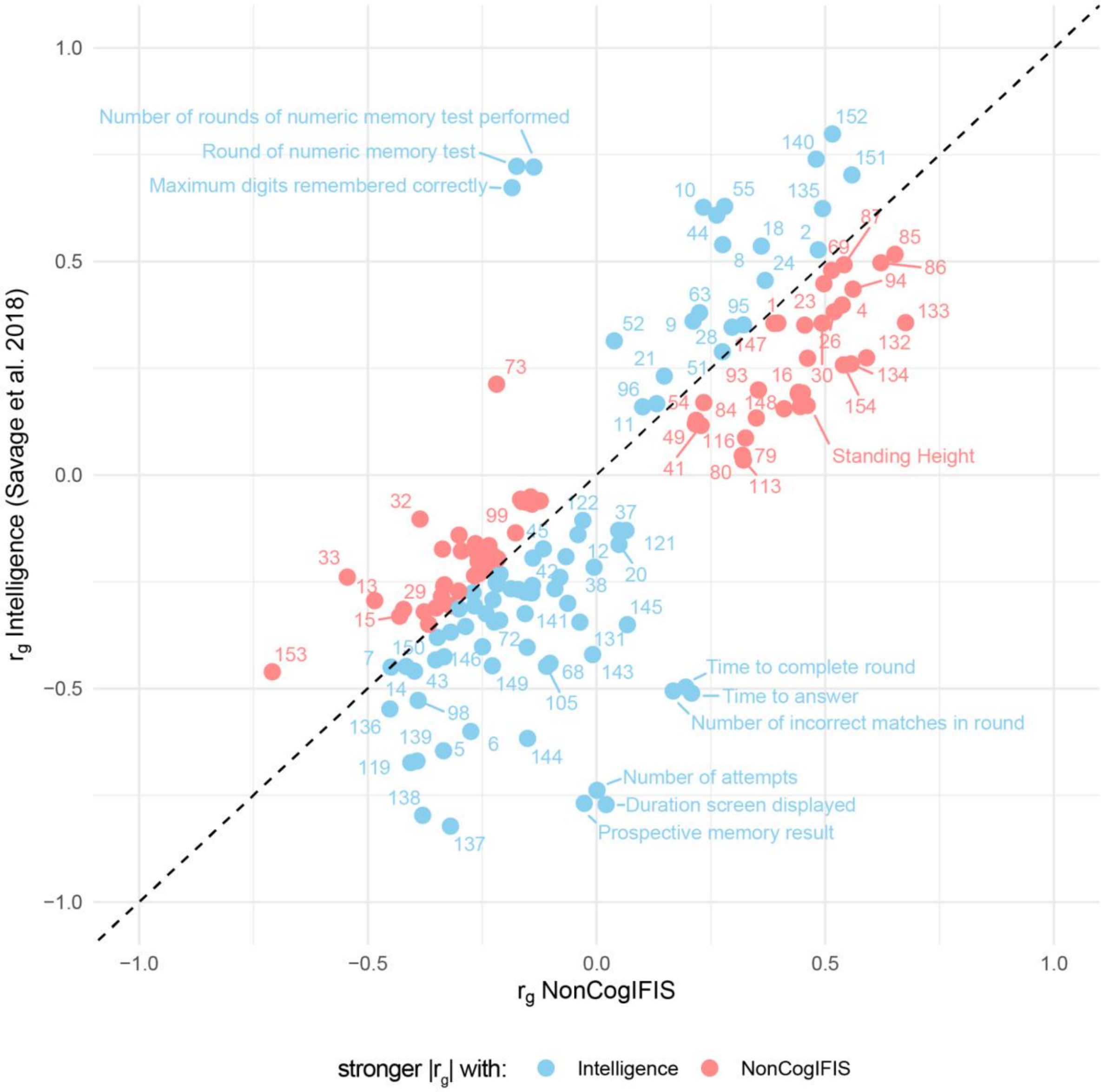

Correlation based imputation phenotype selection. This table shows the genetic correlation of each trait used in imputation with measured intelligence (Savage et al.) and NonCogIFIS. NonCogIFIS contains the genetic signal of the imputed FIS GWAS (first version, 154 imputation variables) that is not captured by the measured intelligence GWAS by Savage et al. *red dots |rg(NonCogFIS)| > |rg(Intelligence)|; blue dots |rg(Intelligence)| > |rg(noncogFIS)|. Labels correspond with the labels in Supplementary Table 2.*

Supplementary Figure 9

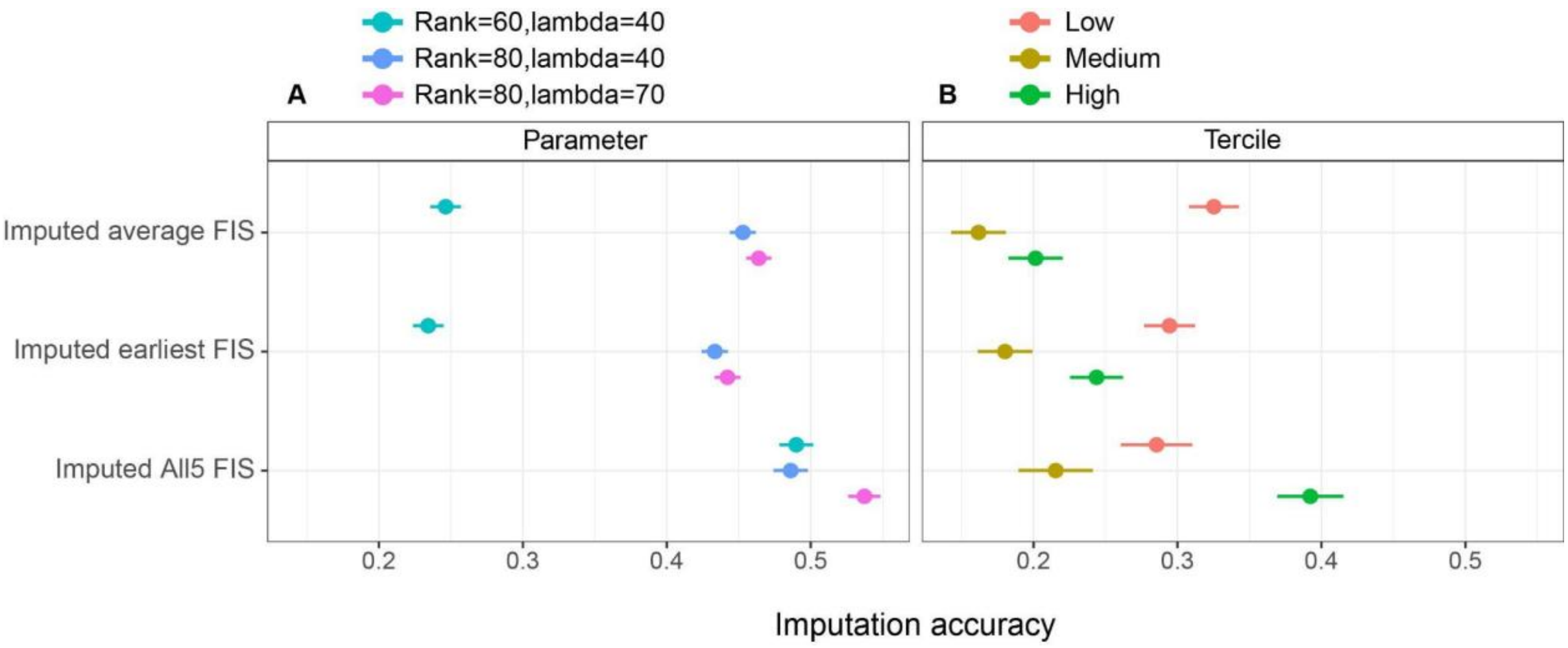

Phenotypic accuracies across FIS imputation approaches. **A)** Shows the overall accuracy when using different imputation parameters. **B)** Shows the accuracy obtained with rank=80 and lambda=70 for three tranches of people divided by their respective observed FIS value (observed average FIS observed earliest FIS, FIS1). In both panels error bars indicate 95% confidence intervals.

Supplementary Figure 10

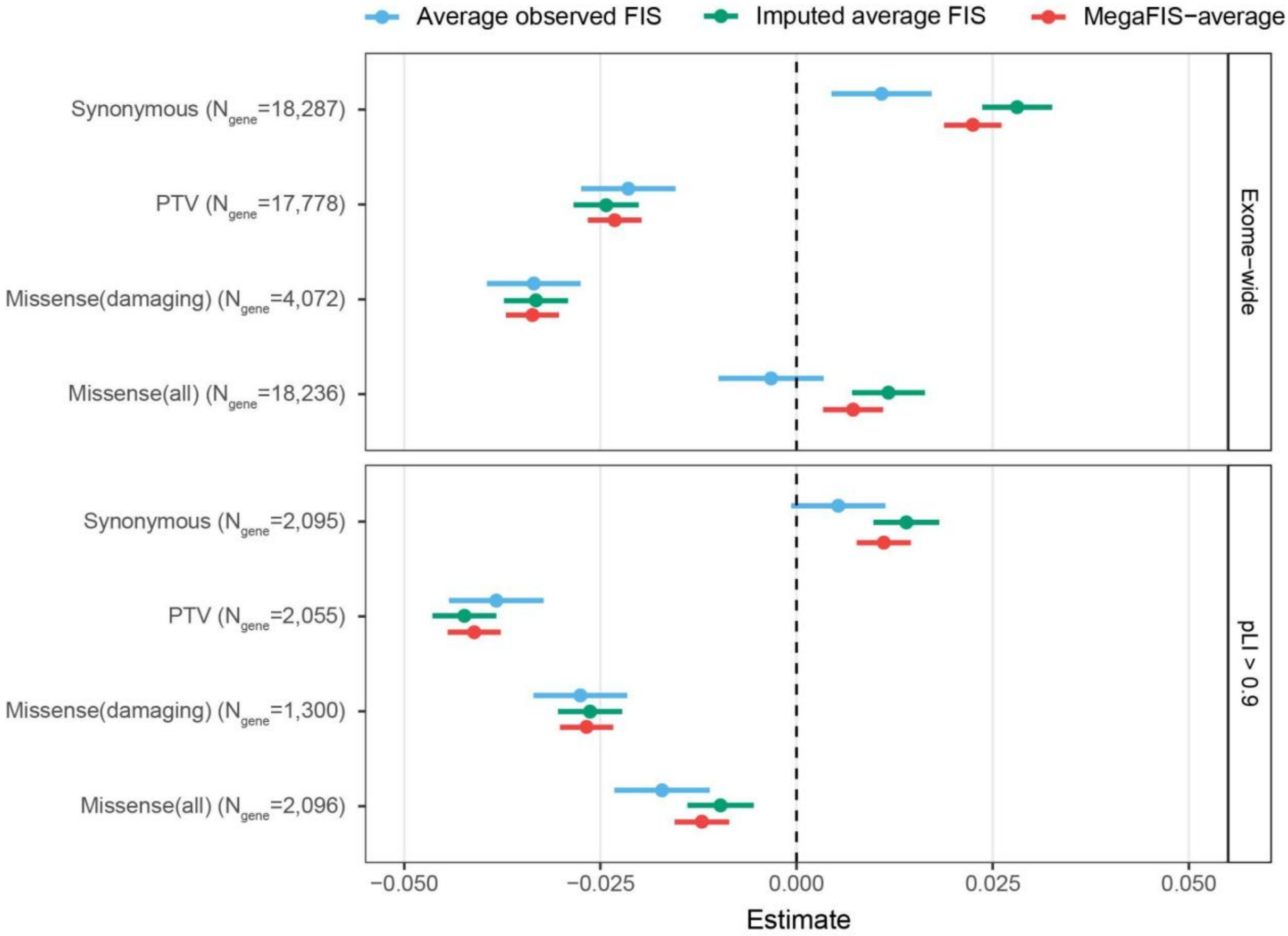

Impact of exome-wide burden of rare protein-coding variants for FIS in EUR samples in UK Biobank. The effects of protein-truncating, all missense, damaging missense and synonymous variant burden on Average observed FIS (blue), imputed Average FIS (green), and MegaFIS-average (red) across the exome (top) and in genes with high LOF intolerance (pLI ≥ 0.9) (bottom). Note that these three subsets are referred to as “observed FIS”, “imputed FIS” and “combined FIS” respectively in the main text. Unrelated UKB EUR samples were included in this analysis (n = 328,795). pLI is the probability of being LOF-intolerant as recorded in the gnomAD database. The number of genes included in each burden was labelled. Data are presented as effect size estimates (β) with 95% confidence intervals.

Supplementary Figure 11

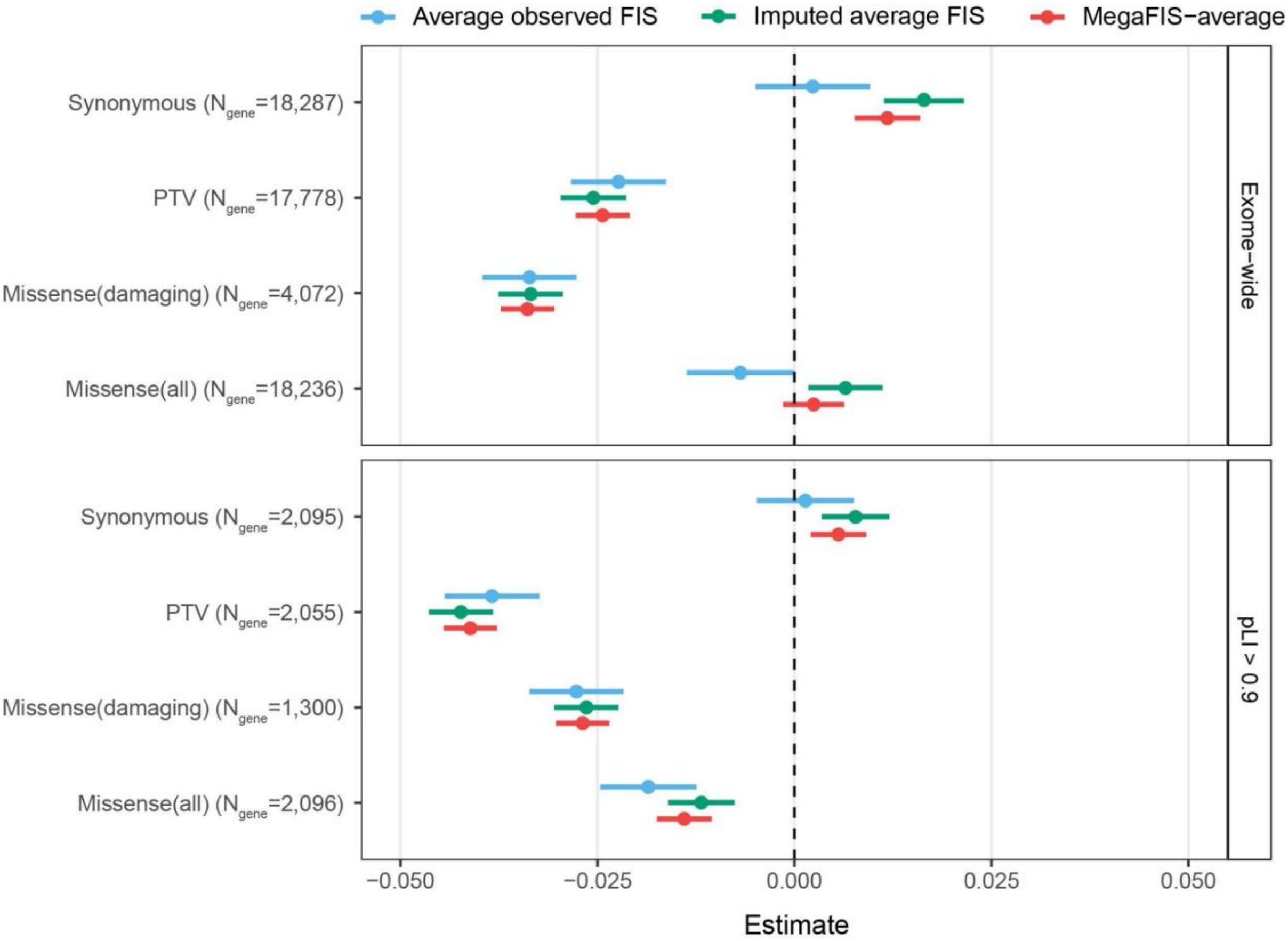

Exome-wide burden of rare protein-coding variants for FIS in EUR samples in the UKB controlling for the number of singleton synonymous variants per individual. The effects of protein-truncating, all missense, damaging missense and synonymous variant burden on average observed FIS (blue), imputed average FIS (green), and MegaFIS-average (red) across the exome (top) and in genes with high LOF intolerance (pLI ≥ 0.9) (bottom). Note that these three subsets are referred to as “observed FIS”, “imputed FIS” and “combined FIS” respectively in the main text. Unrelated UKB EUR samples were included in this analysis (N = 328,795). pLI is the probability of being LOF-intolerant as recorded in the gnomAD database. The number of genes included in each burden was labeled. Data are presented as effect size estimates (β) with 95% confidence intervals.

Supplementary Figure 12

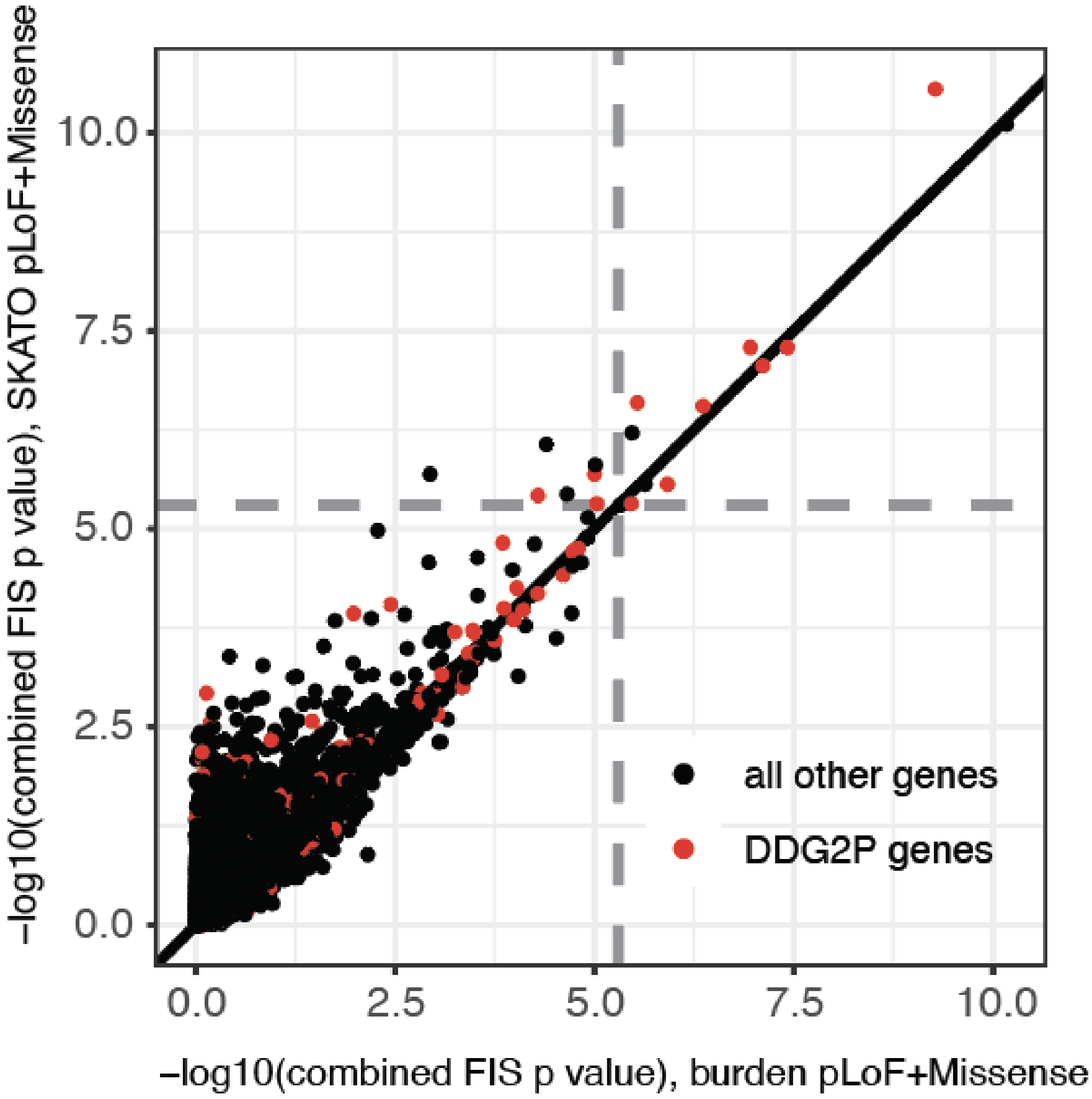

Results from gene burden test versus SKAT-O. -log10(p-values) obtained with SKAT-O versus burden tests, using PTV+damaging missense variants, for combined FIS (two-sided t-test for burden associations, non-directional omnibus test for SKAT-O). Gray dashed lines indicate the empirical p value cutoff for an FDR <1% in our gene association tests ($p<5.03\times10^{-6}$).

Supplementary Figure 13

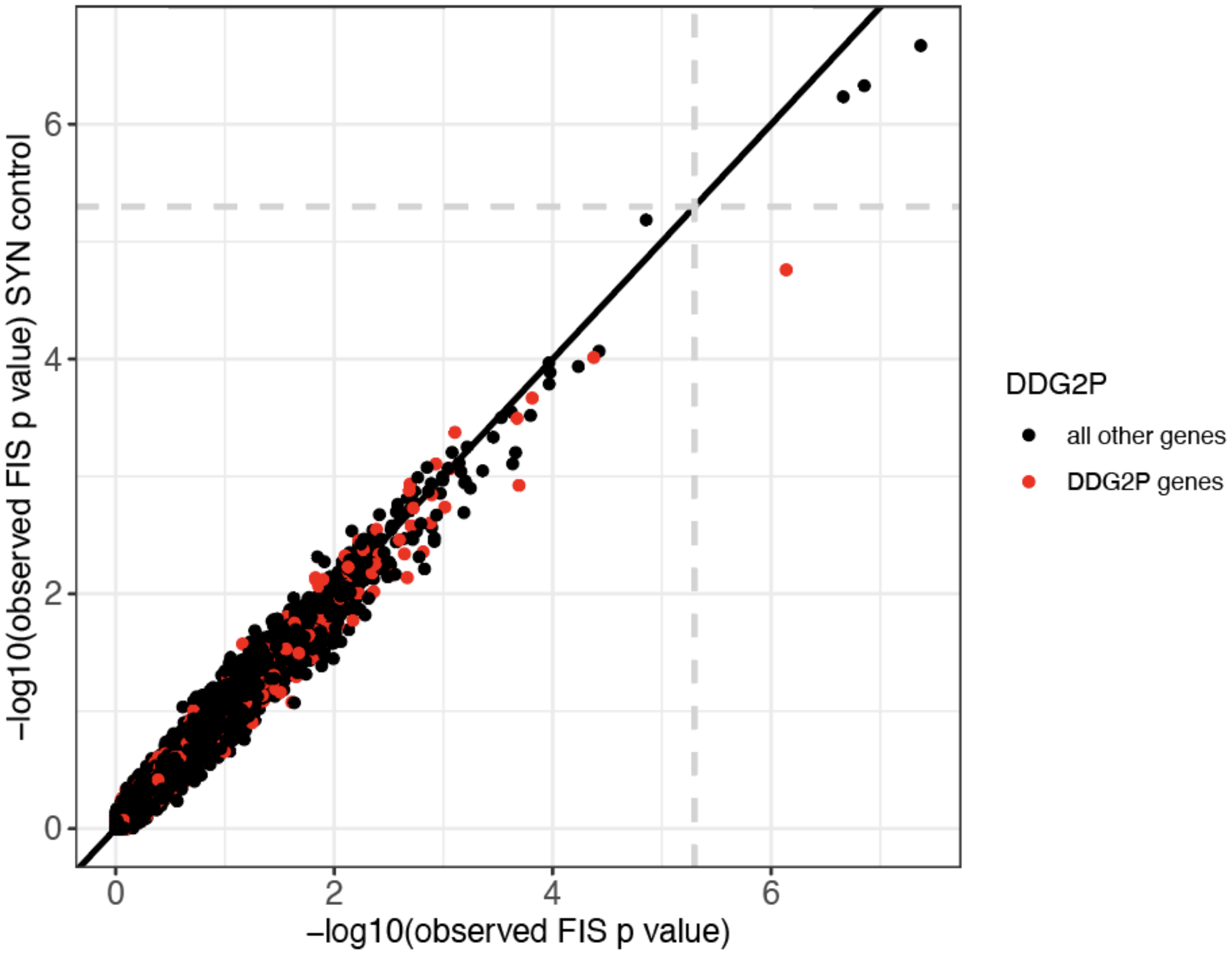

Results from gene-based rare variant burden tests before and after conditioning on the number of singleton synonymous variants. On the x-axis is the -log10(p) for the PTV burden test and on the y-axis is the -log10(p) after conditioning on the number of singleton synonymous variants an individual carries using observed FIS (two-sided t-test). Gray dashed lines indicate the empirical p value cutoff for an FDR <1% in our gene association tests ($p<5.03\times10^{-6}$).

Supplementary Methods

Introduction to the replication cohorts

The Avon Longitudinal Study of Parents and Children (ALSPAC)[7,67], and the Millennium Cohort Study (MCS) are two British birth cohorts that followed a group of participants from their birth. The studies collected information on education and employment, family and parenting, physical and mental health, and social attitudes, as well as applying cognitive tests at various ages. ALSPAC recruited 14,833 women based in Avon, UK who were pregnant during 1991 and 1992 and eventually included a total of 14,901 children of predominantly white ethnicity (>95%). In addition, 12,113 partners of pregnant women also participated in the study and 3,807 are still enrolled. Please note that the study website contains details of all the data that is available through a fully searchable data dictionary and variable search tool.[68] Ethical approval for the study was obtained from the ALSPAC Ethics and Law Committee and the Local Research Ethics Committees. MCS is a UK-wide nationally representative cohort that recruited 18,818 children born between 2000 and 2002. It intentionally over-sampled areas with high child poverty, large ethnic minority populations, and smaller UK nations (Wales, Scotland and Northern Ireland). INTERVAL is a prospective cohort study of about 50,000 participants nested within a pragmatic randomized trial of blood donors [69]. Between 2012 and 2014, blood donors aged 18 years and older were recruited from 25 National Health Service Blood and Transplant (NHSBT) donor centers across England. All participants gave informed consent before joining the study and the National Research Ethics Service approved this study (11/EE/0538). Participants were predominantly healthy individuals since people with major disease (myocardial infarction, stroke, cancer etc.) are excluded from blood donation, as well as those with recent illness or infection. Participants completed online questionnaires about basic lifestyle and health-related information, including self-reported height and weight, ethnicity, current smoking status, alcohol consumption, doctor-diagnosed anemia, use of medications (hormone replacement therapy, iron supplements) and menopausal status.

Quality control and imputation of ALSPAC and MCS genotype data

Quality control procedures included excluding individuals with high missing genotype rates (>3%) and filtering autosomal SNPs to retain those with MAF >0.5%, missingness <3%, and Hardy–Weinberg equilibrium $p > 1 \times 10^{-5}$. Relatedness was inferred using KING[70], and a set of unrelated individuals was derived by iteratively removing participants with excessive inferred kinship. European ancestry was confirmed by projecting samples onto 1,000 Genomes Phase 3 data using the smartpca function from EIGENSOFT, with variants pruned for linkage disequilibrium (pairwise $r^2 < 0.2$ in sliding windows) and exclusion of high/long-range LD regions (including the HLA). Principal component analysis was then conducted on the unrelated samples, and all individuals were projected into this space. Prior to imputation, SNPs that were palindromic, absent from the reference panel, or exhibited allele mismatches were removed. Genotypes were subsequently imputed to the TOPMed r2 reference panel via the TOPMed imputation server[71], and only well-imputed common variants (Minimac4 $R^2 > 0.8$; MAF >1%) were retained for analysis.

Quality control of ALSPAC and MCS whole-exome sequencing data

For both ALSPAC and MCS cohorts, the quality control of whole exome sequencing data was described in a previous paper[72]. First, we used GATK v4.2 to call short variants (SNVs and indels) in 11,994 samples from ALSPAC and 11,916 samples from MCS. We then applied sample quality control (QC) measures to remove outliers on several metrics (e.g., heterozygosity, variant counts) and likely sample mismatches. To identify low-quality variants (variant QC), we trained a random forest on pre-defined truth sets in each cohort separately and then used the random forest filtering in combination with genotype-level and missingness filters to balance precision, recall, true and false positive rates, and synonymous transmission ratios. Specifically, we filtered SNVs (genotypes set to missing) if they had an allele depth (DP) < 5, a heterozygous allele balance ratio (AB) < 0.2, or a genotype quality (GQ) < 20 (ALSPAC) or < 15 (MCS) and Indels using these thresholds: DP < 10, AB < 0.3, GQ <10 (ALSPAC) or GQ < 20 (MCS). We finally excluded the variants that failed the random forest filtering or if the fraction of missing genotypes (missingness) exceeded 0.5. In summary, we retained 8,436 children and 3,215 parents in ALSPAC and 7,667 children and 6,925 parents in MCS.

Quality control and imputation of INTERVAL genotype data

The genotyping and imputation protocol and QC for the INTERVAL samples (n ≈ 50,000) have been described previously in detail[73]. INTERVAL samples were genotyped in ten batches using DNA extracted from buffy coat on the Affymetrix Axiom UK Biobank array, which assayed approximately 830,000 variants. High-quality autosomal variants (MAF >0.05, HWE $p > 1\times10^{-6}$, and low inter-variant LD with $r^2 \leq 0.2$) were employed to identify duplicate samples (using a PLINK Method-of-Moments IBD threshold of 0.9) and to exclude non-European individuals based on principal component analysis (PCA), where subjects with PC1 or PC2 scores <0 relative to 1000 Genomes major ancestry populations were removed.

Subsequent variant-level filtering within each batch excluded variants that deviated strongly from Hardy–Weinberg equilibrium ($p < 5\times10^{-6}$) and those with a call rate <97%. Variants failing in multiple batches were dropped from the merged dataset. Sample contamination was assessed using the relationship between allele frequency and probe intensity, and samples with >10% contamination, or 3–10% contamination coupled with high relatedness, were removed. An additional set of ∼100,000 high-quality variants was used for global IBD analysis (to remove across-batch duplicates and overlapping participants with UK Biobank/UK BiLEVE) and for PCA using flashpca. Outlier individuals were excluded based on lower principal components, and the final PCA was rerun to generate covariates for downstream analyses, resulting in 43,059 participants.

Prior to imputation, further QC steps established a high-quality imputation scaffold by retaining autosomal, bi-allelic, non-monomorphic variants with a global HWE filter ($p > 5\times10^{-6}$), a within-batch call rate >99%, and a global call rate >75%. Non-autosomal, multi-allelic, and inconsistent variants were removed. Phasing was performed with SHAPEIT3 using chunks of 5,000 variants (with a 250-variant overlap), and imputation was conducted on the Sanger Imputation Server using the PBWT algorithm with a combined 1000 Genomes Phase 3–

UK10K reference panel. This process yielded approximately 87.7 million imputed variants. Imputation accuracy was subsequently validated against whole-exome sequencing data from 3,976 participants, confirming high levels of non-reference concordance and precision across common, low-frequency, and rare variants.

Calculating PGIs in INTERVAL

After removing 1,557 individuals who also participated in UKB and after removing one of each related pair (up to 3rd degree), 40,813 individuals remained. PGIs were calculated from the imputed genotype data in PLINK v1.9 using 967,141 variants, after filtering for imputation quality (INFO) score <0.8, minor allele count <8 and HWE p-value $< 5 \times 10^{-6}$, similar to Sun et al.[74].

Attempting replication of the rare variant association in CADPS2 with EA in All of Us

We sought to replicate the association of EA and *CADPS2* in the All of Us Research program[75] (AoU). Whole-genome sequencing data and relevant survey information were available for 152,378 participants of European ancestry. Gene-burden tests were performed with SAIGE-GENE+(Zhou et al. 2022), adjusting for sex, age, age², sex-by-age interaction, sex-by-age² interaction and the top 20 principal components of ancestry. EA was coded in accordance with Okbay et al.[56] based on responses to the question, “What is the highest grade or year of school you completed?” as follows: “Never Attended” as 0, “One Through Four” as 2.5, “Five Through Eight” as 6.5, “Nine Through Eleven” as 10, “Twelve Or GED” as 12, “College One to Three” as 14, “College Graduate” as 16 and “Advanced Degree” as 20.

References

1. Trampush, J. W. *et al.* GWAS meta-analysis reveals novel loci and genetic correlates for general cognitive function: a report from the COGENT consortium. *Mol. Psychiatry* **22**, 336–345 (2017).
2. Savage, J. E. *et al.* Genome-wide association meta-analysis in 269,867 individuals identifies new genetic and functional links to intelligence. *Nat. Genet.* **50**, 912–919 (2018).
3. Fawns-Ritchie, C. & Deary, I. J. Reliability and validity of the UK Biobank cognitive tests. *PLoS One* **15**, e0231627 (2020).
4. Brown, H. S. & May, A. E. A test–retest reliability study of the Wechsler Adult Intelligence Scale. *J. Consult. Clin. Psychol.* **47**, 601–602 (1979).
5. Demange, P. A. *et al.* Investigating the genetic architecture of noncognitive skills using GWAS-by-subtraction. *Nat. Genet.* **53**, 35–44 (2021).
6. Willer, C. J., Li, Y. & Abecasis, G. R. METAL: fast and efficient meta-analysis of genomewide association scans. *Bioinformatics* **26**, 2190–2191 (2010).
7. Fraser, A. *et al.* Cohort Profile: the Avon Longitudinal Study of Parents and Children: ALSPAC mothers cohort. *Int. J. Epidemiol.* **42**, 97–110 (2013).
8. Boyd, A. *et al.* Cohort Profile: The 'Children of the 90s'; the index offspring of The Avon Longitudinal Study of Parents and Children (ALSPAC). *International Journal of Epidemiology* **42**, 111–127 (2013).
9. Di Angelantonio, E. *et al.* Efficiency and safety of varying the frequency of whole blood donation (INTERVAL): a randomised trial of 45 000 donors. *Lancet* **390**, 2360–2371 (2017).
10. Crow, A. J. D. *et al.* A systematic review and meta-analysis of intellectual, neuropsychological, and psychoeducational functioning in neurofibromatosis type 1. *Am. J. Med. Genet. A* **188**, 2277–2292 (2022).
11. Yang, L. *et al.* IGF1R variants in patients with growth impairment: Four novel variants and genotype-phenotype correlations. *J. Clin. Endocrinol. Metab.* **103**, 3939–3944 (2018).
12. Epi4K Consortium. De Novo mutations in SLC1A2 and CACNA1A are important causes of epileptic encephalopathies. *Am. J. Hum. Genet.* **99**, 287–298 (2016).
13. Helbig, K. L. *et al.* De Novo pathogenic variants in CACNA1E cause developmental and epileptic encephalopathy with contractures, macrocephaly, and dyskinesias. *Am. J. Hum. Genet.* **104**, 562 (2019).
14. Lassuthova, P. *et al.* Mutations in ATP1A1 cause dominant Charcot-Marie-tooth type 2. *Am. J. Hum. Genet.* **102**, 505–514 (2018).
15. Paul, M. S. *et al.* A syndromic neurodevelopmental disorder caused by rare variants in PPFIA3. *Am. J. Hum. Genet.* **111**, 1239 (2024).
16. Butler, K. M. *et al.* De novo variants in GABRA2 and GABRA5 alter receptor function and contribute to early-onset epilepsy. *Brain* **141**, 2392–2405 (2018).
17. Du, W. *et al.* Calcium-sensitive potassium channelopathy in human epilepsy and paroxysmal movement disorder. *Nat. Genet.* **37**, 733–738 (2005).
18. Lise, S. *et al.* Recessive mutations in SPTBN2 implicate β-III spectrin in both cognitive and motor development. *PLoS Genet.* **8**, e1003074 (2012).
19. Tabarki, B., AlMajhad, N., AlHashem, A., Shaheen, R. & Alkuraya, F. S. Homozygous KCNMA1 mutation as a cause of cerebellar atrophy, developmental delay and seizures. *Hum. Genet.* **135**, 1295–1298 (2016).
20. Elsayed, S. M. *et al.* Autosomal dominant SCA5 and autosomal recessive infantile SCA are allelic conditions resulting from SPTBN2 mutations. *Eur. J. Hum. Genet.* **22**, 286–288 (2014).
21. Granadillo, J. L. *et al.* Pathogenic variants in TNRC6B cause a genetic disorder characterised by developmental delay/intellectual disability and a spectrum of neurobehavioural phenotypes including autism and ADHD. *J. Med. Genet.* **57**, 717–724 (2020).

22. Chen, G. *et al.* GIGYF1 disruption associates with autism and impaired IGF-1R signaling. *J. Clin. Invest.* **132**, (2022).
23. A major segment of the neurofibromatosis type 1 gene: cDNA sequence, genomic structure, and point mutations. *Cell* **62**, 609 (1990).
24. AlAbdi, L. *et al.* Loss-of-function variants in MYCBP2 cause neurobehavioural phenotypes and corpus callosum defects. *Brain* **146**, 1373–1387 (2023).
25. Hengel, H. *et al.* Bi-allelic loss-of-function variants in BCAS3 cause a syndromic neurodevelopmental disorder. *Am. J. Hum. Genet.* **108**, 1069–1082 (2021).
26. Martin, H. C. *et al.* Quantifying the contribution of recessive coding variation to developmental disorders. *Science* **362**, 1161–1164 (2018).
27. Fridman, H., Khazeeva, G., Levy-Lahad, E., Gilissen, C. & Brunner, H. Reproductive and cognitive effects in carriers of recessive pathogenic variants. *bioRxiv* 2024.09.30.615774 (2024) doi:10.1101/2024.09.30.615774.
28. Judd, J. *et al.* Allele frequencies at recessive disease genes are mainly determined by pleiotropic effects in heterozygotes. *bioRxivorg* 2025.12.05.692665 (2025) doi:10.64898/2025.12.05.692665.
29. Heyne, H. O. *et al.* Mono- and biallelic variant effects on disease at biobank scale. *Nature* **613**, 519–525 (2023).
30. Chen, C.-Y. *et al.* The impact of rare protein coding genetic variation on adult cognitive function. *Nat. Genet.* **55**, 927–938 (2023).
31. Furia, F. *et al.* The phenotypic and genotypic spectrum of individuals with mono- or biallelic ANK3 variants. *Clin. Genet.* **106**, 574–584 (2024).
32. Prasad, A. *et al.* A discovery resource of rare copy number variations in individuals with autism spectrum disorder. *G3 (Bethesda)* **2**, 1665–1685 (2012).
33. St Pourcain, B. *et al.* Common variation near ROBO2 is associated with expressive vocabulary in infancy. *Nat. Commun.* **5**, 4831 (2014).
34. Marchman, V. A. & Fernald, A. Speed of word recognition and vocabulary knowledge in infancy predict cognitive and language outcomes in later childhood. *Dev. Sci.* **11**, F9–16 (2008).
35. Reijnders, M. R. F. *et al.* De Novo and inherited loss-of-function variants in TLK2: Clinical and genotype-phenotype evaluation of a distinct neurodevelopmental disorder. *Am. J. Hum. Genet.* **102**, 1195–1203 (2018).
36. Villamor-Payà, M. *et al.* Identification of a de novo mutation in TLK1 associated with a neurodevelopmental disorder and immunodeficiency. *medRxiv* (2023) doi:10.1101/2023.08.22.23294267.
37. Vissers, L. E. L. M. *et al.* Mutations in a new member of the chromodomain gene family cause CHARGE syndrome. *Nat. Genet.* **36**, 955–957 (2004).
38. Sugathan, A. *et al.* CHD8 regulates neurodevelopmental pathways associated with autism spectrum disorder in neural progenitors. *Proc. Natl. Acad. Sci. U. S. A.* **111**, E4468–77 (2014).
39. Li, M. *et al.* Integrative functional genomic analysis of human brain development and neuropsychiatric risks. *Science* **362**, eaat7615 (2018).
40. Iossifov, I. *et al.* The contribution of de novo coding mutations to autism spectrum disorder. *Nature* **515**, 216–221 (2014).
41. Satterstrom, F. K. *et al.* Large-Scale Exome Sequencing Study Implicates Both Developmental and Functional Changes in the Neurobiology of Autism. *Cell* **180**, 568–584.e23 (2020).
42. Lim, E. T. *et al.* Rates, distribution and implications of postzygotic mosaic mutations in autism spectrum disorder. *Nat. Neurosci.* **20**, 1217–1224 (2017).
43. Degenhardt, F. *et al.* Duplications in RB1CC1 are associated with schizophrenia; identification in large European sample sets. *Transl. Psychiatry* **3**, e326 (2013).
44. Bochdanovits, Z. *et al.* Joint reanalysis of 29 correlated SNPs supports the role of PCLO/Piccolo as a causal risk factor for major depressive disorder. *Mol. Psychiatry* **14**, 650–652 (2009).

45. Nievergelt, C. M. *et al.* Discovery of 95 PTSD loci provides insight into genetic architecture and neurobiology of trauma and stress-related disorders. *medRxiv* (2023) doi:10.1101/2023.08.31.23294915.
46. International Mouse Phenotyping Consortium. Mouse gene Dpp8.
47. International Mouse Phenotyping Consortium. Mouse gene Ipo9.
48. Kaplanis, J. *et al.* Evidence for 28 genetic disorders discovered by combining healthcare and research data. *Nature* **586**, 757–762 (2020).
49. Samocha, K. E. *et al.* A framework for the interpretation of de novo mutation in human disease. *Nat. Genet.* **46**, 944–950 (2014).
50. Koko, M. *et al.* Exome sequencing of UK birth cohorts. *Wellcome Open Research* (2024).
51. Ji, Z., Li, Y., Liu, S. X. & Sharrocks, A. D. The forkhead transcription factor FOXK2 premarks lineage-specific genes in human embryonic stem cells for activation during differentiation. *Nucleic Acids Res.* **49**, 1345–1363 (2021).
52. van der Heide, L. P. *et al.* FoxK2 is required for cellular proliferation and survival: FoxK2 REGULATES PROLIFERATION AND CELLULAR SURVIVAL. *J. Cell. Physiol.* **230**, 1013–1023 (2015).
53. Polderman, T. J. C. *et al.* Meta-analysis of the heritability of human traits based on fifty years of twin studies. *Nat. Genet.* **47**, 702–709 (2015).
54. Tan, T. *et al.* Family-GWAS reveals effects of environment and mating on genetic associations. *Genetic and Genomic Medicine* (2024).
55. Lee, J. J. *et al.* Gene discovery and polygenic prediction from a 1.1-million-person GWAS of educational attainment. *Nat. Genet.* **50**, 1112 (2018).
56. Okbay, A. *et al.* Polygenic prediction of educational attainment within and between families from genome-wide association analyses in 3 million individuals. *Nat. Genet.* **54**, 437–449 (2022).
57. Karlsson Linnér, R. *et al.* Genome-wide association analyses of risk tolerance and risky behaviors in over 1 million individuals identify hundreds of loci and shared genetic influences. *Nat. Genet.* **51**, 245–257 (2019).
58. Baselmans, B. *et al.* The genetic and neural substrates of externalizing behavior. *Biol. Psychiatry Glob. Open Sci.* **2**, 389–399 (2022).
59. Open Targets Platform. https://platform.opentargets.org/credible-set/651a7e1654d0a7f9a9dde0f4850c0365.
60. Speidel, D. *et al.* A family of Ca2+-dependent activator proteins for secretion. *J. Biol. Chem.* **278**, 52802–52809 (2003).
61. Lee, J. J. *et al.* Gene discovery and polygenic prediction from a genome-wide association study of educational attainment in 1.1 million individuals. *Nat. Genet.* **50**, 1112–1121 (2018).
62. Sadakata, T. *et al.* Autistic-like phenotypes in Cadps2-knockout mice and aberrant CADPS2 splicing in autistic patients. *J. Clin. Invest.* **117**, 931–943 (2007).
63. Bulik-Sullivan, B. K. *et al.* LD Score regression distinguishes confounding from polygenicity in genome-wide association studies. *Nat. Genet.* **47**, 291–295 (2015).
64. Bulik-Sullivan, B. *et al.* An atlas of genetic correlations across human diseases and traits. *Nat. Genet.* **47**, 1236–1241 (2015).
65. Watanabe, K., Taskesen, E., van Bochoven, A. & Posthuma, D. Functional mapping and annotation of genetic associations with FUMA. *Nat. Commun.* **8**, 1826 (2017).
66. Young, A. I. *et al.* Mendelian imputation of parental genotypes improves estimates of direct genetic effects. *Nat. Genet.* **54**, 897–905 (2022).
67. Boyd, A. *et al. The 'Children of the 90s'; the Index Offspring of The Avon Longitudinal Study of Parents and Children*. (ALSPAC).
68. Explore data and samples. https://www.bristol.ac.uk/alspac/researchers/our-data/.
69. Moore, C. *et al.* The INTERVAL trial to determine whether intervals between blood donations can be safely and acceptably decreased to optimise blood supply: study protocol for a randomised controlled trial. *Trials* **15**, 363 (2014).
70. Manichaikul, A. *et al.* Robust relationship inference in genome-wide association studies.

Bioinformatics **26**, 2867–2873 (2010).
71. McCarthy, S. *et al.* A reference panel of 64,976 haplotypes for genotype imputation. *Nat. Genet.* **48**, 1279–1283 (2016).
72. Gardner, E. J. *et al.* Reduced reproductive success is associated with selective constraint on human genes. *Nature* **603**, 858–863 (2022).
73. Astle, W. J. *et al.* The Allelic landscape of human blood cell trait variation and links to common complex disease. *Cell* **167**, 1415–1429.e19 (2016).
74. Sun, B. B. *et al.* Genomic atlas of the human plasma proteome. *Nature* **558**, 73–79 (2018).
75. Murray, J. The ‘all of us’ research program. *N. Engl. J. Med.* **381**, 1884 (2019).
