# Supplementary material for: Imputation of fluid intelligence scores reduces ascertainment bias and increases power for analyses of common and rare variants
Source: Nat Genet. 2026 Oct 5;58(11):2784–93. doi: 10.1038/s41588-026-02787-5 (PMC13754570; doi:10.1038/s41588-026-02787-5)
Supplement: Supplementary file 2 [file 41588_2026_2787_MOESM2_ESM.pdf]

nature portfolio

Corresponding author(s): Hilary C. Martin, Abdel Abdellaoui, Matthew E. Hurles

Last updated by author(s): Jul 23, 2026

Reporting Summary

Nature Portfolio wishes to improve the reproducibility of the work that we publish. This form provides structure for consistency and transparency in reporting. For further information on Nature Portfolio policies, see our Editorial Policies and the Editorial Policy Checklist.

Statistics

For all statistical analyses, confirm that the following items are present in the figure legend, table legend, main text, or Methods section.

n/a	Confirmed	
☐	☒	The exact sample size (*n*) for each experimental group/condition, given as a discrete number and unit of measurement
☐	☒	A statement on whether measurements were taken from distinct samples or whether the same sample was measured repeatedly
☐	☒	The statistical test(s) used AND whether they are one- or two-sided<br>*Only common tests should be described solely by name; describe more complex techniques in the Methods section.*
☐	☒	A description of all covariates tested
☐	☒	A description of any assumptions or corrections, such as tests of normality and adjustment for multiple comparisons
☐	☒	A full description of the statistical parameters including central tendency (e.g. means) or other basic estimates (e.g. regression coefficient) AND variation (e.g. standard deviation) or associated estimates of uncertainty (e.g. confidence intervals)
☐	☒	For null hypothesis testing, the test statistic (e.g. *F*, *t*, *r*) with confidence intervals, effect sizes, degrees of freedom and *P* value noted<br>*Give P values as exact values whenever suitable.*
☒	☐	For Bayesian analysis, information on the choice of priors and Markov chain Monte Carlo settings
☒	☐	For hierarchical and complex designs, identification of the appropriate level for tests and full reporting of outcomes
☐	☒	Estimates of effect sizes (e.g. Cohen's *d*, Pearson's *r*), indicating how they were calculated

Our web collection on statistics for biologists contains articles on many of the points above.

Software and code

Policy information about availability of computer code

Data collection	No software was used in the data collection
Data analysis	The open source software used for this study was: softImpute v1.4-1, LDSC 1.0, GenomicSEM v0.05, METAL v2020-05-05, FUMA v1.6, SNIPAR v0.0.19, bigsnpr v1.12.18, Regenie v4.1, ENSEMBL Variant Effect Predictor v104. Our code is available through GitHub https://github.com/dmvandenberg/fluid-intelligence-imputation and Zenodo (10.5281/zenodo.21167984)

For manuscripts utilizing custom algorithms or software that are central to the research but not yet described in published literature, software must be made available to editors and reviewers. We strongly encourage code deposition in a community repository (e.g. GitHub). See the Nature Portfolio guidelines for submitting code & software for further information.

Data

Policy information about availability of data

All manuscripts must include a data availability statement. This statement should provide the following information, where applicable:

- Accession codes, unique identifiers, or web links for publicly available datasets
- A description of any restrictions on data availability
- For clinical datasets or third party data, please ensure that the statement adheres to our policy

UK Biobank data are available to researchers upon application (https://www.ukbiobank.ac.uk/enable-your-research/apply-for-access). Summary statistics from

population and within-family based GWAS of combined FIS, measured FIS, Imputed FIS, and the meta-analysis of combined FIS and COGENT can be found on Zenodo (10.5281/zenodo.21167984). Full results of rare variant analyses, including gene-level association statistics are available in the Supplements.

Research involving human participants, their data, or biological material

Policy information about studies with human participants or human data. See also policy information about sex, gender (identity/presentation), and sexual orientation and race, ethnicity and racism.

Reporting on sex and gender	This study included both female and male participants. Analyses did not investigate sex-specific efffects. In GWAS analyses, we included genetic sex (UKB variable 22001) as a covariate.
Reporting on race, ethnicity, or other socially relevant groupings	This study included only participants of genetically inferred European ancestry (see Methods) to minimize confounding due to population stratifications in genetic analyses.
Population characteristics	A total of 502,536 participants (273,402 females and 229,134 males) aged between 37 and 73 years old were recruited in the UK between 2006 and 2010. The participants were recruited across 22 assessment centers throughout Great Britain in order to cover a variety of different settings providing socioeconomic and ethnic heterogeneity and urban–rural mix. See for more details Bycroft, C. et al (2018). The UK Biobank resource with deep phenotyping and genomic data. Nature 562, 203.
Recruitment	The UK Biobank ascertainment strategy was designed to capture sufficient variation in socioeconomic, urban–rural, and ethnic background. The participation rate however was 5.45% and was biased towards older, more healthy, and female residents. The UK Biobank sample does reflect nationally representative data sources to a significant degree.
Ethics oversight	UK Biobank has received ethical approval from the National Health Service North West Centre for Research Ethics Committee (reference: 11/NW/0382) and gave approval for this work.

Note that full information on the approval of the study protocol must also be provided in the manuscript.

Field-specific reporting

Please select the one below that is the best fit for your research. If you are not sure, read the appropriate sections before making your selection.

☒ Life sciences ☐ Behavioural & social sciences ☐ Ecological, evolutionary & environmental sciences

For a reference copy of the document with all sections, see nature.com/documents/nr-reporting-summary-flat.pdf

Life sciences study design

All studies must disclose on these points even when the disclosure is negative.

Sample size	The sample size was determined by UK Biobank; see for more details Bycroft, C. et al (2018). The UK Biobank resource with deep phenotyping and genomic data. Nature 562, 203.
Data exclusions	In order to avoid bias due to populations stratification, we excluded participants with non-European ancestry as determined by PCA on genome-wide SNPs (details are described in the Methods).
Replication	Replication was done in three independent cohorts with different measures of intelligence; ALSPAC, MCS and INTERVAL. All replications were in line with our conclusions.
Randomization	Randomization was not relevant as this was not an experimental design. We included all participants of UK Biobank who genetically cluster with those of European ancestries
Blinding	Blinding was not relevant as this was not an experimental design.

Reporting for specific materials, systems and methods

We require information from authors about some types of materials, experimental systems and methods used in many studies. Here, indicate whether each material, system or method listed is relevant to your study. If you are not sure if a list item applies to your research, read the appropriate section before selecting a response.

Materials & experimental systems

n/a	Involved in the study
☒	☐ Antibodies
☒	☐ Eukaryotic cell lines
☒	☐ Palaeontology and archaeology
☒	☐ Animals and other organisms
☒	☐ Clinical data
☒	☐ Dual use research of concern
☒	☐ Plants

Methods

n/a	Involved in the study
☒	☐ ChIP-seq
☒	☐ Flow cytometry
☒	☐ MRI-based neuroimaging

Plants

Seed stocks	n/a
Novel plant genotypes	n/a
Authentication	n/a
