# Supplementary material for: Imputation of fluid intelligence scores reduces ascertainment bias and increases power for analyses of common and rare variants
Source: Nat Genet. 2026 Oct 5;58(11):2784–93. doi: 10.1038/s41588-026-02787-5 (PMC13754570; doi:10.1038/s41588-026-02787-5)
Supplement: Supplementary file 3 [file 41588_2026_2787_MOESM3_ESM.pdf]

nature portfolio

Peer Review File

Imputation of fluid intelligence scores reduces ascertainment bias and increases power for analyses of common and rare variants

Corresponding Author: Mr David van den Berg

Version 0:

Decision Letter:

20th August 2025

Dear Mr. van den Berg,

Your Article "Imputation of fluid intelligence scores reduces ascertainment bias and increases power for analyses of common and rare variants" has been seen by three referees. You will see from their comments below that, while they find your work of potential interest, they have raised substantial points that need to be addressed. In light of these comments, we cannot accept the manuscript for publication at this time, but we would be very interested in considering a suitably revised version that addresses the referees' concerns.

We hope you will find the referees' comments useful as you decide how to proceed. If you wish to submit a substantially revised manuscript, please bear in mind that we will be reluctant to approach the referees again in the absence of major revisions.

To guide the scope of the revisions, the editors discuss the referee reports in detail within the team, including with the chief editor, with a view to identifying key priorities that should be addressed in revision, and sometimes overruling referee requests that are deemed beyond the scope of the current study. In this case, we ask that you thoroughly address all technical points related to the imputation procedure and downstream association analyses, clarifying and extending the analyses as requested and revising the presentation and interpretations where needed. We hope you will find this prioritized set of referee points to be useful when revising your study. Please do not hesitate to get in touch if you would like to discuss these issues further.

If you choose to revise your manuscript taking into account all reviewer and editor comments, please highlight all changes in the manuscript text file. At this stage, we will need you to upload a copy of the manuscript in MS Word .docx or similar editable format.

We are committed to providing a fair and constructive peer-review process. Do not hesitate to contact us if there are specific requests from the reviewers that you believe are technically impossible or unlikely to yield a meaningful outcome.

If revising your manuscript:

*1) Include a “Response to referees” document detailing, point-by-point, how you addressed each referee comment. If no action was taken to address a point, you must provide a compelling argument. This response will be sent back to the referees along with the revised manuscript.

*2) If you have not done so already, please begin to revise your manuscript so that it conforms to our Article format instructions, available here.
Refer also to any guidelines provided in this letter.

*3) Include a revised version of any required Reporting Summary: https://www.nature.com/documents/nr-reporting-summary.pdf
It will be available to referees (and, potentially, statisticians) to aid in their evaluation if the manuscript goes back for peer review.
A revised checklist is essential for re-review of the paper.

Please be aware of our guidelines on digital image standards.

EXTENDED DATA FIGURES

When re-submitting your manuscript, please ensure that any supplementary figures and tables that are crucial to the manuscript's conclusions are converted into Extended Data figures and tables to increase visibility of these data. Extended Data figures and tables are online-only (present in the online PDF and full-text HTML versions of the paper), peer-reviewed display items that provide essential background to the article but are not included in the main article due to space constraints. A maximum of ten Extended Data display items (figures and tables) is permitted.

You may use the link below to submit your revised manuscript and related files:

Link Redacted

Note: This URL links to your confidential home page and associated information about manuscripts you may have submitted, or that you are reviewing for us. If you wish to forward this email to co-authors, please delete the link to your homepage.

If you wish to submit a suitably revised manuscript we would hope to receive it within 3-6 months. If you cannot send it within this time, please let us know. We will be happy to consider your revision so long as nothing similar has been accepted for publication at Nature Genetics or published elsewhere. Should your manuscript be substantially delayed without notifying us in advance and your article is eventually published, the received date would be that of the revised, not the original, version.

Please do not hesitate to contact me if you have any questions or would like to discuss the required revisions further.

Nature Genetics is committed to improving transparency in authorship. As part of our efforts in this direction, we are now requesting that all authors identified as 'corresponding author' on published papers create and link their Open Researcher and Contributor Identifier (ORCID) with their account on the Manuscript Tracking System (MTS), prior to acceptance. ORCID helps the scientific community achieve unambiguous attribution of all scholarly contributions. You can create and link your ORCID from the home page of the MTS by clicking on 'Modify my Springer Nature account'. For more information, please visit www.springernature.com/orcid.

Thank you for the opportunity to review your work.

Sincerely,
Kyle

Kyle Vogan, PhD
Senior Editor
Nature Genetics
https://orcid.org/0000-0001-9565-9665

Referee expertise:

Referee #1: Genetics, behavioral traits, cognition

Referee #2: Genetics, behavioral traits, cognition

Referee #3: Genetics, behavioral traits, cognition

Reviewers' Comments:

Reviewer #1 (Remarks to the Author):

At a high level, in this paper, the authors explore using an imputed fluid intelligence measure to augment their GWAS sample size and perform follow-up analyses that have greater statistical power. Their main analyses are conducted using data from the UK Biobank, which collected fluid intelligence data on just under 300k individuals (some of whom had data collected over multiple waves) but for which fluid intelligence data was missing on over 150k. The authors begin with an exploration of various ways to aggregate the fluid intelligence measures when multiple waves are available. They find that all three approaches (measurement in first wave, first measurement, and mean measurement) produce GWAS results that have similar properties (e.g., heritability and genetic correlation estimates). After a phenotype curation step using GWAS by subtraction, they impute fluid intelligence scores for those with missing data. The GWAS using their imputed fluid intelligence measure also has similar properties to those of a set of GWAS summary statistics from the COGENT consortium, though some differences relative to the unimputed UKB summary statistics remain.

Their approach of using imputed phenotypes appears to be very successful at increasing statistical power. They find many more lead SNPs in the GWAS, produce a more predictive polygenic score, improve the precision of tissue enrichment analyses and identify many more implicated genes in a rare-variant analysis.

Overall, I think this paper is useful and well-written. My one main concern is easily addressed. This concern and several small concerns are described below:

Major concern:

1) I find that I'm not convinced that the authors' imputation procedure adequately mitigates any sort of contamination of their phenotype such that their new hits actually represent fluid intelligence rather than a composite phenotype based on the features they use in their imputation. While it is comforting that the genetic correlation between their imputed phenotype and the non-cognitive component of educational attainment is similar to the genetic correlation based on the COGENT consortium's intelligence GWAS, it is concerning that the genetic correlation is quite different than what is estimated when using unimputed fluid intelligence. It also appears that the genetic correlation with raw educational attainment and Household Income is higher when using imputed fluid intelligence rather than measured fluid intelligence. This suggests to me that their imputed fluid intelligence score may be capturing heritable socio-economic pathways rather that just raw fluid intelligence.

I wonder if this problem is due to the feature selection approach the authors describe in Supplementary Note 2. It appears that the authors use a variable to impute fluid intelligence if the variable showed a stronger genetic correlation with intelligence (from Savage et al.) than with their estimated non-cognitive component of imputed fluid intelligence. Looking at Supplementary Figure 8, it appears that many included phenotypes only barely satisfy this criterion, suggesting that those phenotypes may still contribute a lot of non-fluid-intelligence signal.

All of this discussion is related to a recently published paper (Miao et al., 2024), which was not cited by the authors, that describes the difficulties of inference and the risk of false positives when using imputed phenotypes. They propose a method that allows you to use imputed phenotype data to boost power but no expected bias. If they authors implement the method from this paper, do they get very different results? Are their reasons that they prefer their approach to that of Miao et al.?

Minor concerns:

2) In the introduction, the authors characterize direct effects as estimates that "remov[e] the effects of genetic nurture mediated by relatives", but genetic nurture isn't the only confound of population GWAS that is removed by within-family GWAS. It also removes biases from population stratification and the effect of assortative mating.

3) The paper is very dense in acronyms that may not be obvious to the average Nature Genetic reader, and which made it difficult for me to follow at times (e.g., NDC, PTV, FIS, NTC, vA, vB, vC, etc.). I'd recommend reducing the number of acronyms if possible.

4) Typo in General Lay summary: "We caully considered the goal of this research"

References:

Miao, J., Wu, Y., Sun, Z., Miao, X., Lu, T., Zhao, J., & Lu, Q. (2024). Valid inference for machine learning-assisted genome-wide association studies. Nature Genetics, 56(11), 2361-2369.

Reviewer #2 (Remarks to the Author):

Only a subset of UK Biobank volunteers took the “fluid intelligence” (FI) test, a subset apparently subject to far more selection or ascertainment bias than is widely known. Since so many phenotypes have been measured in the UK Biobank, it is possible to impute the FI scores of those who did not take the test. The authors did this with the SoftImpute algorithm of Mazunder, Hastie, and Tibshirani, which minimizes an objective function consisting of two terms: (1) the distance between the observed and imputed data matrices according to the Frobenius norm and (2) the product of a tuning parameter lambda and the sum of the singular values of the imputed data matrix. Intuitively, this algorithm seeks to fill in the missing values in such a way as to minimize the dimensionality (more precisely, the rank) of the imputed data matrix. My perusal of the relevant documentation and literature did not turn up anything on the robustness of this algorithm under various conditions (“data missing at random” and so forth), but judging by the results, I venture to say that it worked pretty well in this application. The heritability of imputed FI is lower, as expected if the imputed values are noisier than the observed ones, but the constancy of LDSC genetic correlations with other variables suggests that the noise does not introduce bias. With a larger effective sample size, the authors were able to identify more genome-wide significant hits, more likely causal genes mediating the small effects of common SNPs, and more genes where rare variants have at least moderate effects. The accuracy of polygenic prediction increased somewhat in validation samples.

I regard the most intriguing empirical finding of this paper to be the decline in the magnitude of the correlation between so-called direct genetic effects (i.e., causal effects estimated within families) and non-transmitted coefficients (i.e., biases) upon

the use of imputation. This correlation went from substantially negative to near zero. This seems to provide strong support for the negative correlation between direct genetic effects and non-transmitted coefficients, in the case of intelligence, being the result of selection or ascertainment bias.

I am perhaps not competent to review this paper. I learned a lot from it about rare-variant analysis, and there is probably little authoritative or useful I could say to these authors about that aspect.

I hesitated to endorse the publication of this paper in Nature Genetics because I worry that it will encourage the practice of substituting prediction for measurement of psychological traits. McDonald (2011) defined measurement (as opposed to prediction) as an attempt to assess some behavioral trait with items or questions explicitly calling forth instances of the relevant behavior — an attempt that may be accompanied by an error of measurement. In the case of an intelligence test, the attempt amounts to asking the examinee a number of questions with objectively correct answers. Because of examinee-by-item interactions and genuine stochasticity, the (weighted) sum of correct answers may deviate from the examinee's "true score" or "factor score," and this deviation is the error of measurement in this case. Prediction was defined to be an attempt to assign a number to an individual coming as close to the true/factor score as possible, e.g. by using a multiple-regression equation. There are a number of differences between measurement and prediction. For starters, the variance of a measurement is greater than the variance of the actual attribute (because the variance of the former is the true variance plus the variance due to measurement error) while the variance of a prediction is smaller (because the coefficient of determination must be less than one). In the multivariate case, measurements and predictions need not have the same rank order. Without getting into all the details of paradoxes that might arise from relying on predictions rather than measurements, McDonald expressed a strong preference for the latter. He used the example of an unreliable English test that is correlated with a reliable math test. If we are using predictions, then most of our information about an examinee's English ability might come from the math rather than the English test. "I believe further discussion is needed. My personal view rests on the common-sense instantial argument that to measure an individual's ability at English we should use only English, and not increase the score on the basis of high performance in mathematics. More generally, to measure one attribute we should use just indicators—specific measures—of that attribute, without adjusting the score on the basis of other, conceptually distinct attributes" (McDonald, 2011, p. 534).

One consequence of a willingness to use predictions is "conceptual drift." Once we shrug at the use of variables other than questions with objectively correct answers as predictors of intelligence, merely because these other variables are correlated with existing IQ tests, some researchers might come to act as if they are indeed *measures* of intelligence. This does damage to the implicit definition of intelligence; "intelligence is whatever IQ tests measure" is not such a bad definition if you have the right tests, a disastrous definition if everything starts getting included. A colleague once argued to me that something like left-wing political views, which are positively correlated with IQ scores, might well be regarded as a legitimate *measure* of intelligence rather than an interesting correlate. Conceptual drift can also have untoward effects on the downstream results obtained by researchers who are less skillful or scrupulous than the authors of the present manuscript.

Nevertheless, because of the many interesting results (e.g., rare-variant associations), I come down on the side of endorsing this manuscript for publication. What I ask of the authors is a clear call for biobank curators to obtain high-quality *measurements* of phenotypes and to actively find ways to minimize selection/ascertainment bias. Prediction or imputation might work in a given situation, but it should be made clear that this is not a research strategy or an all-purpose backstop.

I have been asked by the journal to inspect the code posted by the authors on GitHub and confirm that it runs. After spending an entire working week on reviewing this paper, days past the deadline, I decline to take on this additional task, which the journal did not require in the past. Sometimes code is clearer than words, but the words need to be clear in any case. I already have struggled to get some of the software used by the authors running on my system. I have confidence in these authors and do not feel that anything useful can be gained by my inspection of their code.

ADDITIONAL MAJOR POINTS

p. 6: "Additionally, the point estimate for rdirect-NTC was closer to zero than was observed using measured FIS alone, and was not significantly different from zero (Figure 1F), suggesting that imputation further ameliorated the ascertainment bias observed when using only the measured FI values." Is it possible to obtain the significance of the difference between FIS1 and Combined FIS? It seems that the block jackknife should work. Eyeballing the confidence intervals suggests that the two correlations are indeed different. I am also wondering why the confidence intervals widen as we go from the top to the bottom of this figure. Naively it seems that they should decrease because of increasing sample size.

p. 7: "The same trend was observed for direct effect estimates obtained by controlling for parental PGIs in ALSPAC and MCS, suggesting the increased variance explained was at least partly due to improved within-family prediction." It must be mentioned that the children in ALSPAC and MSC are young enough that shared (common) environment accounts for a substantial share of the variance and genetic differences less (Tucker-Drob, Briley, & Harden, 2013). This is consistent with a high degree of passive gene-environment correlation in early life. With genetics, which contributes to within-family differences, mattering less, and the shared environment (including the genetics of the parents) mattering more, within-family segregation of the polygenic score will not predict the phenotype as well early in life. Some of the difference between population and direct in this comparison is likely explained by this developmental trend.

p. 31: "HapMap3+ SNPs" What is a HapMap3+ SNP? That is, what does the "+" mean? Also, might it be necessary to ensure that the imputation quality of all SNPs used in the polygenic score is very high, say, greater than 0.99? Tan et al. (2024) required a SNP to have an imputation accuracy of 99 percent in order to be included in its family-based GWAS. The

Supplementary Methods (SM, pp. 30-31) indicate that less stringent thresholds were applied. How the increased unreliability of differences can adversely affect the outcome is subtly different between family-based GWAS and polygenic prediction. In the former an unreliable difference in genotypes within families can lead to a bad beta; in the latter, even if we have little confounding in the training GWAS and a reasonable beta, an unreliable difference in genotypes within families in the validation sample can contribute noise to a difference in polygenic scores. Note that a polygenic score can be based on fewer than 500,000 SNPs and still tag all common SNPs.

Supplementary Material, pp. 10-11: Supplementary Note 2 is arguably crucial to the whole paper, but it is tough going. The last two paragraphs are particularly hard to understand and possibly contain errors. At the very least, the word “approaches” is accidentally duplicated. Please work to make this more comprehensible.

MINOR POINTS

p. 2: “direct genetic effects on measures of intelligence (i.e., the influence of an individual’s genotype on their own phenotype, removing the effects of genetic nurture mediated by relatives), and quantified the confounding due to residual population stratification and assortative mating.” -> “direct genetic effects on measures of intelligence within families, thereby removing biases from genetic nurture, residual population stratification, and assortative mating” Families are essential to mention in the introduction, since “direct genetic effects” is not a transparent term. The different sources of bias should be treated similarly here.

p. 4: “resemble those of the direct genetic effects after accounting for residual population stratification accounted for by LDSC regression” -> “resemble those of the direct genetic effects after accounting for residual population stratification with LDSC regression” Best to avoid repetition of “account for.”

Figure 1: The labels of panels E and F can be improved. “r direct – population” suggests that a subtraction is being performed, but this is not the case. I suggest “r(direct, population)” or perhaps “r(delta, beta)” as in Young et al. (2022), with explanations of the Greek letters in the caption. Similarly, the label of panel F should be “r(direct, NTC),” with perhaps a subscript “g” on the “r” to suggest that this is a genetic correlation estimated with LDSC.

Figure 2: “The direct effects were estimated in the two birth cohorts by additionally controlling for parental PGIs in the regressions.” Additionally? It is not necessary (and possibly detrimental) to control for anything else if you are already controlling for parental PGS. I can see controlling for sex and PCs if they did remove some variance and if it is thought that continuing to control for them makes for an apples-to-apples comparison.

Table 1: The caption should contain a reference to Supplementary Note 6, which describes the functions of some of the encoded proteins. It seems unbalanced to mention other phenotypes associated with these genes but not the known biological functions of their encoded proteins.

Table 1: “SKATO” -> “SKAT-O”

Table 1: Shouldn’t there be an explanation of what beta means? I’m guessing that it means the change in the phenotype per qualifying variant in the gene.

p. 14: “We were able to use GWAS-by-subtraction to refine the list of phenotypic variables used in the imputation from 154 to 82 variables” This is the first mention of GWAS-by-subtraction as a method used in this study. If the authors consider this important, perhaps the Discussion should not be the place to raise it for the first time.

p. 16: “Another limitation is that, due to the computational burden of applying multiple imputation, we analyzed only a single imputed dataset. This approach ignores imputation uncertainty, potentially biasing effect size estimates and standard errors.” If the GWAS standard errors are too small for this reason, will not correction with the LDSC intercept at least keep false positives among GWAS hits at the right level? Also, I would leave out “effect size estimates” and make the object of “biasing” just “standard errors.” Multiple imputation cannot reveal whether a given statistical estimate is biased in the strict sense.

p. 16: “However, the imputed phenotype correlated approximately 0.50 with the masked observed measure, below the test–retest reliability (~0.60), and preserved expected patterns of downstream genetic associations with other traits (e.g. Figure 1C), suggesting that the imputation introduced predominantly classical measurement error. Such measurement error likely biases effect size estimates toward zero” -> “The imputed phenotype correlated approximately 0.50 with the masked observed measure, below the test-retest reliability (~0.6), and preserved patterns of downstream genetic associations with other traits (e.g., Figure 1C), suggesting that the imputation introduced noise but not bias of the kind to disturb LDSC.” There are a number of problems with the original wording of the passage. The word “however” signals that what follows at least partially reverses what has come before. However … when the previous sentence and this one are revised in the manner that I suggest, there is no longer a close connection, even of opposition, between them. The first sentence concerns the possible underestimation of the GWAS standard errors as a result of creating only one imputed dataset, while the second concerns potential biases in the GWAS betas themselves regardless of whether their standard errors are correctly calibrated.

As I pointed out earlier, the procedure used by the authors would not be regarded by some psychometric purists as “classical measurement” or related to what is called “classical test theory,” making the term “classical measurement error” less than ideal. Hence my suggested revision removing the words “classical measurement.” The statement that “measurement error

likely biases effect size estimates toward zero" is a bit difficult to understand. If GWAS betas are biased toward zero, then why do the LDSC genetic correlations still seem to be accurate? Probably the reason for this is that if the imputed values are shrunk toward zero by a roughly uniform factor, then that factor cancels from the numerator and denominator in the LDSC estimate of the genetic correlation. But if this is not going to be explained to readers, the original wording will probably confuse at least some of them. Hence my suggested revision, which I think will avoid clashing intuitions.

p. 24: The description of SoftImpute omits the salient feature of the algorithm, its objective function.

p. 31: "We used LDpred2-auto98 to calculate PGIs in ALSPAC, MCS, and INTERVAL using our the" Omit either "our" or "the."

p. 32: "Enrichment of de novo variants in NDC probands" This is getting beyond my expertise, and I am happy to let the authors perform and present this analysis however they like. But they might consider trying to explain this more clearly to non-specialists. What is a "cumulative per-site mutation rate?" Is this a mutation rate for an entire protein-coding gene rather than a single site? Shouldn't the E have a "G" subscript or index as well as "i?" On a more substantive note, is a Poisson test assuming independence appropriate when mutations are known to be spatially autocorrelated? That is, if a proband has a mutation in one region, is not the observation of mutations nearby much more likely? Or is this taken care of by the "cumulative" nature of the mutation rate? And whatever the mutation rates mean or wherever they come from, is there not some statistical uncertainty associated with them that should be accounted for?

Supplementary Material, p. 4: "Frequently Asked Questions (FAQ)" I am opposed to FAQs or digressions in the Discussion section like this. The proper place for this often non-scientific piece of editorializing is an author's website or some other platform clearly distinct from the paper itself. This is not the worst one that I've read, but it still does not belong. To single out just one objection I have to this particular one: I regard embryo selection as a grave evil, but the section about this seems more like ass covering than serious engagement of the issue. It states that there are "ethical concerns" but does not really say what they are. There follow some "technical challenges" regarding the effectiveness of embryo selection that can be disputed. Finally, there is a legitimate ethical point at the end about unintended deleterious consequences of pleiotropy, but this seems to contradict the earlier suggestions about limited effectiveness. It is pretty weak to say that there are ethical problems and then avoid mentioning any of the problems that concern those most strongly opposed to embryo selection, including the incentivizing of the mass destruction of embryonic life. Other problems might take some space to explain, but that one is brief and uncomplicated. Are pro-life views verboten at Nature Genetics? (Do you see what I mean about sticking to science?)

Supplementary Material, pp. 12-13: Why does controlling for the *number* of singletons carried by an individual address fine-scale population structure? I can see why all those in the whole population carrying a *particular* variant observed as a singleton in a sample are probably related or come from the same locale. But why are those who carry similar totals of singletons more likely to come from the same fine-scale stratum? If different strata have expanded at different rates in the very recent past, then the total number of singletons might distinguish the strata. Is this the reason? Some readers might want to know more about this.

Supplementary Material, p. 13: "SKATO" -> "SKAT-O"

Supplementary Material, p. 13: "encoding an axon-binding receptor" -> "encoding a receptor expressed in the growth cones of extending axons"

Supplementary Material, p. 31: "the quality control of Whole exome sequencing data was described in the previous paper" Why is "Whole" capitalized? And what is the previous paper?

REFERENCES

McDonald, R. P. (2011). Measuring latent quantities. Psychometrika, 76, 511-536.

Tan, T. et al. (2024). Family-GWAS reveals effects of environment and mating on genetic associations. medRxiv.

Tucker-Drob, E.M., Briley, D.A., & Harden, K.P. (2013). Genetic and environmental influences on cognition across development and context. Current Directions in Psychological Science, 22, 349-355.

Young, A. (2022). Mendelian imputation of parental genotypes improves estimates of direct genetic effects. Nature Genetics, 54, 897-905.

Reviewer #3 (Remarks to the Author):

See attached.

Version 1:

Decision Letter:

27th March 2026

Dear David,

Your revised Article "Imputation of fluid intelligence scores reduces ascertainment bias and increases power for analyses of common and rare variants" has been seen by the original referees. You will see from their comments below and attached that, while they find the work substantially improved, they have raised a few ongoing concerns. We remain interested in the possibility of publishing your study in Nature Genetics, but we would like to consider your response to these points in the form of a further revision before we make a final decision on publication.

We therefore invite you to further revise your manuscript taking into account all of the reviewers' comments. Please highlight all changes in the manuscript text file. At this stage, we will need you to upload a copy of the manuscript in MS Word .docx or similar editable format.

We are committed to providing a fair and constructive peer-review process. Do not hesitate to contact us if there are specific requests from the reviewers that you believe are technically impossible or unlikely to yield a meaningful outcome.

When revising your manuscript:

*1) Include a “Response to referees” document detailing, point-by-point, how you addressed each referee comment. If no action was taken to address a point, you must provide a compelling argument. This response will be sent back to the referees along with the revised manuscript.

*2) If you have not done so already, please begin to revise your manuscript so that it conforms to our Article format instructions, available
here.
Refer also to any guidelines provided in this letter.

*3) Include a revised version of your Reporting Summary: https://www.nature.com/documents/nr-reporting-summary.pdf
It will be available to referees (and, potentially, statisticians) to aid in their evaluation if the manuscript goes back for peer review.
A revised checklist is essential for re-review of the paper.

Please be aware of our guidelines on digital image standards.

EXTENDED DATA FIGURES

When re-submitting your manuscript, please ensure that any supplementary figures and tables that are crucial to the manuscript’s conclusions are converted into Extended Data figures and tables to increase visibility of these data. Extended Data figures and tables are online-only (present in the online PDF and full-text HTML versions of the paper), peer-reviewed display items that provide essential background to the article but are not included in the main article due to space constraints. A maximum of ten Extended Data display items (figures and tables) is permitted.

Please use the link below to submit your revised manuscript and related files:

Link Redacted

Note: This URL links to your confidential home page and associated information about manuscripts you may have submitted, or that you are reviewing for us. If you wish to forward this email to co-authors, please delete the link to your homepage.

We hope to receive your revised manuscript within 4-8 weeks. If you cannot send it within this time, please let us know.

Please do not hesitate to contact me if you have any questions or would like to discuss these revisions further.

Nature Genetics is committed to improving transparency in authorship. As part of our efforts in this direction, we are now requesting that all authors identified as ‘corresponding author’ on published papers create and link their Open Researcher and Contributor Identifier (ORCID) with their account on the Manuscript Tracking System (MTS), prior to acceptance. ORCID helps the scientific community achieve unambiguous attribution of all scholarly contributions. You can create and link your ORCID from the home page of the MTS by clicking on ‘Modify my Springer Nature account’. For more information, please visit www.springernature.com/orcid.

We look forward to seeing the revised manuscript and thank you for the opportunity to review your work.

Sincerely,
Kyle

Kyle Vogan, PhD
Senior Editor
Nature Genetics
https://orcid.org/0000-0001-9565-9665

Referee expertise:

Referee #1: Genetics, behavioral traits, cognition

Referee #2: Genetics, behavioral traits, cognition

Referee #3: Genetics, behavioral traits, cognition

Reviewers' Comments:

Reviewer #1 (Remarks to the Author):

In this revision, the authors have responded thoroughly to the responses of the reviewers and made substantial changes, including writing a very careful new supplementary note describing the theory motivating their imputation procedure. In my previous review, I raised two major concerns. First, I worried that their imputed phenotype was contaminated by the phenotypes they used for imputation in a way that affected the interpretation of their results. Second, I asked why their approach would be preferable to an approach like POP-GWAS, which accounts for such potential contamination. I also raise a handful of small concerns. The authors have done an excellent job addressing my second major concern and all of my small concerns; however, I remain somewhat concerned about contamination.

To be clear, the revisions have substantially improved the manuscript's treatment of the imputed phenotype, and I appreciate the care the authors have taken to engage this issue. In particular, I found the new Discussion language and Supplementary Note 2 helpful. The supplement now provides a clear framework for why mega-analysis of measured and imputed phenotypes could, under explicit assumptions, reduce ascertainment-related bias relative to relying only on the subset with directly measured FIS. I also appreciate the additional empirical work the authors have done to reduce concern about construct drift, including refinement of the imputation variable set, discussion of why an earlier version appeared too "EA-like," and the use of external genetic-correlation patterns and related diagnostics to evaluate whether the final combined FIS remains aligned with a cognitive phenotype. Together, these additions make the paper considerably stronger and make me more comfortable that the imputation is not simply producing an arbitrary proxy phenotype.

That said, I continue to have an important residual concern about how this limitation is framed across the manuscript. My overall instinct is that the imputation is likely largely reasonable and that the combined FIS is probably useful for discovery. However, I remain worried by the apparently substantial difference in the genetic correlation with educational attainment between observed FIS and combined FIS. Even if the overall genetic-correlation profile is broadly similar, and even if some negative-control traits look reassuring, the EA result still raises the possibility that at least some of the newly discovered signals may reflect variants associated with educational attainment through pathways that are not purely cognitive.

My concern is not that the authors have ignored this issue entirely; on the contrary, I think they have done high-quality work to identify it, reduce it, and discuss it more candidly than in the original version. Rather, I think the remaining uncertainty is not yet woven consistently enough through the manuscript. In several places, the paper still reads as though combined FIS can be treated largely as though it were a directly measured intelligence phenotype. For example, the abstract headlines the higher number of independent SNP associations, improved prediction, and new genes found from a rare variant analysis, but has no discussion on the limitations of these results. Even in the main text where the genetic correlations between combined FIS and EA (non-cognitive) are presented, the authors don't point out this discrepancy, instead saying that "Genetic correlations with height and NonCogEA consistent with those found with the COGENT GWAS." Instead, an acknowledgement of the limitations is only found in the Discussion and Supplementary Note 2, which support a more qualified interpretation: combined FIS is a pragmatically useful proxy phenotype that may improve on the selected observed-FIS sample, but the magnitude of the remaining construct-drift problem has not been fully quantified. I think the paper would be improved if this limitation were integrated more explicitly throughout the rest of the paper, especially when presenting downstream discoveries and biological interpretation, rather than being concentrated mainly in the limitations section.

Alternatively, the authors should be more explicit in their manuscript why bias due to contamination is unlikely to materially affect interpretation of the new findings. For example, the new supplement suggests that if the imputation is uncontaminated by other phenotypes, then the patterns of rg should be the same. This is largely true with the exception of EA, EA (non-cognitive), and household income. Given that this is a violation of the assumptions in the Supplement, is it possible to infer how much this might affect downstream results and how that compares to the bias from ascertainment that the authors focus on? Even if this cannot be fully resolved empirically, I think the manuscript should make this tradeoff more explicit and sustain that framing throughout.

Reviewer #2 (Remarks to the Author):

The authors went to great effort, perhaps more than necessary, to address the questions and concerns of the reviewers. More authors have been added to the team, apparently for their expertise in matters raised by the reviewers, but I have also added authors to my papers in similar circumstances and see no problem with this. An extensive supplementary note has been added, demonstrating that if errors in prediction/imputation of the true score (as opposed to measurement) have mean zero conditional on the predictors and are uncorrelated with genotypes, then many desirable consequences follow. The premises here cannot be proved, but the consequences can be empirically checked, and they do more or less hold in this case.

This manuscript seems suitable for publication in close to its current form. My comments and suggestions at this point are all fairly minor.

Main p. 6, lines 155-157: “and resemble those of the direct genetic effects after accounting for residual population stratification with LDSC regression” This clause was in the original submission, but I now find that I do not understand it. Perhaps consider omitting.

Main p. 7, lines 208-209: This new sentence is missing a word.

Main p. 29, line 748: There should be a period after “GWAS,” before the next sentence beginning with “SNP.”

Main p. 35, line 950: This Tan et al. paper is a preprint on medRxiv, currently under review at Nature. Why is the journal given as Genetic and Genomic Medicine?

Reviewer #3 (Remarks to the Author):

See attached.

Version 2:

Decision Letter:

Our ref: NG-A69343R1

27th May 2026

Dear David,

Your revised manuscript "Imputation of fluid intelligence scores reduces ascertainment bias and increases power for analyses of common and rare variants" (NG-A69343R1) has been seen by two of the original referees. As you will see from their comments below, they find that the paper has improved in revision, and therefore we will be happy in principle to publish your study in Nature Genetics as an Article pending final revisions to satisfy their remaining points and to comply with our editorial and formatting guidelines.

We are now performing detailed checks on your paper, and we will send you a checklist detailing our editorial and formatting requirements soon. Please do not upload the final materials or make any revisions until you receive this additional information from us.

Thank you again for your interest in Nature Genetics. Please do not hesitate to contact me if you have any questions.

Sincerely,
Kyle

Kyle Vogan, PhD
Senior Editor
Nature Genetics
https://orcid.org/0000-0001-9565-9665

Reviewer #1 (Remarks to the Author):

Overall, I am happy with this revision. The revised manuscript now more clearly acknowledges that combined FIS has higher genetic correlations with EA, EA non-cognitive, and household income than observed FIS, and it appropriately notes in several places that some residual non-cognitive or SES-related signal may remain. I think these changes are sufficient.

I do want to note one remaining point about the response letter, though I do not think it requires further changes to the manuscript. It appears that combined FIS is more similar to COGENT than to observed FIS when looking at the broader pattern of genetic correlations. In the response letter, but not in the manuscript, the authors explain that NonCogEA is

constructed to be genetically uncorrelated with observed FIS, so the differences seen there may be due to artifacts from the way NonCogEA was constructed. They hypothesize that COGENT may remain correlated with NonCogEA because it was only a small component of the cognitive GWAS used in constructing NonCogEA. However, these arguments do not explain why combined FIS differs from observed FIS for household income, for example, which does not have the same issue. There are many possible explanations for this pattern, including that COGENT may also contain some non-cognitive signal, but as the authors explain, I do not think there is a way to fully resolve this empirically. More importantly, the revised manuscript itself now seems appropriately cautious on this issue. Additional revisions to discuss this point would likely make the manuscript more unwieldy with little benefit, so I am happy with the manuscript as it is written.

Reviewer #3 (Remarks to the Author):

The authors have again been highly responsive to my comments. I have only some minor comments/suggestions:

Main text:

1. Lines 74-75: The text claims that Supplementary Note 2 provides conditions for unbiased genetic effect estimates, but I believe Theorem 5 shows consistency, not unbiasedness.

2. Lines 178-179: It isn't clear what "latent trait" refers to.

3. Line 452: It isn't clear what kind of "technical artefact" you have in mind.

Supplementary Note 2:

4. Assumptions 3 and 5 use the terminology "the conditional first two moments" but formally only mention conditional expectations and conditional covariance, not the remaining second moments (conditional variances). I suggest changing the terminology to "the conditional expectations and conditional covariance."

5. Proof of Corollary 3: I did not follow "Substituting these expressions into Equation (9) gives Equation (11)." Could you spell out the steps in a bit more detail?

6. Remark 10, typo: "indepdent"

7. Sections 8 and 9 are missing statements of what assumptions are needed.

8. Section 8.1: What is Z? I don't think it has been defined previously.

9. Section 9: What is Gtagged? I don't think it has been defined previously. I also suggest reminding the reader what Y* is.

10. Section 10.1: Some of the notation is not defined.

11. Remark 12: I think you meant "specification check" rather than "specificity check."

12. Section 11: The terminology "complexity of measurement" and "complexity of prediction" strike me as odd and imprecise. "Complexity" has many meanings, and the term "prediction" is usually used when the object being predicted is the object of interest. Why not use the terminology "error of measurement" and "error of imputation"—or "measurement error" and "imputation error"—which I think would be more standard and more precise.

Signed,
Dan Benjamin

Open Access This Peer Review File is licensed under a Creative Commons Attribution 4.0 International License, which permits use, sharing, adaptation, distribution and reproduction in any medium or format, as long as you give appropriate credit to the original author(s) and the source, provide a link to the Creative Commons license, and indicate if changes were made.
In cases where reviewers are anonymous, credit should be given to 'Anonymous Referee' and the source.
The images or other third party material in this Peer Review File are included in the article's Creative Commons license, unless indicated otherwise in a credit line to the material. If material is not included in the article's Creative Commons license and your intended use is not permitted by statutory regulation or exceeds the permitted use, you will need to obtain permission directly from the copyright holder.
To view a copy of this license, visit https://creativecommons.org/licenses/by/4.0/

Editor

> To guide the scope of the revisions, the editors discuss the referee reports in detail within the team, including with the chief editor, with a view to identifying key priorities that should be addressed in revision, and sometimes overruling referee requests that are deemed beyond the scope of the current study. In this case, we ask that you thoroughly address all technical points related to the imputation procedure and downstream association analyses, clarifying and extending the analyses as requested and revising the presentation and interpretations where needed. We hope you will find this prioritized set of referee points to be useful when revising your study. Please do not hesitate to get in touch if you would like to discuss these issues further.

We thank the editors for the opportunity to revise our manuscript and appreciate the clear guidance on revision priorities. In response, we have strengthened and clarified the imputation workflow and downstream association analyses by adding a theoretical framework linking our imputation diagnostics to explicit assumptions (new Supplementary Note 2), expanding robustness analyses and statistical tests, and improving the presentation for clarity. Notably, in response to reviewer concerns about heteroskedasticity, we re-ran the common-variant GWAS analyses using Regenie with robust standard errors, and repeated all downstream analyses that rely on these summary statistics; as a result, some common-variant summary statistics changed slightly, but the main conclusions are unchanged.

We have also responded to all remaining reviewer comments point-by-point below. We believe the manuscript has been substantially strengthened as a result of the editors' guidance and the reviewers' careful and constructive input.

Reviewer #1

At a high level, in this paper, the authors explore using an imputed fluid intelligence measure to augment their GWAS sample size and perform follow-up analyses that have greater statistical power. Their main analyses are conducted using data from the UK Biobank, which collected fluid intelligence data on just under 300k individuals (some of whom had data collected over multiple waves) but for which fluid intelligence data was missing on over 150k. The authors begin with an exploration of various ways to aggregate the fluid intelligence measures when multiple waves are available. They find that all three approaches (measurement in first wave, first measurement, and mean measurement) produce GWAS results that have similar properties (e.g., heritability and genetic correlation estimates). After a phenotype curation step using GWAS by subtraction, they impute fluid intelligence scores for those with missing data. The GWAS using their imputed fluid intelligence measure also has similar properties to those of a set of GWAS summary statistics from the COGENT consortium, though some differences relative to the unimputed UKB summary statistics remain.

Their approach of using imputed phenotypes appears to be very successful at increasing statistical power. They find many more lead SNPs in the GWAS, produce a more predictive polygenic score, improve the precision of tissue enrichment analyses and identify many more implicated genes in a rare-variant analysis.

Overall, I think this paper is useful and well-written.

We thank the reviewer for the careful reading and the clear summary of our approach and main findings. We appreciate the positive evaluation of the manuscript, and we address the reviewer's comments point-by-point below.

My one main concern is easily addressed. This concern and several small concerns are described below:

Major concern:

1) I find that I'm not convinced that the authors' imputation procedure adequately mitigates any sort of contamination of their phenotype such that their new hits actually represent fluid intelligence rather than a composite phenotype based on the features they use in their imputation. While it is comforting that the genetic correlation between their imputed phenotype and the non-cognitive component of educational attainment is similar to the genetic correlation based on the COGENT consortium's intelligence GWAS, it is concerning that the genetic correlation is quite different than what is estimated when using unimputed fluid intelligence. It also appears that the genetic correlation with raw educational attainment and Household Income is higher when using imputed fluid intelligence rather than measured fluid intelligence. This suggests to me that their imputed fluid intelligence score may be capturing heritable socio-economic pathways rather than just raw fluid intelligence.

I wonder if this problem is due to the feature selection approach the authors describe in Supplementary Note 2. It appears that the authors use a variable to impute fluid intelligence if the variable showed a stronger genetic correlation with intelligence (from Savage et al.) than with their estimated non-cognitive component of imputed fluid intelligence. Looking at Supplementary Figure 8, it appears that many included phenotypes only barely satisfy this criterion, suggesting that those phenotypes may still contribute a lot of non-fluid-intelligence signal.

We agree with the reviewer that the imputed fluid intelligence score should not be interpreted as a perfectly "pure" measure of fluid intelligence. Rather, it represents a less-ascertained proxy for cognitive performance that probably retains some socio-environmental signal, just as measured fluid intelligence does, albeit in different proportions. Importantly, despite this, the imputed phenotype improves prediction of directly measured cognitive outcomes in independent cohorts and substantially increases power to detect rare variant associations that are clearly relevant to cognition, as evidenced by the strong enrichment for genes with established roles in neurodevelopmental disorders. We acknowledge this in the discussion (page 15) now as follows: "*Because the imputation necessarily leverages variables associated with both cognitive performance*

and socio-environmental factors, the resulting phenotype probably reflects a mixture of cognitive and socio-environmental influences, as with measured FI (and many other traits), albeit in different proportions."

POP-GWAS method from Miao et al
All of this discussion is related to a recently published paper (Miao et al., 2024), which was not cited by the authors, that describes the difficulties of inference and the risk of false positives when using imputed phenotypes. They propose a method that allows you to use imputed phenotype data to boost power but no expected bias. If the authors implement the method from this paper, do they get very different results? Are there reasons that they prefer their approach to that of Miao et al.?

References: Miao, J., Wu, Y., Sun, Z., Miao, X., Lu, T., Zhao, J., & Lu, Q. (2024). Valid inference for machine learning-assisted genome-wide association studies. Nature Genetics, 56(11), 2361-2369.

We thank the reviewer for suggesting the Miao et al approach. This method is useful in the instance where the phenotypic measurements are missing at random, or at least such that the effect size estimated for a given SNP in a GWAS among the measured samples is unbiased. However, in the case of fluid intelligence, we know that this is almost certainly not the case, since educational attainment correlates with completion of the FI questionnaire (Supp Figure 2) and there is a negative correlation between the direct and population effects (Figure 1), which strongly suggests ascertainment bias. Thus, we do not believe the method is appropriate to use in this situation. As we show in the new Supplementary Note 2, with sufficiently informative imputation, even in the case where the measured individuals are a biased subset, one can recover an unbiased estimate of the effect size of a given SNP by pooling measured and imputed individuals into a single regression via mega-analysis. This is the approach we take here.

Minor concerns:

2) In the introduction, the authors characterize direct effects as estimates that "remov[e] the effects of genetic nurture mediated by relatives", but genetic nurture isn't the only confound of population GWAS that is removed by within-family GWAS. It also removes biases from population stratification and the effect of assortative mating.

We thank the reviewer and agree that within-family GWAS estimates are robust to multiple sources of confounding beyond indirect genetic effects, including population stratification and assortative mating. To avoid ambiguity, we have simplified the definition of "direct genetic effects" in that sentence and now define them more generally as the influence of an individual's genotype on their own phenotype, while separately noting the robustness of within-family designs to confounding from population stratification and assortative mating as follows (page 2): "*A recent within-family GWAS meta-analysis conducted by Tan et al.*[17] *estimated direct genetic effects on measures of intelligence within families, thereby removing biases from genetic nurture, residual population stratification, and assortative mating.*"

3) The paper is very dense in acronyms that may not be obvious to the average Nature Genetic reader, and which made it difficult for me to follow at times (e.g., NDC, PTV, FIS, NTC, vA, vB, vC, etc.). I'd recommend reducing the number of acronyms if possible.

We agree that the manuscript contained a large number of acronyms, which could hinder readability. We have therefore reduced acronym use where possible. Specifically, we removed rarely used acronyms (e.g., vA, vB, vC, NDC) and replaced acronyms for imputed measures in the Supplementary Note with descriptive terms (e.g., "Average imputed FIS"), consistent with the naming of the observed measures.

4) Typo in General Lay summary: "We caully considered the goal of this research"

Thank you, this has been fixed.

Reviewer #2

Only a subset of UK Biobank volunteers took the "fluid intelligence" (FI) test, a subset apparently subject to far more selection or ascertainment bias than is widely known. Since so many phenotypes have been measured in the UK Biobank, it is possible to impute the FI scores of those who did not take the test. The authors did this with the SoftImpute algorithm of Mazunder, Hastie, and Tibshirani, which minimizes an objective function consisting of two terms: (1) the distance between the observed and imputed data matrices according to the Frobenius norm and (2) the product of a tuning parameter lambda and the sum of the singular values of the imputed data matrix. Intuitively, this algorithm seeks to fill in the missing values in such a way as to minimize the dimensionality (more precisely, the rank) of the imputed data matrix. My perusal of the relevant documentation and literature did not turn up anything on the robustness of this algorithm under various conditions ("data missing at random" and so forth), but judging by the results, I venture to say that it worked pretty well in this application. The heritability of imputed FI is lower, as expected if the imputed values are noisier than the observed ones, but the constancy of LDSC genetic correlations with other variables suggests that the noise does not introduce bias. With a larger effective sample size, the authors were able to identify more genome-wide significant hits, more likely causal genes mediating the small effects of common SNPs, and more genes where rare variants have at least moderate effects. The accuracy of polygenic prediction increased somewhat in validation samples.

I regard the most intriguing empirical finding of this paper to be the decline in the magnitude of the correlation between so-called direct genetic effects (i.e., causal effects estimated within families) and non-transmitted coefficients (i.e., biases) upon the use of imputation. This correlation went from substantially negative to near zero. This seems to provide strong support for the negative correlation between direct genetic effects and non-transmitted coefficients, in the case of intelligence, being the result of selection or ascertainment bias.

I am perhaps not competent to review this paper. I learned a lot from it about rare-variant analysis, and there is probably little authoritative or useful I could say to these authors about that aspect.

We thank the reviewer for their thoughtful and detailed assessment of our manuscript. We appreciate the clear summary of the study, including the discussion of the SoftImpute framework, and the emphasis on the decline in the direct effect–non-transmitted coefficient correlation as a key finding. We address the reviewer's specific comments and suggestions point-by-point below.

I hesitated to endorse the publication of this paper in Nature Genetics because I worry that it will encourage the practice of substituting prediction for measurement of psychological traits. McDonald (2011) defined measurement (as opposed to prediction) as an attempt to assess some behavioral trait with items or questions explicitly calling forth instances of the relevant behavior — an attempt that may be accompanied by an error of measurement. In the case of an intelligence test, the attempt amounts to asking the examinee a number of questions with objectively correct answers. Because of examinee-by-item interactions and genuine stochasticity, the (weighted) sum of correct answers may deviate from the examinee's "true score" or "factor score," and this deviation is the error of measurement in this case. Prediction was defined to be an attempt to assign a number to an individual coming as close to the true/factor score as possible, e.g. by using a multiple-regression equation. There are a number of differences between measurement and prediction. For starters, the variance of a measurement is greater than the variance of the actual attribute (because the variance of the former is the true variance plus the variance due to measurement error) while the variance of a prediction is smaller (because the coefficient of determination must be less than one). In the multivariate case, measurements and predictions need not have the same rank order. Without getting into all the details of paradoxes that might arise from relying on predictions rather than measurements, McDonald expressed a strong preference for the latter. He used the example of an unreliable English test that is correlated with a reliable math test. If we are using predictions, then most of our information about an examinee's English ability might come from the math rather than the English test. "I believe further discussion is needed. My personal view rests on the common-sense instantial argument that to measure an individual's ability at English we should use only English, and not increase the score on the basis of high performance in mathematics. More generally, to

measure one attribute we should use just indicators—specific measures—of that attribute, without adjusting the score on the basis of other, conceptually distinct attributes" (McDonald, 2011, p. 534).

One consequence of a willingness to use predictions is "conceptual drift." Once we shrug at the use of variables other than questions with objectively correct answers as predictors of intelligence, merely because these other variables are correlated with existing IQ tests, some researchers might come to act as if they are indeed *measures* of intelligence. This does damage to the implicit definition of intelligence; "intelligence is whatever IQ tests measure" is not such a bad definition if you have the right tests, a disastrous definition if everything starts getting included. A colleague once argued to me that something like left-wing political views, which are positively correlated with IQ scores, might well be regarded as a legitimate *measure* of intelligence rather than an interesting correlate. Conceptual drift can also have untoward effects on the downstream results obtained by researchers who are less skillful or scrupulous than the authors of the present manuscript.

Nevertheless, because of the many interesting results (e.g., rare-variant associations), I come down on the side of endorsing this manuscript for publication. What I ask of the authors is a clear call for biobank curators to obtain high-quality *measurements* of phenotypes and to actively find ways to minimize selection/ascertainment bias. Prediction or imputation might work in a given situation, but it should be made clear that this is not a research strategy or an all-purpose backstop.

We agree that prediction or imputation should not be conflated with measurement, particularly for psychological traits, and that uncritical use of predicted phenotypes risks conceptual drift.

In response, we have revised the Introduction and Discussion to explicitly distinguish measurement from prediction, to emphasize that imputation is a pragmatic, second-best response to limitations of existing biobank data, and to clarify that imputed phenotypes are not intended as substitutes for high-quality cognitive measurement. Specifically, in the Introduction (page 3) we now state:

"*We predicted this would ameliorate ascertainment bias and boost power for downstream genetic analyses, although we stress that imputation is not a substitute for high-quality cognitive measurement, which is costly and logistically challenging to collect at biobank scale.*"

In the Discussion (page 15-16), we further clarify the conceptual scope and limitations of imputation:

"*Finally, and more broadly, these results reflect a pragmatic response to limitations of existing biobank data rather than an endorsement of prediction/imputation as a substitute for measurement. While statistical imputation can improve power and reduce selection bias, it necessarily blurs the boundary between the construct of interest and its correlates, and imputed phenotypes should therefore not be treated as interchangeable with direct cognitive measurements.*"

We explicitly emphasize the importance of improved phenotyping and study design for future work as a conclusion to the limitations section (page 16):

"*Ultimately, the most robust path forward for genetic studies of cognition is improved phenotyping: the routine collection of high-quality, theory-driven cognitive measures alongside strategies to minimize selection and ascertainment bias at the design stage, rather than relying on post hoc statistical correction.*"

Finally, we want to note that our confidence is supported by external validation in cohorts with directly measured cognitive outcomes, which anchors interpretation to measurement-based constructs and helps mitigate concerns about conceptual drift. We agree that improved phenotyping remains the ideal, but recognize that high-quality cognitive measurement at biobank scale is costly and logistically challenging, motivating cautious use of imputation as a pragmatic complement rather than a substitute.

I have been asked by the journal to inspect the code posted by the authors on GitHub and confirm that it runs. After spending an entire working week on reviewing this paper, days past the deadline, I decline to take on this additional task, which the journal did not require in the past. Sometimes code is clearer than words, but the words need to be clear in any case. I already have struggled to get some of the software used by the authors running on my system. I have confidence in these authors and do not feel that anything useful can be gained by my inspection of their code.

We thank the reviewer for this vote of confidence and for the major effort and time put into providing useful feedback on our paper.

p. 6: "Additionally, the point estimate for $r_{direct\text{-}NTC}$ was closer to zero than was observed using measured FIS alone, and was not significantly different from zero (Figure 1F), suggesting that imputation further ameliorated the ascertainment bias observed when using only the measured FI values." Is it possible to obtain the significance of the difference between FIS1 and Combined FIS? It seems that the block jackknife should work. Eyeballing the confidence intervals suggests that the two correlations are indeed different.

We agree with the reviewer that formally testing the difference is appropriate. As they suggested, we have now tested the significance of differences between the various correlations shown using a block jackknife. (i.e. FIS1 versus Average FIS, FIS1 versus Combined FIS, and Combined FIS versus Average FIS). For each comparison, we aligned SNPs, ordered them by chromosome and created 200 blocks of about 5087 SNPs. Then we computed the leave-one-block out r(direct,NTC) for each of those blocks. Using the proposed jackknife significance test, we then computed the p-value of the differences. As the confidence intervals suggested, the difference between FIS1 and Combined FIS was significant ($p = 2.6 \times 10^{-4}$). The differences between FIS1 and Average FIS ($p = 0.011$), and Average FIS and Combined FIS ($p = 0.046$) were both marginally significant. We have added annotations indicating these significance tests to Figure 1F and Supplementary Figure 4G.

I am also wondering why the confidence intervals widen as we go from the top to the bottom of this figure. Naively it seems that they should decrease because of increasing sample size.

We agree that this pattern may appear counterintuitive. The precision of the r(direct, NTC) estimates is not determined solely by the number of trios, but by the joint precision of both the direct effect and nontransmitted coefficient estimates. Estimating a precise correlation between these components requires both to be well estimated. We quantified the genome-wide signal in the non-transmitted coefficient component analogously to the direct effects by calculating SNP h^2 using LDSC. For the NTC GWAS used in Figure 1F, SNP-heritability estimates were generally near zero and not statistically significant, with the exception of the Combined FIS phenotype (see Table), indicating limited polygenic signal in the nontransmitted component.

**NTC GWAS**	**h^2**	**SE**
Average observed FIS	-0.0205	0.0307
Earliest observed FIS	-0.0068	0.0329
observed FIS1	0.0077	0.0675
MegaFIS-average	0.0502	0.0215

When one side of the correlation is weakly estimated, the resulting correlation estimate has larger jackknife uncertainty, even if the number of trios increases. Although the NTC signal becomes detectable with increased sample size, it shows little overlap with the direct-effect signal, leading to a near-zero correlation estimate with wider confidence intervals.

> p. 7: “The same trend was observed for direct effect estimates obtained by controlling for parental PGIs in ALSPAC and MCS, suggesting the increased variance explained was at least partly due to improved within-family prediction.” It must be mentioned that the children in ALSPAC and MSC are young enough that shared (common) environment accounts for a substantial share of the variance and genetic differences less (Tucker-Drob, Briley, & Harden, 2013). This is consistent with a high degree of passive gene-environment correlation in early life. With genetics, which contributes to within-family differences, mattering less, and the shared environment (including the genetics of the parents) mattering more, within-family segregation of the polygenic score will not predict the phenotype as well early in life. Some of the difference between population and direct in this comparison is likely explained by this developmental trend.

We thank the reviewer for this important point regarding developmental differences in the relative contribution of shared environmental and genetic influences on cognitive ability. We agree that within-family genetic prediction is expected to be attenuated in early childhood, when shared environmental influences and passive gene–environment correlation play a larger role. Our intention in this paragraph was not to suggest that within-family prediction is large in absolute terms in ALSPAC or MCS, but rather that improvements in the discovery GWAS translate into improved prediction of the direct genetic component that is present. To avoid ambiguity, we have revised the wording to refer explicitly to improved prediction of direct genetic effects.

The quoted sentence in the Results (page 9) now reads as follows: “*PGI prediction in three independent cohorts showed that both the variance explained by the PGIs (Figure 2C, Supplementary Table 9) and direct effect estimates obtained by controlling for parental PGIs increased monotonically with sample size, validating our integration and imputation approaches (Supplementary Note 6)*.”

> p. 31: “HapMap3+ SNPs” What is a HapMap3+ SNP? That is, what does the “+” mean? Also, might it be necessary to ensure that the imputation quality of all SNPs used in the polygenic score is very high, say, greater than 0.99? Tan et al. (2024) required a SNP to have an imputation accuracy of 99 percent in order to be included in its family-based GWAS. The Supplementary Methods (SM, pp. 30-31) indicate that less stringent thresholds were applied. How the increased unreliability of differences can adversely affect the outcome is subtly different between family-based GWAS and polygenic prediction. In the former an unreliable difference in genotypes within families can lead to a bad beta; in the latter, even if we have little confounding in the training GWAS and a reasonable beta, an unreliable difference in genotypes within families in the validation sample can contribute noise to a difference in polygenic scores. Note that a polygenic score can be based on fewer than 500,000 SNPs and still tag all common SNPs.

Apologies for not being clear in the Methods. HapMap3+ is an extended set of HapMap3 variants (~1.1M to ~1.4M) designed to maximize genome-wide tagging coverage, improving performance across diverse ancestries compared to the original set. We have now clarified this in the Methods (p. 31): “We used unrelated individuals with European ancestries (ascertained as described in the Supplementary Methods) and generated LD reference panels restricting to HapMap3+ SNPs (Privé et al. 2023), which are designed to maximize genome-wide tagging coverage, improving performance across diverse ancestries compared to the original set.”

The reviewer raises a good point about the imputation quality threshold for family-based GWAS and polygenic prediction. We have now repeated the within-family GWAS using ~1.1M SNPs with INFO score > 0.98 (vs. 900k SNPs at INFO>.99), and have also repeated the PGI analyses in external cohorts with the new summary statistics generated with Regenie. We have set an INFO score threshold of >0.98 to ensure the SNPs were well imputed while retaining a sufficient number of variants in all cohorts to conduct the PGI analyses. None of the results qualitatively changed.

Supplementary Material, pp. 10-11: Supplementary Note 2 is arguably crucial to the whole paper, but it is tough going. The last two paragraphs are particularly hard to understand and possibly contain errors. At the very least, the word "approaches" is accidentally duplicated. Please work to make this more comprehensible.

We thank the reviewer for pointing this out. We have restructured Supplementary Note 3 (previously Supplementary Note 2) and added subheadings for each iteration of imputation. We also fixed some errors in nomenclature and reduced the number of acronyms used to make it more comprehensible. Additionally we added some text regarding Supplementary Note 3 to the main text (p. 6-7), in response to a comment from Reviewer 3: "*To arrive at a robust imputation strategy, we went through several iterations. We started with refining our set of imputation variables, beginning with 154 variables phenotypically correlated with intelligence which were then subsetted to 82 variables whose genetic correlation profile was more aligned with intelligence than with the residual non-cognitive signal observed in the first imputation iteration (see Methods). Then, we evaluated three approaches to imputing FIS (Supplementary Figure 5), namely through imputing FIS using the earliest FIS measure for observed individuals, the average measure for observed individuals, or all available FIS measures. These approaches were evaluated using imputation accuracy, SNP heritability (population-based and within-family), genetic correlations with external traits, and the correlation between direct effects and non-transmitted coefficients. In Supplementary Note 2, we outline the theoretical framework formalising what our phenotype imputation is trying to achieve, and show how these evaluation metrics relate to this framework*"

MINOR POINTS

p. 2: "direct genetic effects on measures of intelligence (i.e., the influence of an individual's genotype on their own phenotype, removing the effects of genetic nurture mediated by relatives), and quantified the confounding due to residual population stratification and assortative mating." -> "direct genetic effects on measures of intelligence within families, thereby removing biases from genetic nurture, residual population stratification, and assortative mating" Families are essential to mention in the introduction, since "direct genetic effects" is not a transparent term. The different sources of bias should be treated similarly here.

We agree and have revised this sentence to make the within-family context explicit, as suggested, and to describe the different sources of bias in a more consistent way. The revised sentence on page 2 now reads: "*A recent within-family GWAS meta-analysis conducted by Tan et al.*[17] *estimated direct genetic effects on measures of intelligence within families, thereby removing biases from genetic nurture, residual population stratification, and assortative mating.*"

p. 4: "resemble those of the direct genetic effects after accounting for residual population stratification accounted for by LDSC regression" -> "resemble those of the direct genetic effects after accounting for residual population stratification with LDSC regression" Best to avoid repetition of "account for."

Thank you, this has been changed as suggested.

Figure 1: The labels of panels E and F can be improved. "r direct – population" suggests that a subtraction is being performed, but this is not the case. I suggest "r(direct, population)" or perhaps "r(delta, beta)" as in Young et al. (2022), with explanations of the Greek letters in the caption. Similarly, the label of panel F should be "r(direct, NTC)," with perhaps a subscript "g" on the "r" to suggest that this is a genetic correlation estimated with LDSC.

Thank you, we changed the titles of figure 1E and 1F to r_g(direct, population) and r(direct, NTC) repectively.

Figure 2: “The direct effects were estimated in the two birth cohorts by additionally controlling for parental PGIs in the regressions.” Additionally? It is not necessary (and possibly detrimental) to control for anything else if you are already controlling for parental PGS. I can see controlling for sex and PCs if they did remove some variance and if it is thought that continuing to control for them makes for an apples-to-apples comparison.

We agree that, if one is interested in estimating PGI direct effects, it is arguably not necessary to control for PCs. However, as the Reviewer notes, we want to control for PCs in the population effect regressions and allow for an apples-to-apples comparison within and across cohorts. Importantly, the PGI analysis is primarily used to show the relative improvement in variance explained as opposed to the absolute variance explained by the PGIs.

Table 1: The caption should contain a reference to Supplementary Note 6, which describes the functions of some of the encoded proteins. It seems unbalanced to mention other phenotypes associated with these genes but not the known biological functions of their encoded proteins.

We have updated the Table caption to reference Supplementary Note 8 (previously 6).

Table 1: “SKATO” -> “SKAT-O”

We have updated the spelling in Table 1.

Table 1: Shouldn’t there be an explanation of what beta means? I’m guessing that it means the change in the phenotype per qualifying variant in the gene.

We thank the reviewer for pointing this out. We have updated the caption to specify that beta indicates the associated change in standard deviations of the phenotype given one qualifying variant in the gene.

p. 14: “We were able to use GWAS-by-subtraction to refine the list of phenotypic variables used in the imputation from 154 to 82 variables” This is the first mention of GWAS-by-subtraction as a method used in this study. If the authors consider this important, perhaps the Discussion should not be the place to raise it for the first time.

We have revised the Discussion to avoid introducing “GWAS-by-subtraction” at this point in the manuscript. Instead, we now describe this step more generally as a genetically informed refinement of the predictor set, while keeping methodological details in Supplementary Note 3. The revised text on page 14 now reads:

“*Our initial imputation used a broad set of correlated variables and achieved good accuracy (r=0.52 with measured FIS, comparable to the FIS test–retest correlation of r≈0.6), but showed inflated genetic correlations with traits such as NonCogEA and height (Supplementary Note 3; Supplementary Fig. 4). We therefore refined the predictor set (154 to 82 variables; Supplementary Note 3, Supplementary Figures 7 and 8) by retaining variables whose genetic correlation profile was more aligned with intelligence than with the residual non-cognitive signal observed in the first imputation. This attenuated the inflation in these correlations, with only a modest loss of accuracy (r=0.46).*”

p. 16: “Another limitation is that, due to the computational burden of applying multiple imputation, we analyzed only a single imputed dataset. This approach ignores imputation uncertainty, potentially biasing effect size estimates and standard errors.” If the GWAS standard errors are too small for this reason, will not correction with the LDSC intercept at least keep false positives among GWAS hits at the right level? Also, I would leave out “effect size estimates” and make the object of “biasing” just “standard errors.” Multiple imputation cannot reveal whether a given statistical estimate is biased in the strict sense.

We thank the reviewer for this very helpful comment. In line with this suggestion, we have made two sets of changes: (i) we have clarified the theoretical role of imputation uncertainty and its impact on estimates versus standard errors in the new Supplementary Note 2, and (ii) we have re-run all GWAS using heteroskedasticity-robust standard errors, so that our inference does not assume homoskedasticity, for the reasons explained below.

First, we now agree that our original wording was too strong. In the revised manuscript we no longer state that using a single imputed dataset “biases effect size estimates.” In the new Supplementary Note 2 we show that, under our core assumptions (imputation targets the same latent trait and the imputation error is uncorrelated with genotypes), the single-imputation GWAS slopes still converge to the same population estimands as in the fully observed case. In that sense, effect size estimates are not biased in the strict sense; the primary impact of ignoring imputation uncertainty is on the precision of those estimates (i.e., the standard errors and hence power). We have therefore rephrased the limitation to focus on potential underestimation of standard errors, not on bias of the point estimates. The revised segment (p. 14) now reads: “*This accuracy of 0.46 was below the test-retest reliability (~0.6), although cross-trait genetic correlations estimated from the combined FIS GWAS closely matched those from independent intelligence GWAS (for example, Figure 1C). These observations suggest that the imputation introduced additional noise but not systematic bias of the kind that would distort LDSC estimates, consistent with the theory we develop in Supplementary Note 2. The modestly lower LDSC-SNP h2 observed for combined FIS than observed FIS (0.16 versus 0.20; Figure 1) is likely due to this additional noise. Additionally, we note that the heteroskedasticity introduced during imputation can bias standard errors downward, so we used a heteroskedasticity-robust standard error estimator.*”

Second, to address directly the concern that a single imputed dataset and heterogeneous error variance could lead to downward-biased standard errors, we now explicitly fit the GWASs with heteroskedasticity-robust “sandwich” standard errors (HC3). This specification allows the residual variance to differ between measured and imputed individuals (and between measured individuals with different numbers of FI tests) and therefore does not rely on the homoskedasticity assumption that is most likely to be violated by our imputation scheme. Re-running the analyses with robust standard errors left the pattern of association signals essentially unchanged, with effect size estimates being virtually identical, and genetic correlations with our prior GWASs being >0.99 (SE = .001). We now state in the Methods that all reported GWAS results are based on these robust standard errors.

Third, regarding the LDSC intercept: we agree that, when there is a genome-wide inflation of test statistics, using the LDSC intercept can help keep the average false positive rate closer to nominal levels by rescaling test statistics. However, LDSC is designed to diagnose and correct broad-scale confounding (e.g., population stratification and cryptic relatedness) and polygenicity; it does not directly model SNP-specific heteroskedasticity arising from the mixture of measured and imputed phenotypes, nor does it fully account for the additional variability introduced by single imputation. In particular, LDSC intercept adjustment operates at the level of the mean inflation of chi-square statistics, whereas imputation-related uncertainty affects the variance of the regression residuals conditional on SNP and phenotype source. For this reason, we do not rely on LDSC intercept correction alone to guarantee valid standard errors. Instead, we use robust standard errors in the original GWAS fits to address heteroskedasticity and single-imputation uncertainty directly.

p. 16: “However, the imputed phenotype correlated approximately 0.50 with the masked observed measure, below the test–retest reliability (~0.60), and preserved expected patterns of downstream genetic associations with other traits (e.g. Figure 1C), suggesting that the imputation introduced predominantly classical measurement error. Such measurement error likely biases effect size estimates toward zero” -> “The imputed phenotype correlated approximately 0.50 with the masked observed measure, below the test-retest reliability (~0.6), and preserved patterns of downstream genetic associations with other traits (e.g., Figure 1C), suggesting that the imputation introduced noise but not bias of the kind to disturb LDSC.” There are a number of problems with the original wording of the passage. The word “however” signals that what follows at least partially reverses what has come before. However … when the previous sentence and this one are revised in the manner that I suggest, there is no longer a close connection, even of opposition, between them. The first sentence concerns the possible underestimation of the GWAS standard errors as a result of creating only one imputed dataset, while the second concerns potential biases in the GWAS betas themselves regardless of whether their standard errors are correctly calibrated.

As I pointed out earlier, the procedure used by the authors would not be regarded by some psychometric purists as “classical measurement” or related to what is called “classical test theory,” making the term “classical measurement error” less than ideal. Hence my suggested revision removing the words “classical measurement.” The statement that “measurement error likely biases effect size estimates toward zero” is a bit difficult to understand. If GWAS betas are biased toward zero, then why do the LDSC genetic correlations still seem to be accurate? Probably the reason for this is that if the imputed values are shrunk toward zero by a roughly uniform factor, then that factor cancels from the numerator and denominator in the LDSC estimate of the genetic correlation. But if this is not going to be explained to readers, the original wording will probably confuse at least some of them. Hence my suggested revision, which I think will avoid clashing intuitions.

We are grateful to the reviewer for these detailed comments and for the suggested rewording. In the revised manuscript we have substantially rewritten the relevant part of the Discussion and clarified the underlying reasoning in the new Supplementary Note.

First, we agree that our original paragraph conflated two distinct issues, namely the underestimation of standard errors due to using a single imputed dataset, and the impact of imputation noise on the GWAS betas themselves. We now separate these points clearly. The sentence about using a single imputed dataset appears in the context of standard errors and power only, and no longer refers to bias in effect sizes. The following sentence, which discusses the correlation between the imputed and masked observed scores and the downstream genetic associations, has been rephrased along the lines suggested by the reviewer: “*This accuracy of 0.46 was below the test-retest reliability (~0.6), although downstream analyses of the combined FIS GWAS preserved patterns of downstream genetic associations with other traits (for example, Figure 1C). These observations suggest that the imputation introduced additional noise but not systematic bias of the kind that would distort LDSC estimates, consistent with the theory we develop in Supplementary Note 2.*” We have removed the word “however,” since the two sentences now describe different phenomena rather than opposing points.

In making this change, we have dropped the phrase “classical measurement error” from the main text. We agree that, from a psychometric point of view, our setting would not be regarded as classical test theory in a strict sense, and using that term was unnecessarily strong. Now, in the second paragraph of the Discussion (third last paragraph) we instead refer more neutrally to the imputation as introducing “additional noise,” and we direct readers who want the formal conditions to the Supplementary Note, where we spell out when this noise can be treated as orthogonal to genotypes and not a source of new gene-environment correlation.

This new sentence is also intended to clarify the relationship between attenuation of effect sizes and the stability of LDSC genetic correlations, which the original phrasing (“measurement error likely biases effect size estimates toward zero”) could indeed make it seem contradictory. In the Supplementary Note we now explain that when imputation adds noise that is uncorrelated with genotypes, it increases the error variance of the phenotype while leaving the genetic covariance with SNPs unchanged. This shrinks the marginal GWAS betas and observed-scale SNP heritability toward zero by a roughly uniform factor. However, LDSC estimates of genetic correlation between our phenotype and an external trait depend on the ratio of the

genetic covariance to the square root of the product of heritabilities, so the same multiplicative factor appears in both numerator and denominator and cancels out. In the main text we describe this verbally rather than in equations and emphasize that, under our assumptions, additional imputation noise attenuates betas and heritability but does not generate the type of bias that would be expected to distort LDSC genetic correlations.

We believe these changes make the Discussion both more accurate and less confusing, and we are very grateful to the reviewer for highlighting the issues with our original wording and for the concrete suggestions that guided the revision.

p. 24: The description of SoftImpute omits the salient feature of the algorithm, its objective function.

We thank the reviewer for this comment and agree some wording is necessary to address this key feature of SoftImpute compared to multiple imputation algorithms. On page 26 we added the text *"It does so by minimizing an objective function consisting of two terms: (1) the distance between the observed entries in the observed and imputed data matrices according to the Frobenius norm, and (2) the product of a tuning parameter lambda and the sum of the singular values of the imputed data matrix. Intuitively, this means the algorithm fills in missing values in a way that minimizes the rank of the imputed data matrix, favouring low-dimensional structure over complexity."* largely as suggested by the reviewer.

p. 31: "We used LDpred2-auto98 to calculate PGIs in ALSPAC, MCS, and INTERVAL using our the" Omit either "our" or "the."

Thank you for catching this typo. We have removed "our" from the sentence.

p. 32: "Enrichment of de novo variants in NDC probands" This is getting beyond my expertise, and I am happy to let the authors perform and present this analysis however they like. But they might consider trying to explain this more clearly to non-specialists. What is a "cumulative per-site mutation rate?" Is this a mutation rate for an entire protein-coding gene rather than a single site? Shouldn't the E have a "G" subscript or index as well as "i?" On a more substantive note, is a Poisson test assuming independence appropriate when mutations are known to be spatially autocorrelated? That is, if a proband has a mutation in one region, is not the observation of mutations nearby much more likely? Or is this taken care of by the "cumulative" nature of the mutation rate? And whatever the mutation rates mean or wherever they come from, is there not some statistical uncertainty associated with them that should be accounted for?

We appreciate the Reviewer's suggestion to improve clarity for non-specialists and have revised the text accordingly (Supplementary Note 8, section: Neurodevelopmental conditions association of twelve FIS-associated non-DDG2P genes): "*We compared the observed number of de novo mutations in these genes to the number expected to occur by chance alone, based on a statistical model that accounts for the background mutation rate at each genomic position"*. To clarify, the cumulative per-site mutation rate refers to the summed mutation rate across all sites in a gene that can give rise to the relevant type of mutation (e.g. a protein-truncating variant), rather than at a single site. Indeed, $E_{i,G}$ should have the subscript G - this is now corrected.

Regarding the appropriateness of the Poisson test, we acknowledge that the model assumptions do not fully capture the true underlying biophysics of *de novo* mutation processes, including spatial autocorrelation. The mutation rate model we use (Samocha et al., Nature Genetics, 2014) estimates expected rates based on trinucleotide context and has been extensively validated and applied in large-scale studies of de novo variation (e.g., Deciphering Developmental Disorders (McRae et al., Nature, 2017) and Autism Sequencing Consortium (Fu et al., Nature Genetics, 2022). Crucially, rather than relying solely on the model's assumptions, we use synonymous *de novo* variants as an empirical control to verify calibration. If the model were substantially misspecified, or if violations of the Poisson assumption materially affected inference, we would expect spurious enrichment or depletion of synonymous variants, which we do not observe. This gives

us confidence that any enrichment detected in functional variant classes reflects a calibrated null model rather than artefact.

> Supplementary Material, p. 4: "Frequently Asked Questions (FAQ)" I am opposed to FAQs or digressions in the Discussion section like this. The proper place for this often non-scientific piece of editorializing is an author's website or some other platform clearly distinct from the paper itself. This is not the worst one that I've read, but it still does not belong. To single out just one objection I have to this particular one: I regard embryo selection as a grave evil, but the section about this seems more like ass covering than serious engagement of the issue. It states that there are "ethical concerns" but does not really say what they are. There follow some "technical challenges" regarding the effectiveness of embryo selection that can be disputed. Finally, there is a legitimate ethical point at the end about unintended deleterious consequences of pleiotropy, but this seems to contradict the earlier suggestions about limited effectiveness. It is pretty weak to say that there are ethical problems and then avoid mentioning any of the problems that concern those most strongly opposed to embryo selection, including the incentivizing of the mass destruction of embryonic life. Other problems might take some space to explain, but that one is brief and uncomplicated. Are pro-life views verboten at Nature Genetics? (Do you see what I mean about sticking to science?)

We agree that a FAQ section is better handled outside the permanent scientific record, and we have therefore removed the FAQ from the Supplementary Material and moved it to an external website (https://github.com/dmvandenberg/fluid-intelligence-imputation) where it can be revised and updated as a dynamic entity. We have also revised the FAQ to be more tightly focused on scientific interpretation and limitations of imputed phenotypes. We also revised the embryo-selection answer to make the scientific points more internally consistent (including clarifying how limited (non-zero) predictive power could still entail unintended trade-offs via pleiotropy, and that the expected future gains in predictive power would increase these trade-offs).

We note that the FAQ is focused on scientific clarification for non-specialist readers. Where it touches on potentially sensitive applications (including embryo selection), the goal is to pre-empt misinterpretation by clearly stating the study's scope and methodological limitations (for example, the ancestry restriction and the challenges of cross-population generalization), rather than to provide a broader ethical discussion on embryo selection which we feel is beyond the scope of this paper.

> Supplementary Material, pp. 12-13: Why does controlling for the *number* of singletons carried by an individual address fine-scale population structure? I can see why all those in the whole population carrying a *particular* variant observed as a singleton in a sample are probably related or come from the same locale. But why are those who carry similar totals of singletons more likely to come from the same fine-scale stratum? If different strata have expanded at different rates in the very recent past, then the total number of singletons might distinguish the strata. Is this the reason? Some readers might want to know more about this.

The number of singletons carried by an individual serves as an albeit crude proxy for fine-scale population structure that is not fully captured by principal components. It is well established that lower-frequency variants are more informative about recent population structure than common variants (e.g., Mathieson & McVean, PLoS Genetics, 2012), because they tend to arise more recently and are therefore more geographically localised. Individuals carrying an unusually high number of variants that are very rare/observed only once amongst the whole of UKB may have subtly more distant ancestries than the majority of participants, reflecting recent demographic differences across fine-scale population strata. Controlling for singleton count thus helps to account for residual stratification that PCs derived from common variants may miss. We added this reasoning to the text (Supplementary Note 7; Exome-wide burden analysis) as further clarification for the sensitivity analysis: "*As proxy for this fine-scale structure, we added the number of singleton synonymous variants exome-wide as a covariate, since individuals carrying an unusually high number of singletons relative to the sample may have subtly more distant ancestries than the majority of participants; we use synonymous variants as they are likely to be largely functionally neutral and hence not associated with the phenotype other than through population stratification*."

Supplementary Material, p. 13: “SKATO” -> “SKAT-O”

We have modified the spelling of SKATO to SKAT-O throughout.

Supplementary Material, p. 13: “encoding an axon-binding receptor” -> “encoding a receptor expressed in the growth cones of extending axons”

We thank the Reviewer for improving the description of *ROBO2* function. We have modified the text with this description.

Supplementary Material, p. 31: “the quality control of Whole exome sequencing data was described in the previous paper” Why is “Whole” capitalized? And what is the previous paper?

We have modified the spurious capitalization of the “w” and added a reference to the previous paper.

REFERENCES

McDonald, R. P. (2011). Measuring latent quantities. Psychometrika, 76, 511-536.

Tan, T. et al. (2024). Family-GWAS reveals effects of environment and mating on genetic associations. medRxiv.

Tucker-Drob, E.M., Briley, D.A., & Harden, K.P. (2013). Genetic and environmental influences on cognition across development and context. Current Directions in Psychological Science, 22, 349-355.

Young, A. (2022). Mendelian imputation of parental genotypes improves estimates of direct genetic effects. Nature Genetics, 54, 897-905.

Deciphering Developmental Disorders Study. Prevalence and architecture of de novo mutations in developmental disorders. Nature 542, 433–438 (2017).

Fu, J.M., Satterstrom, F.K., Peng, M. et al. Rare coding variation provides insight into the genetic architecture and phenotypic context of autism. Nat Genet 54, 1320–1331 (2022).

Reviewer #3

Summary. This paper imputes fluid intelligence for European-genetic-ancestry individuals in the UK Biobank and reports GWAS and exome-based rare-variant tests on a combined sample including both individuals with measured and imputed fluid intelligence. The imputation increases the available sample size from ~270,000 individuals (the sample size used in Chen et al., 2023, *Nature Genetics*) to >450,000 individuals. The paper explores several different imputation strategies, aiming to find a strategy that generates an imputed variable that satisfies several criteria: it is similar to the measured variable in SNP heritability, heritability of direct effects, and genetic correlation with two previous GWAS of cognitive ability and (as negative controls) with height and a previous GWAS of the “non-cognitive” component of educational attainment. Another criterion is a genetic correlation between direct effects and non-transmitted coefficients (coefficients on the parental genotypes) close to zero, since negative correlation likely reflects ascertainment bias. The paper settles on using average fluid intelligence scores across all occasions when it was measured and imputing missing values using a subset of 82 (out of 154) variables that have a stronger genetic correlation with fluid intelligence than with the “non-cognitive” component of fluid intelligence. The imputation accuracy is $r = 0.46$, compared to a retest reliability of approximately

0.6, and the SNP heritability of the imputed phenotype is 0.16, compared to ~0.20 for the measured variable. The paper finds that mega-analysis of the imputed with the measured variables improves power for all subsequent analyses. The main results come from the rare-variant analysis, which identifies 26 genes (Table 1), compared to 18 previously found by Chen et al., with effect sizes in the range ~0.25-1 SDs. In analyses in two (much smaller) validation cohorts, ALSPAC and MCS (each with ~5,000 individuals with exome data), the gene results replicate in aggregate (though there is not enough power to test specific genes) and are associated with lower childhood cognitive ability—though not necessarily with a diagnosed intellectual disability.

Evaluation. The core idea of this paper—imputing fluid intelligence in order to increase power for rare-variant analysis—is creative and important. The results are valuable and interesting. The analyses and discussion are thoughtful. The authors also admirably take on the responsibility of writing a General Lay Summary and FAQs in order to communicate responsibly with non-academic audiences.

The paper has two main weaknesses. First, the imputation procedure (despite all the thought and care that went into it) comes across as ad hoc, and it isn't clear to what extent we should be concerned about biases that it may introduce into the analysis. Second, it is difficult to understand from the paper exactly how the imputation was done, which makes it even harder to evaluate.

We thank the reviewer for the thoughtful evaluation and kind summary of our work. In response to the concerns raised, we have substantially revised the manuscript and Supplementary Information, including, for example, adding a new Supplementary Note that provides a formal theoretical framework to motivate the imputation approach and clarify potential sources of bias, and revising the main text to describe the imputation procedure more clearly and concisely. We respond to the reviewer's detailed comments point-by-point below.

1. I think the paper could be more persuasive and influential if it develops some theory underlying (at least the goals of) the imputation and proposes a systematic procedure for researchers who wish to impute phenotypes for the purpose of genetic analysis. In case it's helpful, I'll write out here what the paper led me to think about.

In the additive model, the measured phenotype can be written

$$y_m = g_m + \varepsilon_m,$$

and the imputed phenotype can be written

$$y_i = g_i + \varepsilon_i,$$

(in both cases omitting subscripts for individuals) where g denotes the additive genetic factor and ε denotes non-genetic factors. We can always decompose g_i as $g_i = g_m + \eta$, where the genetic component of the imputation error, η, is uncorrelated with g_m by construction. Thus, $y_i = g_m + \eta + \varepsilon_i$. In order for genetic analysis of y_i to be unbiased for discovering genetic causes of y_m, we need the composite error, $\eta + \varepsilon_i$, to be uncorrelated with g_m. In practice, what we can try to get evidence about is whether each component of the composite error is (separately) uncorrelated with g_m.

To test whether η is uncorrelated with g_m, we could take the individuals with measured phenotype values, impute their phenotype values, and calculate $y_i - y_m = (g_i - g_m) +$ $(\varepsilon_i - \varepsilon_m)$. Then, the genetic correlation of $y_i - y_m$ with y_m is an estimate of the correlation between η with g_m.

The assumption that ε_i is uncorrelated with g_m would be violated if there is gene- environment correlation (such as population stratification) where the relevant genetic component is g_m and the relevant environmental component is ε_i. Under the assumptions of LD score regression, the LD score regression intercept from a GWAS of y_i could provide evidence on whether ε_i is uncorrelated with g_i, which would imply that ε_i is uncorrelated with g_m if η is uncorrelated with g_m. Within-family analyses that control for parental genotypes would ensure that ε_i is uncorrelated with g_m, since genotypes are randomly assigned conditional on parental genotypes.

I think the paper would be stronger if it made clear how the tests of imputation quality that it conducts are related to a theoretical framework for what imputation is trying to achieve. If it turns out that the authors haven't conducted the right tests, then they should reconsider which tests they use.

We thank the reviewer for this very helpful suggestion. In the revised manuscript, we have added a new Supplementary Note 2 "Theoretical Framework for Interpreting Genetic Analyses of Observed and Imputed Phenotypes Subject to Measurement Error and Ascertainment Bias", which develops the formal framework the reviewer describes and ties it directly to the diagnostics we use in the paper.

Briefly, in this Supplementary Note we introduce a latent continuous trait, Y, with an additive genetic component G and a non-genetic component E, so that Y = G + E. Measured fluid intelligence (FI) scores are modeled as

$Y_m = Y + \varepsilon_m$,

where ε_m represents measurement error of the FI instrument. Imputed scores are modeled as

$\hat{Y} = f(W) + \delta$,

where W denotes the auxiliary variables used for imputation and δ is the prediction (imputation) error.

For individuals without a measured score (S = 0), our key assumption (Assumption 1 in the Supplementary Note) is that the imputation targets the latent trait Y and that the imputation error is orthogonal to genotypes within the imputed group:

$E[\hat{Y} \mid W, S = 0] = E[Y \mid W, S = 0]$, and
$Cov(\delta, X \mid S = 0) = 0$,
where X denotes genotypes. In the reviewer's notation, G corresponds to g_m, and the composite error term $\eta + \varepsilon$ in their decomposition plays the role of δ. The requirement that genetic analysis of $\hat{Y}$ be unbiased for discovering the genetic causes of the measured phenotype is therefore exactly the requirement that this composite error $(\eta + \varepsilon)$ be uncorrelated with the additive genetic component g_m.

We then show how our empirical tests map onto this framework:

Endogenous selection and mega-analysis

To address the concern that the subset of people with the measured FI score (S=1) being non-random may induce collider bias, we explicitly model endogenous selection driven by the latent trait Y and show how this can distort genetic effects within the measured and unmeasured groups. We then prove Theorem 5 ("Mega-analysis consistency under endogenous selection"), which constructs the pooled ("mega-analysis") phenotype:

$Y^* = Y_m$ if S = 1 (measured)
$Y^* = \hat{Y}$ if S = 0 (imputed).

Theorem 5 shows that, under Assumption 1 and standard conditions on measurement error, the pooled regression of Y* on genotypes has population limit:

$\text{plim}\ \hat{\beta}_p^{mega} = Cov(X_p, Y) / Var(X_p)$,

i.e. the same per-SNP effect that would be obtained if the latent trait Y were observed for all individuals. This result holds even when selection into S = 1 is, for example, a monotone increasing function of Y, which can generate collider bias within the measured subsample. In the reviewer's notation, this theorem shows that if the imputation error term $(\eta + \varepsilon_i)$ is uncorrelated with g_m, then the mega-analysis using Y* is asymptotically unbiased for the genetic causes of the underlying trait.

Given the endogenous selection into measurement for FIS, we believe that masking the phenotype in a subset of measured individuals and imputing it, as the reviewer suggested, would be difficult to interpret since we expect the estimates of the genetic associations in this selected group to be biased. Instead, we proposed alternative tests to assess whether the estimated effects obtained from Y* capture the intended effects we aimed to measure on unobserved Y, as described below.

Expected LDSC behavior and cross-trait genetic correlations

We next derive implications for LD score regression (Section 10 and Theorem 6). Under the same assumptions, namely that the imputation sufficiently targets Y and adds only genotype-orthogonal noise, we show that:

> (i) On the observed scale, the marginal per-SNP effect for Y* equals that for Y (this follows from Theorem 5).
> (ii) On the unit-variance scale used by LDSC, standardization rescales the per-SNP coefficients by a factor that depends only on Var(Y) and Var(Y*). This affects observed-scale SNP heritability but not the underlying per-SNP effects.
> (iii) For any external trait T, the genetic covariance and correlation are preserved:
> $\rho_g(\hat{Y}, T) = \rho_g(Y, T)$ and $r_g(\hat{Y}, T) = r_g(Y, T)$,
> while the observed-scale heritability rescales by Var(Y) / Var(Y*).

This derivation connects directly to our empirical checks in the main text. When we move from measured FI to the combined FIS phenotype, we observe exactly this pattern, namely, a modest reduction in observed-scale SNP heritability (from approximately 0.20-0.23 to 0.16), but that combined FIS and measured FIS are very similar in their profile of genetic correlations with external cognition GWAS, with negative control traits (height and the "non-cognitive" component of educational attainment), and with a wide array of other traits. In light of Theorem 6, that stability of the r_g profile is exactly what we would expect if imputation has added mainly genotype-orthogonal noise rather than a new genetically correlated construct.

Link to the reviewer's proposed tests and our existing diagnostics

The framework also clarifies how our existing diagnostics speak to the components of the composite error highlighted by the reviewer:

- The comparison of imputation accuracy to test–retest reliability, via Theorem 1, tells us how strongly $\hat{Y}$ is correlated with the latent trait Y relative to pure instrument noise. Intuitively, this is a check that the "genetic part" of the imputation error is small.

- The stability of LDSC cross-trait genetic correlations (r_g) with respect to cognitive and non-cognitive external traits is, by Theorem 6, a direct test that the non-genetic component of the imputation error (ε in the reviewer's notation) is not introducing new gene-environment correlation.

General testing procedure for imputation in genetic studies

We have added references to this derivation in the Supplementary Note 2 throughout the main text (e.g., p7), stating "*The genetic correlation profile of combined FIS was highly similar to that of the COGENT GWAS. Genetic correlations with height and NonCogEA consistent with those found with the COGENT GWAS (Figure 1C). As we show in Supplementary Note 2, this is a necessary condition if the imputation is targeting the same underlying latent genetic construct as measured intelligence.*". This framework converts our previously informal choices into an explicit and generalizable procedure for constructing and validating imputed phenotypes for genetic analysis:

1. Target the latent trait Y rather than instrument-specific artifacts (Assumption 1).

2. Combine observed and imputed values in a single mega-analysis regression of Y* on genotypes, using heteroskedasticity-robust standard errors.

3. Treat LDSC cross-trait genetic correlations as specificity checks that should remain stable, even as observed-scale SNP heritability attenuates.

We hope these additions address the reviewer's concerns about the theoretical underpinnings of our approach and provide a clear template that other researchers can adopt when imputing phenotypes for genetic analysis.

2. Relatedly, I think the paper should make clearer the rationale underlying the tests it reports. In case they're helpful, here are my thoughts on how the tests in the paper relate to the theoretical framework.

If η is classical measurement error that is uncorrelated with all other variables, then the condition that η is uncorrelated with g_m will hold. I interpret the authors' comparisons between the genetic correlation between y_m and some other variable (cognitive ability, height, the "non- cognitive" component of educational attainment), $y_0 = g_0 + \varepsilon_0$, and the genetic correlation between y_i and y_0 as implicitly trying to get at this (stronger than needed) assumption that η is classical measurement error. Since $Cov(g_i, g_0) = Cov(g_m, g_0) + Cov(\eta, g_0)$, the genetic covariances $Cov(g_i, g_0)$ and $Cov(g_m, g_0)$ will be equal if η is classical measurement error that is uncorrelated with all other variables. The genetic correlations will be equal if g_i and g_m have equal variances, which requires that η has zero variance (i.e., $\eta = 0$).

The analyses of how much lower the heritability of g_i is relative to g_m are relevant to assessing the variance of η and the statistical power of genetic analysis of the imputed phenotype.

We thank the reviewer for these thoughtful comments. As described in our response above, we now develop an explicit theoretical framework in the new Supplementary Note and use it to clarify the rationale for the tests of imputation quality that we report. Here we explain how our diagnostics relate to that framework and to the reviewer's notation.

Recall that in our framework, the latent trait of interest is Y, with additive genetic component G and non-genetic component E (Y = G + E). Measured FI scores are Y_m, and the imputed scores are $\hat{Y}$. For individuals without measurements (S = 0), Assumption 1 specifies that $\hat{Y}$ targets Y (so prediction error is mean-zero around Y conditional on the auxiliary variables), and that the imputation error is orthogonal to genotypes within the imputed group (no extra gene-environment correlation introduced by the imputation step).

Our Assumption 1 is precisely the requirement that this composite error is not systematically genetically correlated with g_m in the imputed group; it does not require that η has zero variance, nor that it is a classical measurement error in the strong sense of being uncorrelated with "all other variables."

Below we explain how the different tests relate to this framework.

Genetic correlation profiles with external traits

We agree with the reviewer that if one thinks in terms of the decomposition $g_i = g_m + \eta$, then:

- $Cov(g_i, g_0) = Cov(g_m, g_0)$ when η is genetically orthogonal to the genetic component of some other phenotype, g_0, and

- $Var(g_i)$ will equal $Var(g_m)$ only if η has zero variance, in which case the genetic correlations would also be exactly equal.

Our goal with the genetic correlation checks is not to assume η = 0 or that g_i and g_m have identical variance, but rather to test the weaker condition that the pattern of genetic overlap with external traits is preserved when moving from Y_m to the combined phenotype Y* (measured + imputed).

In the Supplementary Note 2 (Section "Evaluating Imputation via Genetic Correlations with LDSC"), we show that under Assumption 1, when we combine measured and imputed values into Y*, the following holds for any external trait T:

- The underlying per-SNP effects on the latent trait Y and on the mega-analysis phenotype Y* are the same.

- Measurement and prediction error (including η) inflate residual variance and hence reduce observed-scale SNP heritability for Y*, but they do not change the genetic covariance with T.

- After standardization to unit variance (the scale on which LDSC r_g analyses operates), the LDSC genetic correlation $r_g(\hat{Y}, T)$ is therefore expected to equal $r_g(Y, T)$, even though the heritability of $\hat{Y}$ is lower.

This result formalizes what we were informally trying to achieve with our genetic correlation checks (that the r_g profile between our combined FIS GWAS and an independent intelligence GWAS are stable and that the r_g with respect to a wide range of traits and particularly negative controls such as height and the "non-cognitive" component of educational attainment recapitulates the expected r_g as estimated with the Savage et al. GWAS) (Extended Data Figure 3, Supplementary Figure 3). These results are exactly what we would expect if the imputation has added genotype-orthogonal noise (increasing the variance of η) without introducing new systematic gene-environment correlation. In other words, we are not implicitly assuming that η has zero variance; we are using the observed stability of the r_g profile, together with the theoretical result above, as evidence that η is not loading on a distinct genetically correlated construct.

We have revised the Results and Discussion to state this rationale explicitly, and we now make clear that we expect heritability to attenuate when moving from measured FIS to combined FIS, but the cross-trait r_g pattern with cognition and negative controls to remain largely unchanged if the imputation is well behaved.

SNP heritability of the imputed vs measured phenotypes

We agree with the reviewer that comparisons of SNP heritability between Y_m and the combined phenotype speak to both the variance of the imputation error and the power of downstream analyses.

In the Supplementary Note (Section "SNP heritability under mixed measurement and imputation"), we derive the observed-scale SNP heritability for the mega-analysis phenotype Y*:

- $h_{SNP}^2(Y^*) = Var(G_{tagged}) / Var(Y^*)$,

where Var(Y*) includes variance from G, from E, from measurement error in Y_m, and from prediction error in $\hat{Y}$ (including η). Under Assumption 1, imputation affects only the denominator (by adding extra variance through prediction error) and not the per-SNP effects in the numerator. Thus:

- Any reduction in observed-scale h_{SNP}^2 when moving from measured FIS to combined FIS is expected and interpretable as added noise rather than as a change in the underlying genetic architecture.

- The magnitude of the reduction tells us how much effective signal-to-noise ratio we have traded off for the gain in sample size.

We see this pattern in our analyses. Combined FIS has somewhat lower h_{SNP}^2 than measured FIS, but the reduction is modest relative to the statistical power gained in downstream analyses, and the r_g profiles and within-family results remain stable.

We have revised the manuscript to explicitly link the tests of imputation quality (SNP heritability comparisons, genetic correlation profiles) to the assumptions and results in the new Supplementary Note, for example (p. 7): "*The genetic correlation profile of combined FIS was highly similar to that of the COGENT GWAS (Extended Data Figure 3; Supplementary Figure 3). Genetic correlations with height and NonCogEA consistent with those found with the COGENT GWAS (Figure 1C). As we show in Supplementary Note 2, this is a necessary condition if the imputation is targeting the same underlying latent genetic construct as measured intelligence.*". We hope these clarifications address the reviewer's questions about the rationale behind our validation strategy.

I'm not clear on the rationale underlying the authors' efforts to do the imputation using variables that have a stronger genetic correlation with fluid intelligence than with the "non- cognitive" component of fluid intelligence. In this analysis, g_m is decomposed into the sum of a "cognitive" component and a "non-cognitive" component. If the goal of the paper is to study genetic causes of g_m (as I think it is), then I'm not sure why we'd want to construct g_i to capture more of the "cognitive" component of g_m.

We appreciate that our rationale here was not sufficiently clear. The reviewer is correct that our ultimate goal is to study the genetic causes of g_m (the additive genetic component of measured FI), not to isolate "pure cognition" and discard all non-cognitive influences. However, there is a slight misunderstanding - we did not choose variables that have a stronger genetic correlation with fluid intelligence than with the "non-cognitive" component of fluid intelligence, but rather, variables that have a stronger genetic correlation with fluid intelligence than with the non-cognitive component of our initial ***imputed*** fluid intelligence variable. This is described in Supplementary Note 3 in the section "Refining imputation variables". This distinction is important. Our tests of this initial imputed fluid intelligence variable showed that it had a very high genetic correlation with the non-cognitive component of educational attainment (NonCogEA) (Supplementary Figure 4D - MegaFIS-vA). We were concerned that some of the auxiliary variables we were using had a genetic signal primarily aligned with NonCogEA, so the imputation procedure was beginning to approximate a broader "EA-like" trait rather than the FI trait indexed by the UKB test and independent intelligence GWAS. In other words, without some guardrail, the imputed phenotype was drifting toward a construct that mixes FI with non-cognitive features that are heavily represented in EA and in NonCogEA, potentially, increasing the genetic correlation with NonCogEA and height beyond what is typical for the FIS construct.

Thus, we isolated this non-cognitive component of the initial imputed fluid intelligence (NonCog-iFIS) using GWAS-by-subtraction and then calculated genetic correlations between NonCog-iFIS and the different variables we were including in the imputation. We then removed variables that showed a stronger r_g with NonCog-iFIS than with the Savage et al. intelligence GWAS. Intuitively,

- Variables whose genetic signal lines up more with the Savage et al. intelligence GWAS than with NonCog-iFIS contribute information that is more specific to a true measure of cognitive ability..
- Variables whose genetic signal is more strongly aligned with NonCog-iFIS than with the Savage et al. intelligence GWAS are likely to push the imputation toward the "non-cognitive" EA component that we explicitly want to avoid over-representing in a GWAS of FI.

This design choice does not change the scientific target from g_m to some abstract "cognitive-only" factor. Rather, it uses NonCogEA to regularize the imputation so that:

- $\hat{Y}$ remains anchored to the same genetic construct as Y_m and independent intelligence GWAS (as reflected in the r_g profile), and
- the imputation does not systematically introduce genetic signals that are primarily non-cognitive and potentially enriched for indirect and stratification effects.

Empirically, this filtering step moved us from imputation version 'MegaFIS-vA' to 'MegaFIS-vB', and we saw the pattern we would hope for if it is working as intended, yielding only a small drop in imputation accuracy (r from 0.52 to 0.46) but a substantial improvement in the specificity of the genetic correlations (e.g., reduced r_g with NonCogEA and height), as well as more similar population and direct-effect heritabilities and a near-zero direct–NTC correlation.

We have revised the manuscript's Supplementary Note 3 (previously Supplementary Note 2), Section *Refining imputation variables* to clarify that our variable-selection rule based on stronger genetic correlation with FI than with NonCog-iFIS is a way to ensure that the imputed phenotype remains anchored to the same latent FI trait Y "*To reduce the "non-cognitive" educational attainment signal being introduced by some of the imputation variables, and to more closely reflect the intended latent fluid intelligence trait, we refined the set of imputation variables based on whether their genetic signal aligned more strongly with cognitive ability*

than with non-cognitive correlates of intelligence". We hope these clarifications address the reviewer's questions about the rationale behind this strategy.

3. I'd like the authors to dig deeper into why mega-analysis of the measured and imputed fluid intelligence appeared to work better than meta-analysis, as described in Supplementary Note 3. As the authors explain there, meta-analysis has better theoretical properties because measured and imputed fluid intelligence have different means and variances. One worrisome possibility is that the apparent better performance of mega-analysis in terms of genetic signal is due to standard errors that are biased downward. This could occur because the imputation error causes heteroskedasticity: the variance of the error in a GWAS is higher for the imputed variable than the measured variable. If the GWAS assumes homoskedasticity, then the standard errors are likely to be biased downward. I am not sure if this worrisome possibility would also explain the other differences noted in Supplementary Note 3, but I'm at a loss in understanding what else could explain the differences (since under standard assumptions, if the phenotype were identical in the two samples, meta-analysis and mega-analysis should give identical results).

We thank the reviewer for pushing us to clarify this point. In the revision, we have substantially expanded the theoretical treatment of mega- vs meta-analysis in the new Supplementary Note 2. This extends the more informal arguments previously in Supplementary Note 4 (previously Supplementary Note 3).

Building on the framework described in our response above, we now:

1. decompose the within-group marginal SNP effects into three components:
   (a) the true marginal genetic effect on the latent trait Y (β_p),
   (b) an LD-tagging component that arises from correlations with other SNPs, and
   (c) a collider component induced by selection into being measured (S = 1) vs not measured (S = 0).

2. derive conditions under which inverse-variance weighted (IVW) meta-analysis does or does not cancel those components; and

3. show that, in the mega-analysis,when we regress the pooled phenotype Y* (measured plus imputed) on genotypes, the collider components cancel exactly in the covariance, so the mega-analysis slope converges to the same population limit as if the measured trait were observed on everyone.

Below we summarize what we learned and how it addresses the reviewer's concerns, including about heteroskedasticity and standard errors.

Why meta-analysis does not generally recover the ideal marginal effect

We define:

- Y_m = measured score when S = 1,
- $\hat{Y}$ = imputed score when S = 0, and
- $Y^* = Y_m$ for S = 1 and $\hat{Y}$ for S = 0 (the mega-analysis phenotype).

For each group S = s (measured or imputed), the marginal slope for SNP p satisfies:

- $\beta_p^s = \beta_p + \text{Collider}_p^s$,

Where Collider_p^s collects the bias induced by selection on the latent trait Y (through Cov(E, X_p | S = s)).

Under ideal conditions where both measured and imputed groups truly observe the same underlying phenotype Y and selection does not distort the joint distribution differently in S = 1 vs S = 0, these extra

terms would be similar across groups, and inverse-variance weighted meta-analysis would recover the same marginal effect as a mega-analysis.

However, in our setting, selection into S = 1 depends on Y and thus induces collider bias that differs across S = 1 and S = 0; and measurement and imputation introduce different noise structures in the two groups (different error variances, different replicate counts for Y_m, etc.).

In the new Supplementary Note (Section "Meta-analysis with collider bias"), we show that the IVW meta-analysis estimator under this type of ascertainment has no clear population identity. In other words, once selection and measurement/imputation differ across groups, IVW meta-analysis no longer has the nice property it has when both studies measure the same phenotype under the same selection mechanism. It becomes a weighted average of biased group-specific slopes and does not in general recover the marginal effect we care about.

Mega-analysis has preferable theoretical properties to meta-analysis

The key new result (Section "Mega-analysis versus meta-analysis" and the appendix "Exact cancellation of LD tagging in the mega analysis") is that mega-analysis behaves much better under the assumptions relevant for our setting. We find that even though LD structure and collider bias are distorted differently in the measured and imputed groups, the mega-analysis regression of Y* on X recovers the correct population marginal effects under Assumption 1, without imposing additional stability assumptions on LD or selection. The revised Supplementary Note now highlights this cancellation result and explains why, in this particular measured/imputed setting, mega-analysis has cleaner theoretical properties.

Addressing the concern about heteroskedasticity and standard errors

We fully agree with the reviewer that mega-analysis naturally introduces heteroskedasticity, and if one were to run a pooled OLS GWAS assuming homoskedastic errors and then use the usual model-based standard errors, this could indeed bias standard errors downward and inflate test statistics. Thus, we have now rerun all GWASs in Regenie with heteroskedasticity-robust "sandwich"standard errors (HC3). All qualitative results remained the same, including the advantage of mega-analysis over meta-analysis

This makes it unlikely that the advantage of mega-analysis we report is driven by downward-biased standard errors and instead likely reflects their different estimands. Mega-analysis targets $Cov(X_p, Y) / Var(X_p)$ for the latent trait Y (with robust SEs), whereas meta-analysis is a weighted average of group-specific slopes that include distinct LD-tagging and collider components.

Revised interpretation in the main text

Finally, we have revised the wording in the main text (and the corresponding Supplementary sections) to better reflect these theoretical insights. Rather than stating that meta-analysis is "theoretically preferable", we now state in the text (in Results section "Imputation of fluid intelligence scores", p. 7): "*We used mega-analysis rather than meta-analysis because theory predicts that mega-analysis of sufficiently well-imputed imputed phenotypes recovers population genetic effects even under endogenous selection into measurement (Supplementary Note 2). In contrast, inverse-variance weighted meta-analyses does not, in general, remove collider bias if present.*"

We hope that the expanded theoretical treatment, the explicit heteroskedastic-robust inference, and the clarification of the respective estimands address the reviewer's concerns about why mega-analysis "works better" in this context and why this is not an artifact of biased standard errors.

Does a PGI constructed from the mega-analysis have greater predictive power in independent samples than a PGI constructed from the meta-analysis?

We constructed two PGI identically in ALSPAC based on the mega-analysis and on the meta-analysis, using the methods described in the paper. We found that the mega-analysis-derived PGI had a substantially larger

standardized effect size on IQ in both the population and trio model (0.35 and 0.18) compared to the meta-analysis-derived PGI (0.30, 0.15) (Supplementary Table 9). This suggests the mega-analysis yielded effect sizes more concordant with the genetic liability for IQ, as predicted by the theory described above. We added a description of this analysis in Supplementary Note 4: "*In addition, the mega-analyses also demonstrated higher heritability estimates (Supplementary Table 6), and showed better out-of-sample prediction when PGI constructed with their summary statistics were used to predict IQ between and within families in an external cohort (Supplementary Table 9).*"

A related point is that we should expect heteroskedasticity in the imputed phenotype as a function of how many observations of fluid intelligence are averaged (the more observations, the lower the variance). I also worry that, if unaccounted for, this heteroskedasticity can bias the standard errors downward.

We thank the reviewer for raising this valid concern. We have now changed the GWAS method we employ to Regenie, where we implemented robust standard error estimation which produces valid standard error estimates under a heteroskedastic error structure. All downstream conclusions were qualitatively unchanged. Specifically, we continued to find that including imputed FIS increased lead SNP discovery relative to measured FIS, and significantly decreased r(direct,NTC), while retaining h^2 comparable to measured FIS. There was a modest decrease in the number in lead SNPs compared to our previous GWAS with fastGWA (550 for imputed FIS with Regenie compared to 590 with fastGWA), consistent with slightly downward biased SEs in the previous GWAS, but all substantive results remain unchanged. Furthermore, the relative performance of the various imputation approaches remains the same, with the "average" approach yielding the best results.

4. To help clarify how exactly you selected the imputation procedure you use, I'd suggest moving some of the material from Supplementary Note 2 to the main text.

We thank the reviewer for raising this point. Unfortunately there is not enough space in the main text to go into this in detail, but we have added a short description of the steps we went through to optimize our imputation, in the Results (page 6-7):"*To arrive at a robust imputation strategy, we went through several iterations. We started with refining our set of imputation variables, beginning with 154 variables phenotypically correlated with intelligence which were then subsetted to 82 variables whose genetic correlation profile was more aligned with intelligence than with the residual non-cognitive signal observed in the first imputation iteration (see Methods). Then, we evaluated three approaches to imputing FIS (Supplementary Figure 5), namely through imputing FIS using the earliest FIS measure for observed individuals, the average measure for observed individuals, or all available FIS measures. These approaches were evaluated using imputation accuracy, SNP heritability (population-based and within-family), genetic correlations with external traits, and the correlation between direct effects and non-transmitted coefficients. In Supplementary Note 2, we outline the theoretical framework formalising what our phenotype imputation is trying to achieve, and show how these evaluation metrics relate to this framework.*."

5. I think it's great that the authors wrote a FAQ for this paper. It's very nicely done, I have only a few suggestions:

We thank the reviewer for the positive assessment of the FAQ and for these helpful suggestions.

Following Reviewer #2's comments and the editor's guidance, we did move the FAQ from the Supplementary Material to an external website (https://github.com/dmvandenberg/fluid-intelligence-imputation), where it can be updated as a dynamic resource.

I suggest mentioning very early on (and in the General Lay Summary) that the study is restricted to European genetic ancestries, so as to pre-empt concerns about group comparisons.

We now state early in the General Lay Summary that analyses were restricted to UK Biobank participants of genetically inferred European ancestry.

> I like the terminology “polygenic index” that you use in the main text, but in the FAQ, you use “polygenic score.” Why not use “polygenic index” in both?

We have now harmonized terminology by using “polygenic index” consistently throughout the manuscript and accompanying materials.

> I am surprised that you don’t think there are clinical applications of the rare-variant results. I would have thought an effect size as large as 1 SD would be enough for, say, screening young children into an assessment for special needs. It’s not clear to me why the fact that not everyone with these variants have been diagnosed is a reason not to use the variants clinically.

We wrote the section about the clinical relevance of the rare variant results in consultation with clinical genetics colleagues. It would be very unorthodox for a gene to be used to identify putative Mendelian diagnoses purely on the basis of rare variant associations with a quantitative phenotype in UK Biobank; normally, stringent direct evidence for that gene’s association with a rare, severe condition is required. This might include a combination of evidence such as statistical enrichment in clinical cohorts, case series (including familial segregation of the suspected pathogenic variant), and functional evidence. While we agree that, if found in a child with intellectual disability who has unaffected parents, a *de novo* protein-truncating variant in one of the genes we have found would seem a good candidate to contribute to the phenotype, the case is much less clear for inherited variants. The relatively modest effect size of these variants, compared to other known Mendelian variants that cause severe intellectual disability and are frequently observed *de novo*, implies that they will be enriched for being inherited (often from clinically unaffected parents) rather than occurring *de novo* in clinical cohorts. In practice, the interpretation of such inherited variants is extremely challenging. The field is also some way away from screening children for variants where there is no established clinical intervention for a special needs assessment on the basis of their genetics (rather than on their actual phenotype), and the ethics of this are likely to be highly contentious. We have added the following sentence to the FAQ at the end of this paragraph: “*The evaluation of penetrance of these variants for neurodevelopmental conditions needs to be evaluated in very large, minimally ascertained cohorts (e.g. population-wide birth cohorts) to inform their interpretation in clinical contexts*.”

> 6. Typo in General Lay Summary, line 58: “caully”

Thank you, we have corrected this typo.

Editor

Your revised Article "Imputation of fluid intelligence scores reduces ascertainment bias and increases power for analyses of common and rare variants" has been seen by the original referees. You will see from their comments below and attached that, while they find the work substantially improved, they have raised a few ongoing concerns. We remain interested in the possibility of publishing your study in Nature Genetics, but we would like to consider your response to these points in the form of a further revision before we make a final decision on publication.

We therefore invite you to further revise your manuscript taking into account all of the reviewers' comments. Please highlight all changes in the manuscript text file.

Thank you for the opportunity to further revise our manuscript. We are grateful to the reviewers for their continued engagement with our work, their kind words regarding the quality of the manuscript, and their constructive feedback, which has further strengthened the paper. Below, we address each of the remaining points raised by the reviewers and we highlight all corresponding changes in the revised manuscript.

Reviewer #1

In this revision, the authors have responded thoroughly to the responses of the reviewers and made substantial changes, including writing a very careful new supplementary note describing the theory motivating their imputation procedure. In my previous review, I raised two major concerns. First, I worried that their imputed phenotype was contaminated by the phenotypes they used for imputation in a way that affected the interpretation of their results. Second, I asked why their approach would be preferable to an approach like POP-GWAS, which accounts for such potential contamination. I also raise a handful of small concerns. The authors have done an excellent job addressing my second major concern and all of my small concerns; however, I remain somewhat concerned about contamination.

To be clear, the revisions have substantially improved the manuscript's treatment of the imputed phenotype, and I appreciate the care the authors have taken to engage this issue. In particular, I found the new Discussion language and Supplementary Note 2 helpful. The supplement now provides a clear framework for why mega-analysis of measured and imputed phenotypes could, under explicit assumptions, reduce ascertainment-related bias relative to relying only on the subset with directly measured FIS. I also appreciate the additional empirical work the authors have done to reduce concern about construct drift, including refinement of the imputation variable set, discussion of why an earlier version appeared too "EA-like," and the use of external genetic-correlation patterns and related diagnostics to evaluate whether the final combined FIS remains aligned with a cognitive phenotype. Together, these additions make the paper considerably stronger and make me more comfortable that the imputation is not simply producing an arbitrary proxy phenotype.

We thank the reviewer for these words of endorsement and the careful evaluation of our revised manuscript.

That said, I continue to have an important residual concern about how this limitation is framed across the manuscript. My overall instinct is that the imputation is likely largely reasonable and that the combined FIS is probably useful for discovery. However, I remain worried by the apparently substantial difference in the genetic correlation with educational attainment between observed FIS and combined FIS. Even if the overall genetic-correlation profile is broadly similar, and even if some negative-control traits look reassuring, the EA result still raises the possibility that at least some of the newly discovered signals may reflect variants associated with educational attainment through pathways that are not purely cognitive.

My concern is not that the authors have ignored this issue entirely; on the contrary, I think they have done high-quality work to identify it, reduce it, and discuss it more candidly than in the original version. Rather, I think the remaining uncertainty is not yet woven consistently enough through the manuscript. In several places, the paper still reads as though combined FIS can be treated largely as though it were a directly measured intelligence phenotype. For example, the abstract headlines the higher number of independent SNP associations, improved prediction, and new genes found from a rare variant analysis, but has no discussion on the limitations of these results. Even in the main text where the genetic correlations between combined FIS and EA (non-cognitive) are presented, the authors don't point out this discrepancy, instead saying that "Genetic correlations with height and NonCogEA consistent with those found with the COGENT GWAS." Instead, an acknowledgement of the limitations is only found in the Discussion and Supplementary Note 2, which support a more qualified interpretation: combined FIS is a pragmatically useful proxy phenotype that may improve on the selected observed-FIS sample, but the magnitude of the remaining construct-drift problem has not been fully quantified. I think the paper would be improved if this limitation were integrated more explicitly throughout the rest of the paper,

especially when presenting downstream discoveries and biological interpretation, rather than being concentrated mainly in the limitations section.

We agree that the remaining uncertainty around contamination could have been woven more consistently throughout the manuscript. We added appropriate nuance at several points in the abstract and main text:

p. 1, line 25: Extensive validation suggests that the imputed phenotype genetically resembles measured FI and reduces ascertainment bias, though some non-cognitive signal may remain."

p. 8, line 226-231: "However, we note that combined FIS showed slightly higher genetic correlation with EA, NonCogEA, and household income than Observed FIS (Figure 1C), so we cannot rule out that some residual non-cognitive signal (e.g., SES) was introduced through imputation. Nonetheless, the close correspondence with COGENT suggests that combined FIS represents a reasonable approximation of the cognitive construct measured by intelligence GWAS, and our results below suggest it is useful despite its limitations."

p. 10 line 257-260: "We acknowledge that these findings could be driven at least partly by 'non-cognitive' signal we may be capturing through the imputation, which could still reflect other brain-related traits such as personality."

p. 11 line 304-307: "While we cannot fully rule out that some of these associations arise through non-cognitive pathways, the strong enrichment for genes with prior evidence of involvement in intellectual disability suggests that at least many of these discoveries are driven by cognitive signal."

p. 12 line 335-336: "We sought to assess whether the newly discovered genes nonetheless carry genuine cognitive signal by testing them for association with measured cognitive ability in two birth cohorts (ALSPAC, N=5,283; MCS N=5,621)."

Alternatively, the authors should be more explicit in their manuscript why bias due to contamination is unlikely to materially affect interpretation of the new findings. For example, the new supplement suggests that if the imputation is uncontaminated by other phenotypes, then the patterns of rg should be the same. This is largely true with the exception of EA, EA (non-cognitive), and household income. Given that this is a violation of the assumptions in the Supplement, is it possible to infer how much this might affect downstream results and how that compares to the bias from ascertainment that the authors focus on? Even if this cannot be fully resolved empirically, I think the manuscript should make this tradeoff more explicit and sustain that framing throughout.

We thank the reviewer for this suggestion. While we agree that the potential for bias due to contamination requires careful consideration, we would like to elaborate on the elevated genetic correlations with EA, EA (non-cognitive), and household income. The NonCogEA GWAS was derived by "subtracting" the genetic signal of a meta-analysis of UKB FIS (N = 222,543) and COGENT (N = 35,298) from EA; hence, in this UKB measured FIS is the primary driver of the "cognitive" signal used in that GWAS. As a result, observed UKB FIS has a genetic correlation of ~0 with NonCogEA, since the GWAS-by-subtraction approach by design assumed that the Cog and NonCog components had a genetic correlation of 0. We note that COGENT has a genetic correlation of ~0.20 with NonCogEA, presumably reflecting the fact that it only made up 13% of the Cog GWAS. This suggests that not all of COGENT's signal was captured in the Cog GWAS, causing some to leak through into the NonCogEA GWAS. We observed that genetic correlations across a large range of traits obtained with combined FIS were highly similar to those obtained with COGENT - a Deming regression association was not significantly different than 1 (Extended Data Figure 3, Supplementary Figure 3). We interpret this as evidence that combined FIS is likely not substantially violating the assumptions outlined in Supplementary Note 2. It is unclear whether the remaining

genetic correlation with NonCogEA is reflective of a technical artefact in the construction of NonCogEA or the importation of some noncognitive signal to our GWAS. Nonetheless, the downstream analyses do suggest that combined FIS is a genetically meaningful and useful phenotype to study. We acknowledge, however, that the ratio of cognitive to non-cognitive signal cannot be fully resolved empirically. We added the following sentence: (p16. 450-453) to the Discussion to highlight this: "As the precise ratio of cognitive to non-cognitive signal cannot be fully resolved empirically, it remains unclear whether elevated genetic correlations with NonCogEA reflect a technical artefact in the construction of the NonCogEA GWAS or importation of truly non-cognitive signal."

Reviewer #2

The authors went to great effort, perhaps more than necessary, to address the questions and concerns of the reviewers. More authors have been added to the team, apparently for their expertise in matters raised by the reviewers, but I have also added authors to my papers in similar circumstances and see no problem with this. An extensive supplementary note has been added, demonstrating that if errors in prediction/imputation of the true score (as opposed to measurement) have mean zero conditional on the predictors and are uncorrelated with genotypes, then many desirable consequences follow. The premises here cannot be proved, but the consequences can be empirically checked, and they do more or less hold in this case.

We thank the reviewer for their careful reading and are pleased that the additional analyses and supplementary note were well-received.

Main p. 6, lines 155-157: "and resemble those of the direct genetic effects after accounting for residual population stratification with LDSC regression" This clause was in the original submission, but I now find that I do not understand it. Perhaps consider omitting.

As the second clause is redundant, we have omitted it from the manuscript.

Main p. 7, lines 219-222: This new sentence is missing a word.

Thank you, we rearranged the sentence (p. 7, line 219-222) to: "The genetic correlation profile of combined FIS was highly similar to that of the COGENT GWAS (Extended Data Figure 3; Supplementary Figure 3), including consistent genetic correlations with height and NonCogEA, showing that this necessary condition of the imputation has been met (Supplementary Note 2)."

Main p. 29, line 748: There should be a period after "GWAS," before the next sentence beginning with "SNP."

Thank you, this has been fixed.

Main p. 35, line 950: This Tan et al. paper is a preprint on medRxiv, currently under review at Nature. Why is the journal given as Genetic and Genomic Medicine?

We thank the reviewer for catching this. The previous error was due to a bug in our referencing software. We have corrected the reference to: Tan, T. *et al.* Family-GWAS reveals effects of environment and mating on genetic associations. *medRxiv* (2024) doi: 10.1101/2024.10.01.24314703v3.

I have just noticed that there are inconsistencies between Extended Data Figure 3 and Supplementary Table 3. Some of the genetic correlations in the "Psychological/Social" category (e.g., frequency of friends/family visits) have opposite signs in these two "floats". So, either the figure is wrong or the table is wrong. The authors should double-check all of these genetic correlations.

We thank the reviewer for catching this. The apparent inconsistency arises because several traits in Extended Data Figure 3 use counterintuitive phenotype coding, where higher values correspond to less favourable outcomes. Affected traits are:

- Self-rated health
- Job satisfaction
- Family relationship satisfaction
- Friends relationship satisfaction
- Frequency of friend/family visits

For example, satisfaction traits (family relationship satisfaction, friends relationship satisfaction, job satisfaction) are coded on a scale where 1 = 'Extremely happy' and 6 = 'Extremely unhappy'. To aid interpretation, we flipped the sign of the genetic correlations for these traits in the figure so that positive values consistently reflect a more favourable outcome. We now flipped the signs for these traits in Supplementary Table 3 as well and added text to the figure description of Extended Data Figure 3 and below Supplementary Table 3.

Reviewer #3

I liked the previous version of this manuscript, and I believe the revised manuscript is much stronger. The authors have been highly responsive to my comments. I now understand much better how the imputation was conducted, why the authors tested different approaches the way they did, and the theoretical framework that underlies the imputation. My main comment on the revised manuscript is that it still does not make clear what theoretical assumptions are needed for the imputation method to produce unbiased estimates and, relatedly, what realistic violations of the assumptions a researcher should be worried about when evaluating whether to apply the approach in this paper. These issues have now been worked out in the new Supplementary Section "Theoretical Framework…" but a reader of the main text will still have little idea when and which assumptions are needed. I appreciate the space constraints, but the only reason a researcher should prefer using measured variables over imputed variables (a preference the authors now emphasize) is because the assumptions required for the imputation approach may be violated. Therefore, a reader of this paper should come away with an understanding of the tradeoff they are making: which additional assumptions do they need to swallow in exchange for the additional statistical power delivered by imputing the phenotype?

Thank you for this thoughtful summary and for the generous assessment of the revision. We agree with the core point. In the previous version, although the relevant assumptions had been developed in the new Supplementary Note 2, we had not made sufficiently clear which assumptions are needed for which results, nor had we made the empirical tradeoff transparent enough for readers deciding whether imputation is appropriate in their own setting.

To address this, we substantially revised the Supplementary Note so that the assumptions are now stated explicitly, numbered, and tied to the specific theoretical results that use them. In particular, we now distinguish between the core assumptions needed for the main pooled GWAS and LDSC

arguments, and the stronger assumptions used only in the selection subsection to obtain interpretable collider formulas.

We agree that the practical tradeoff should be clear to the reader. The revised note now emphasizes that the main additional requirement for pooling measured and imputed phenotypes is calibration of the imputed phenotype to the latent target trait, both at the level of the conditional mean and, for the pooled GWAS result, at the level of SNP-phenotype covariance in the unmeasured group. We also make clearer what kinds of violations are most concerning in practice, namely situations where the imputation imports genetically correlated non-target signal or where group-specific recentering or rescaling distorts between-group mean structure.

We also added text in two places in the main manuscript to make the assumptions and their role explicit. In the Results (p.6-7, line 173-187) we added the following:
"In Supplementary Note 2, we consider the circumstances under which such a strategy could, in theory, produce unbiased genetic effect estimates, and describe how it should be formally evaluated. In brief, we show that unbiased genetic estimates can be recovered through mega-analysis of observed and imputed values if two key assumptions hold: 1) that measurement error in the observed values is uncorrelated with genotype and 2) that the imputed values are calibrated to the latent trait in the unmeasured group, in the sense that i) on average they recover the conditional mean of true FI given the auxiliary variables, and ii) their covariance with each SNP equals the covariance that the latent trait itself would have with that SNP in the unmeasured group (Supplementary Note 2 and 4). Intuitively, the second condition requires that the imputation does not import genetic signals from the auxiliary variables that are not also present in the latent cognitive trait. While these assumptions cannot be directly tested, we demonstrate that a direct consequence of them being met is that the genetic correlation profile obtained from a GWAS of the resulting mega-analyzed GWAS is unchanged relative to that obtained from an independent GWAS of the desired phenotype."

Additionally, in the Limitations in the Discussion (p. 17, line 464-476) we added:
"Importantly, many of the conclusions depend on two assumptions which cannot be proven to hold definitively, namely that classical measurement error in the FI test is uncorrelated with genotype, and that the imputed phenotype is calibrated to the latent trait in the unmeasured group, both in the conditional mean and in its covariance with each SNP. Additional assumptions are needed only in narrower parts of the analysis e.g., the directional interpretation of the direct-versus-NTC correlation as a marker of ascertainment bias relies on stronger assumptions about how selection distorts the joint distribution of genetic and environmental components; these are stated formally in Supplementary Note 2. Thus, researchers considering this approach in other settings should weigh the power gains against the plausibility of these calibration assumptions in their specific application, and should verify them empirically wherever possible by checking that external genetic correlation profiles are preserved."

We agree that this is the relevant tradeoff. The revised text is intended to make clear that imputing the phenotype can increase power, but only under additional assumptions that do not arise when restricting analysis to measured values alone.

> I will focus the remainder of my comments on the new Supplementary Section "Theoretical Framework…" which—although it is extremely helpful—could still be substantially improved. My main suggestions regarding the new section are:

A. Make it clearer which assumptions are being used where. A standard way to do this is to number the assumptions, and then state, at the beginning of each formal result, which assumption numbers are being assumed.

We agree, and we revised the Supplementary Note accordingly. The assumptions are now explicitly numbered and separated by role. We also added an assumption-roadmap paragraph near the start of the note (top of page 3) explaining which assumptions are core for the main pooled GWAS and LDSC results and which are used only in the selection subsection to obtain interpretable collider formulas. In addition, the formal results now state their assumptions explicitly, so that it is clear what is needed for each theorem or corollary and what is not.

B. Relatedly, make the links between the assumptions/theoretical results and the empirical analysis more explicit. Some of the remarks do this, but there isn't enough of it. (After having read the new section, when I re-read the paper, I was still a bit lost about what assumptions were being made at each stage of the analysis. The summary at the end of the section ("Conclusions and Implications") provides some of these links but mentions only a few of the assumptions that underlie the theoretical results.)

We agreed with this point and revised the note to make these links much more explicit. We added a paragraph explaining the empirical role of each assumption set (top of page 3), and throughout the note we now state more directly which assumptions underlie which parts of the analysis. In particular, we clarify that the calibration assumption for the imputed phenotype underlies the pooled combined-FIS GWAS, the preference for mega-analysis over IVW meta-analysis, and the LDSC validation criterion based on preservation of the external r_g profile. By contrast, the stronger assumptions in the selection subsection are now stated explicitly as being used only to interpret the within-group collider patterns, including the direct-versus-NTC results, rather than to identify the pooled GWAS estimand itself. We also expanded the concluding discussion (Section 11) so that it reflects this division more completely.

C. Throughout, make it clearer whether (and where) you are making independence or orthogonality assumptions. (See minor comments 7 and 9 below.)

We revised the note substantially on this point. In the earlier version, some statements used conditional-independence notation in places where weaker conditions were actually sufficient. We now make that distinction explicit. In particular, the selection subsection (Section 6) has been rewritten so that the general decomposition result is stated using only the existence of second moments, and the simplification step is stated in terms of conditional first- and second-moment invariance given $(Y,\mathbf{C})$, rather than conditional independence. We retain stronger Gaussian assumptions only where they are actually needed, namely for the closed-form collider expression and associated sign intuition. This revision was intended to make clear both where stronger assumptions are being used and where weaker moment conditions suffice.

D. Demarcate more clearly between formal results and commentary on the results.

We revised the structure of the Supplementary Note to separate these more clearly. Definitions, assumptions, theorems, corollaries, and proofs are now stated in a more self-contained way, while explanatory interpretation and links to the empirical analyses are placed in separate remarks. This was done throughout the note, but especially in the imputation and selection sections (sections 5 and 6), where the previous version mixed formal statements and commentary too closely.

> E. Minimize redundancy. Numbering the assumptions would help with this, since you wouldn't need to restate them.

We agree, and this was one of the motivations for restructuring the note. After numbering the assumptions, we were able to reduce repeated verbal restatement and instead refer back to the relevant assumptions directly in the formal results. We also trimmed several passages where the previous version repeated the same intuition in multiple places, while keeping brief remarks where needed to connect the theory to the empirical analysis.

> Here are more specific and more minor comments/suggestions:
> 1. Definition 1: Since $\mathbb{E}(\varepsilon!|Y) = 0$ implies $\mathbb{E}(\varepsilon!) = 0$ (by the law of iterated expectations), it is confusing to state both of them.

We revised the measurement-error set-up (section 2.2) to avoid redundant mean-zero statements.

> 2. Definition 2: The best predictor function for $Y!$ is $\mathbb{E}(Y|\boldsymbol{W}, S = 0)$. Any other unbiased predictor $f(\boldsymbol{W})$ would have a larger conditional variance. It isn't clear to me why you want the generality of allowing any unbiased predictor $f(\boldsymbol{W})$ rather than setting $Y - = \mathbb{E}(Y|\boldsymbol{W}, S = 0) + \delta$. I suggest either clarifying the reason for the generality or dropping it.

Thank you. We agreed with this point and revised the definition accordingly. In the current version, we no longer allow an arbitrary unbiased predictor f(W). Instead, we define the imputed phenotype relative to the conditional mean target, and use δ to denote the calibration error. We also moved the mean-calibration requirement out of the definition and into a numbered assumption (Assumption 2), so that it is clear which part is definitional and which part is substantive. This makes the exposition cleaner and aligns the notation directly with the target quantity relevant for the pooled GWAS and LDSC arguments.

> 3. Assumption 1:
>   a. δ was defined previously, as well as its conditional expectation equaling zero.
>   b. It's not clear whether "We additionally assume…" is meant to be part of Assumption 1. Similarly for "A weaker but sufficient condition…"
>   c. At some point, the assumption switches from being a statement of the assumption to being commentary. Make it clearer where that happens.

We agreed with all three parts of this comment and revised the section substantially.

First, we restructured the imputation set-up so that δ is introduced only as the calibration error in the definition of the imputed phenotype, while its mean-calibration property is no longer treated as definitional. Instead, calibration is now stated as a separate numbered assumption (Assumption 2).

Second, we removed the earlier ambiguity about what was formally part of the assumption and what was not. In the revised version, the assumptions are stated cleanly, and any stronger or weaker alternatives are discussed separately in remarks.

Third, we moved explanatory discussion out of the formal assumption statements and into separate commentary (Remark 1). This was done specifically to make the boundary between assumptions and interpretation clearer.

> 4. Before Theorem 1: I suggest changing "empirical correlation" to "correlation" since everything is about the population random variables.

Thank you. We made this change.

> 5. "Selection mechanism" subsection: S needs to be defined earlier since it is used earlier.

Thank you. We now define S earlier in the note (section 2.2) and use that notation consistently thereafter.

> 6. "Selection mechanism" subsection: A few things are confusing about the definition of "endogenous selection":
> a. It is not set off in a separate type the way other definitions are.
> b. The term does not appear to be used in the remainder of the text.
> c. It is stated as if equation (8) is the definition, but equation (8) just defines the notation; the definition is really that the h function is increasing in Y.
> d. The standard definition of endogenous selection would allow any kind of dependence of the h function on Y. It is not clear why it is being defined in this more specific way and whether that has any consequences.

Thank you. We revised this subsection substantially and no longer use the earlier term "endogenous selection." Instead, we now define phenotype-dependent selection explicitly in a formal definition and then separately define the monotone-in-Y case used for the directional collider discussion. This addresses the concerns, namely, the definition is now set off formally, the role of the equation is clearer, and the note now distinguishes between general dependence of selection on Y and the more specific monotone case, which is used only because it yields an interpretable sign result. We also clarify that the mega-analysis consistency result does not require monotonicity.

> 7. "Selection mechanism" subsection: Clarify "$S \perp (G, E)|(Y, \boldsymbol{C})$ (i.e., given $(Y, \boldsymbol{C})$ the act of selection itself contributes no further information about (G, E))." The symbol $\perp$ usually means orthogonal, but it is sometimes used mean independence; which is meant here? On the one hand, the analyses are linear models, so maybe all we need is orthogonality. On the other hand, the parenthetical "i.e." makes it sound like independence.

We agree that this notation was ambiguous and revised the subsection to avoid it. In the current version, we no longer use the symbol $\perp$. Instead, we state directly the weaker conditional first- and second-moment invariance conditions that are actually used for the decomposition result. This makes clear that the relevant step does not require a generic orthogonality notation or full conditional independence. Where stronger assumptions are used, we now state them explicitly in terms of conditional distributions or Gaussian structure rather than relying on the overloaded $\perp$ notation.

8. Proof of Theorem 2, first line: is the assumption $S \perp (G, E)|(Y, \boldsymbol{C})$ actually needed for the first claim? It looks to me like it follows just from the law of total covariance.

We agree. In the revised version, the first decomposition is now stated under existence of the relevant second moments alone, as a direct application of the law of total covariance. The additional assumption is used only for the simplification step that replaces conditional moments given (Y,C,S=1) with those given (Y,C).

9. Corollary 3: Same comment as #7 but regarding $(G, E) \perp \boldsymbol{C}|Y$.

We revised this in the same spirit. In the current version, we no longer express the relevant condition using (G,E)⊥**C**|Y Instead, for the closed-form corollary we state directly the stronger conditions that are actually needed, namely the Gaussian residual-structure assumptions and the requirement that conditioning on **C** does not change the conditional first two moments of (G,E) once Y is fixed.

10. Theorem 2 and Corollary 3: It is not clear to me where the normality assumptions are used in the empirical analysis. (The "Conclusions and Implications" section does not mention them.)

We clarified this point explicitly in the revised note. The stronger Gaussian assumptions are now confined to the closed-form collider corollary and are used only to obtain the simple expression and sign intuition for the within-group collider term. We now state this directly both in the selection subsection and again in the concluding discussion so that the scope of the normality assumptions is clear.

11. Equation 12: You should state that you are now switching from population random variables to estimators, and explain that $i = 1,2,\ldots, n$ indexes individuals in the sample.

We added this clarification immediately before introducing the mega-analysis estimator.

12. Remark 5: The reference to "implementation constraints" is cryptic. I suggest spelling out more of what you have in mind.

We revised this discussion to make the implementation issue explicit. In the current version, we now state directly that the concern is group-wise recentering or otherwise imposing a common group mean before pooling measured and imputed phenotypes. We then derive the resulting between-group bias term and explain how it depends on both phenotype mean differences and genotype-frequency differences between the measured and imputed groups.

13. Remark 5: I am confused by the claim that the bias is "typically negligible." I can see why that would be true if sample selection is related only to polygenic phenotypes, but what if it is related to residual population stratification? Couldn't the differences in genotype frequencies be relatively large in that case? In addition to clarifying this point, this is one place where it would be helpful to tie the discussion to the empirical analysis.

We agreed with this point and revised the remark substantially (it is now Remark 10, page 9). We no longer state that the bias is generically negligible and instead derive the bias term explicitly and make clear that its magnitude depends jointly on the phenotype mean difference between groups and on genotype-frequency differences between groups. As you suggest, this need not be small if measured and imputed groups differ because of residual population structure or other ancestry-correlated factors could lead to the difference in allele frequencies between S=0 and S=1 to be nonzero and thus influence the estimate if not properly accounted for. We therefore now tie this discussion directly to the empirical analysis, noting that in our application we mitigate this risk by restricting to a relatively homogeneous ancestry subset, adjusting for genetic principal components, and checking genetic correlations with a wide panel of traits and combined FIS are concordant with those obtained from COGENT and that the direct-versus-NTC correlation moves toward zero. We added a sentence to the Methods (p. 28, line 727-729) explaining this at a high level: "While this may in principle bias associations, the resulting per-SNP bias scales with allele frequency differences between measured and imputed individuals, which are likely negligible (see Supplementary Note 2, Remark 10 for more further discussion)."
